# Linearization of the Brevicidine and Laterocidine Lipopeptides Yields Analogues That Retain Full Antibacterial Activity

**DOI:** 10.1021/acs.jmedchem.3c00308

**Published:** 2023-04-18

**Authors:** Ross D. Ballantine, Karol Al Ayed, Samantha J. Bann, Michael Hoekstra, Nathaniel I. Martin, Stephen A. Cochrane

**Affiliations:** † School of Chemistry and Chemical Engineering, Queen’s University Belfast, Stranmillis Road, Belfast BT9 5AG, U.K.; ‡ Biological Chemistry Group, Institute of Biology, Leiden University, Sylviusweg 72, Leiden 2333 BE, The Netherlands

## Abstract

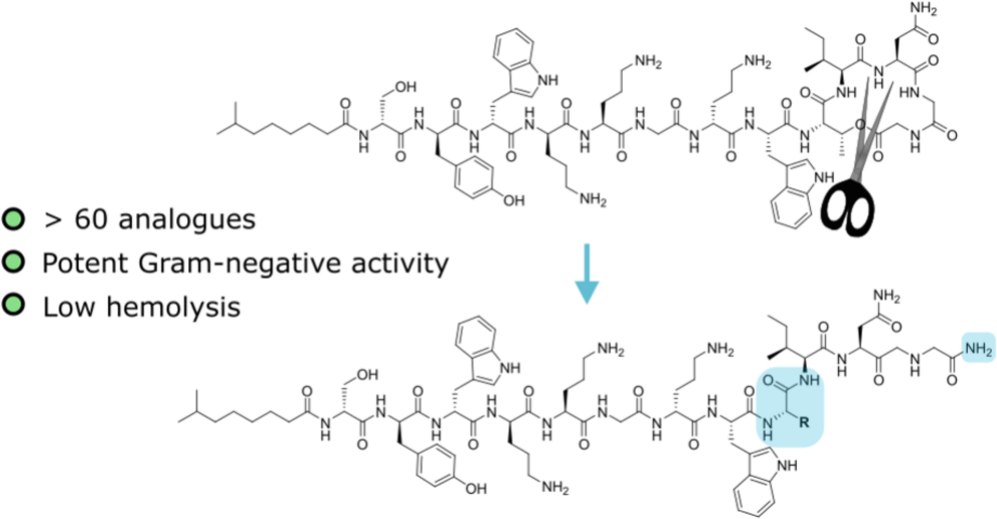

Brevicidine and laterocidine are macrocyclic lipodepsipeptides with selective activity against Gram-negative bacteria, including colistin-resistant strains. Previously, the macrocyclic core of these peptides was thought essential for antibacterial activity. In this study, we show that C-terminal amidation of linear brevicidine and laterocidine scaffolds, and substitution of the native Thr9, yields linear analogues that retain the potent antibacterial activity and low hemolysis of the parent compounds. Furthermore, an alanine scan of both peptides revealed that the aromatic and basic amino acids within the common central scaffold are essential for antibacterial activity. This linearization strategy for modification of cyclic peptides is a highly effective way to reduce the time and cost of peptide synthesis and may be applicable to other non-ribosomal antibacterial peptides.

## Introduction

Antimicrobial resistance (AMR) is a growing concern posing a threat to both global health and economics. Current estimations predict that by 2050, the number of deaths attributable to AMR will surpass the annual deaths caused by cancer.^1,2^ In this context, it is imperative that renewed efforts be made to discover and develop new antibiotics, in particular for the ESKAPE pathogens (*Escherichia coli*, *Klebsiella pneumoniae*, *Acinetobacter baumannii*, *Pseudomonas aeruginosa*, and *Staphylococcus aureus*) which represent the most important nosocomial infections within the United States.^3^ Non-ribosomal peptides (NRPs) represent a burgeoning class of antibacterial compounds with the potential to address resistance.^4^ Several classes of NRPs, such as the polymyxins, daptomycin, and vancomycin, are already in clinical use,^5^ and many more promising antibacterial NRPs have been discovered in recent years, including lysocin E,^6^ teixobactin,^7^ lugdunin,^8^ malacidin,^9^ and cilagicin.^10^ Although the structures of different classes of NRPs vary considerably, a significant majority contain one or more macrocyclic rings. These structural features complicate the chemical synthesis of NRPs, as additional orthogonal protecting groups and special cyclization conditions are required for their synthesis.^10−14^ This increases the complexity and cost of their chemical synthesis, which can be a barrier to clinical uptake.

Structure–activity relationship (SAR) studies are frequently performed on NRPs to optimize activity, stability, and/or cost of synthesis. They can also provide an insight into the mode of action. Alanine scans have already been performed on a number of NRPs, including daptomycin, tridecaptin A_1_ (TriA_1_), and polymyxins, to identify residues that are crucial for antibacterial activity.^15−17^ Additionally, side chain substitution analogues have been generated for laspartomycin C,^18^ and work on teixobactin has shown that *allo*-enduracididine 10 can be replaced with both Arg and Lys, with the resulting analogues retaining strong activity.^19,20^ Although many SAR studies have been performed, the linearization of cyclic NRPs is rarely reported.^21^ If this can be achieved without loss of activity, the cost of, and time required, for synthesis would be significantly reduced.

**Figure 1:**
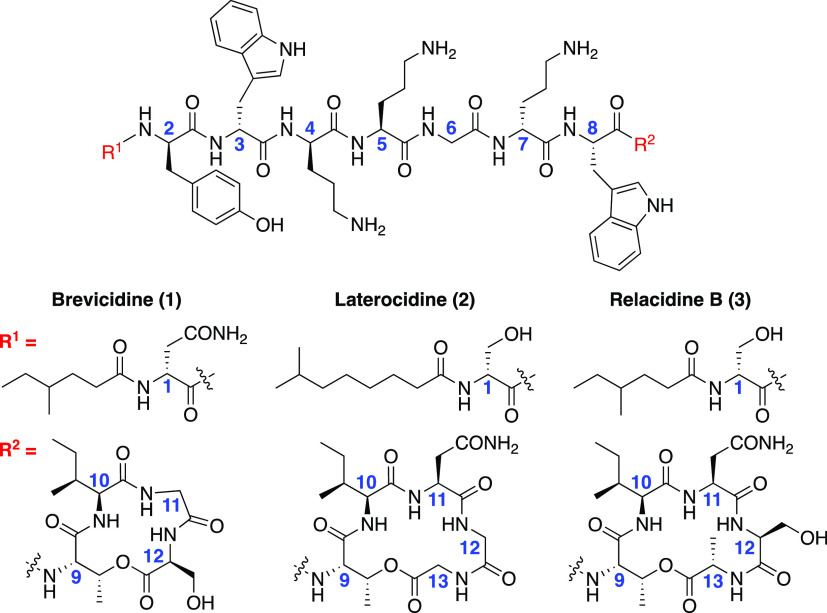
Structures of some known ornicidines: Brevicidine (**1**), laterocidine (**2**), and relacidine B (**3**).

In 2018, the NRPs brevicidine (**1**) and laterocidine (**2**) were discovered and found to have potent antibacterial activity against Gram-negative bacteria, including colistin-resistant *E. coli*.^22^ Structurally related analogues, relacidine A and B, were subsequently discovered in 2020 (Figure 1).^23^ All four peptides share a central scaffold between residues two and eight but vary at their lipidated N-terminal amino acid and C-terminal macrocycle. Although these peptides were only recently discovered, the naming convention has already become somewhat complicated. We therefore propose “*ornicidines*” as a family name for these peptides, given that all contain a conserved central scaffold bearing three ornithine residues.

We recently reported synthetic methods to access brevicidine and laterocidine, as well as novel cyclic analogues.^24^ Subsequent SAR studies revealed that the natural branched and/or chiral N-terminal acyl chains can be substituted with more affordable linear alternatives containing between eight and twelve carbons.^25^ The costliest part in the syntheses of these peptides arises from the need to cyclize the side chain of Thr9 with the C-terminus. This also presents a challenge for synthesis by automated Fmoc solid-phase peptide synthesis (SPPS), as on-resin cyclization requires low resin loading, the use of orthogonal protection strategies, and extended reaction times for the cyclization step. Given that the macrocyclic rings found in the ornicidines differ both in size and amino acid composition, we were inspired to investigate the necessity of the macrocycle motif. In their seminal report on brevicidine and laterocidine, Li et al. found that a linear analogue of laterocidine was 8- to 32-fold less active than the natural cyclic peptide.^22^ However, only one linear analogue was synthesized and tested in their study. To truly ascertain if the macrocyclic ring is essential for antibacterial activity, we embarked on the linearization of these peptides, the details of which are presented herein.

## Results and Discussion

Linear laterocidine (LL-OH, **4**) and linear brevicidine (LB-OH, **5**) were first synthesized by Fmoc SPPS, starting from 2-chlorotrityl (2-CT) chloride resin. The antibacterial activity of these peptides was then compared to brevicidine (**1**) and laterocidine (**2**) using minimum inhibitory concentration (MIC)/microbroth dilution assays (Table 1). The MICs of all peptides were determined against a panel of Gram-negative and Gram-positive ESKAPE pathogens. Additionally, a strain of *E. coli* carrying the *mcr*-1 gene was included in the panel. The *mcr*-1 gene is a transferrable plasmid that confers resistance against colistin, a last-resort antibiotic for Gram-negative infections, by reducing the overall negative charge of lipopolysaccharide (LPS) through modification of lipid A with phosphoethanolamine. The propensity for cationic antibiotics to bind to the cell membrane is therefore reduced. This gene has been observed in more than 27 bacterial species over six continents.^26^ The spread of the *mcr*-1 gene could render colistin ineffectual; therefore, novel antibacterials are urgently required to overcome this form of resistance.

**Table 1:** Activity of Linear Brevicidine and Laterocidine C-Terminal Analogue

	Antibacterial activity (μg/mL)						% Hemolysis
Compound	*E. coli* ATCC 25922	*E. coli* (MCR-1)	*K. pneumoniae* ATCC 13883	*A. baumannii* ATCC 17961	*P. aeruginosa* PAO1	*S. aureus* USA300	Sheep red blood cells
Brev (**1**)	4	4	2	4	8	>32	<0.1
Lat (**2**)	2	2	2–4	2	4	>32	1.3
LL-OH (**4**)	8	16	8–16	16	8	>32	<0.1
LB-OH (**5**)	>32	>32	>32	>32	16	>32	<0.1
LL-NH_2_ (**6**)	4	8	4–8	8	4	>32	<0.1
LB-NH_2_ (**7**)	16	16	16	16	4	>32	<0.1
Δ13LL-NH_2_ (**8**)	8	8	4–8	4	4	>32	<0.1
Δ12LB-NH_2_ (**9**)	8	16	8	8	4	>32	3.8
Δ12–13LL-NH_2_ (**10**)	4	8	8–16	4	4	>32	3.0
Δ11–12LB-NH_2_ (**11**)	4	4–8	2	8	2	>32	<0.1
Δ11–13LL-NH_2_ (**12**)	4	4	2	4	4	32	2.3
Δ10–12LB-NH_2_ (**13**)	>32	>32	>32	>32	>32	>32	<0.1
Δ10–13LL-NH_2_ (**14**)	>32	32	>32	32	>32	>32	<0.1
Δ9–12LB-NH_2_ (**15**)	>32	>32	>32	>32	>32	>32	<0.1
Δ9–13LL-NH_2_ (**16**)	>32	>32	>32	32	>32	>32	<0.1

In line with expectation, both LL-OH (**4**) and LB-OH (**5**) were found to be significantly less active than their cyclic counterparts. However, due to the presence of C-terminal carboxylic acids in both peptides, their net charge (2+) is lower than brevicidine/laterocidine (3+). The overall cationic charge of an antibacterial peptide is a critical property for preferential binding to the negatively charged phospholipids found throughout the bacterial membrane.^27^ We therefore postulated that by masking the C-terminus with an amide, antibacterial activity could be improved. To this end, the linear C-terminal amide analogues of laterocidine (LL-NH_2_, **6**) and brevicidine (LB-NH_2_, **7**) were synthesized by Fmoc SPPS from rink amide resin and tested for antibacterial activity. Gratifyingly, both **6** and **7** showed a 2-fold improvement in activity with respect to C-terminal acids **4** and **5**, overall a 2/4-fold reduction compared to parent peptides.

Having demonstrated that the C-terminal macrocycle is not essential for antibacterial activity, we set out to determine whether the C-terminus of LB-NH_2_ or LL-NH_2_ could be truncated. Shorter peptides require less reagents and synthesis time and are therefore cheaper to synthesize. Amide analogues **8**–**16** were synthesized, whereby the C-terminal residues were removed sequentially up to and including Thr9. This revealed that for LB-NH_2_ both Ser12 and Gly11 can be removed without diminishing antibacterial activity.

However, further removal of the third and fourth C-terminal residues as in analogues **13** and **15**, respectively, resulted in substantial decreases in activity. Truncated LL-NH_2_ analogues showed a similar trend, with removal of the three C-terminal residues (Asn11, Gly12, and Gly13) having no effect on the MIC values. Ile10 was equally implicated as a key residue. At this stage, it must be emphasized that these linear analogues are substantially easier and cheaper to synthesize than brevicidine or laterocidine but retain full activity. Albeit, it is unclear whether these linear peptides operate via the same mechanism of action (MOA) as the parent peptides. Only minimal hemolysis (Table 1) was detected for these peptides at concentrations up to 32× higher than the MIC of the most potent peptides, showing that linearization does not increase hemolytic activity and suggesting that a similar MOA is retained.

**Figure 2:**
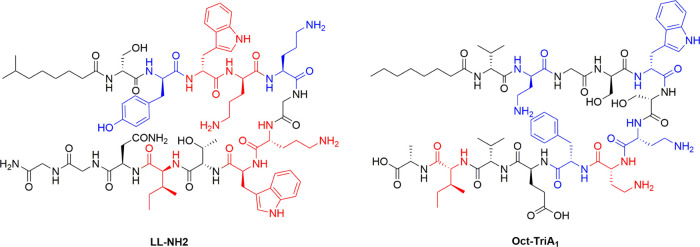
Comparison of essential residues in linear laterocidamide and Oct-TriA_1_.^15^ Blue residues result in a 4- to 8-fold loss in activity against *E. coli* ATCC 25922 upon substitution with alanine, and red residues result in a 16-fold or greater loss.

Given the ease with which these linear analogues could be prepared, we next proceeded to perform an alanine scan on both LB-NH_2_ (analogues **17**–**28**) and LL-NH_2_ (analogues **29**–**41**) (Table 2). Consistent with our truncation studies, substitution of Asn11, Gly12, or Gly13 in LL-NH_2_, or Ser12 in LB-NH_2_, had minimal effect on activity, giving analogues with MIC values of 4–8 μg/mL. Interestingly, the replacement of Gly11 in LB-NH2 was significantly detrimental to activity, showing that although both laterocidine and brevicidine have very similar peptide sequences, changes that work on one are not guaranteed to work on the other. Furthermore, for both the LB-NH_2_ and LL-NH_2_ peptides, the N-terminal residues (d-Tyr2, d-Trp3, d-Orn4, Orn5, d-Orn7, and Trp8) were found to be integral to antibacterial activity (Figure 2). These residues are either aromatic or cationic. The former may be important for maintaining an active conformation through π-stacking or increasing the hydrophobicity of the peptide to aid passage through the bacterial membrane,^28^ while the latter may play a role in target binding through electrostatic interactions, in addition to binding phospholipids present in the membrane.^29^

**Table 2:** Alanine Scan of Linear Brevicidamide and Laterocidamide

	Antibacterial activity (μg/mL)					
Compound	*E. coli* ATCC 25922	*E. coli* (MCR-1)	*K. pneumoniae* ATCC 13883	*A. baumannii* ATCC 17961	*P. aeruginosa* PAO1	*S. aureus* USA300
LB(d-Ala1)-NH_2_ (**17**)	16	16	16	8	8	>32
LB(d-Ala2)-NH_2_ (**18**)	>32	>32	>32	>32	16	>32
LB(d-Ala3)-NH_2_ (**19**)	>32	>32	>32	>32	32	>32
LB(d-Ala4)-NH_2_ (**20**)	>32	>32	>32	32	32	>32
LB(Ala5)-NH_2_ (**21**)	>32	>32	>32	>32	32	>32
LB(Ala6)-NH_2_ (**22**)	32	32	>32	32	32	>32
LB(d-Ala7)-NH_2_ (**23**)	>32	>32	>32	16	32	>32
LB(Ala8)-NH_2_ (**24**)	>32	>32	>32	>32	32	>32
LB(Ala9)-NH_2_ (**25**)	8	16	16	16	4	>32
LB(Ala10)-NH_2_ (**26**)	>32	>32	>32	>32	16	>32
LB(Ala11)-NH_2_ (**27**)	32	32	>32	32	16	>32
LB(Ala12)-NH_2_ (**28**)	8	8	8	4	8	>32
LL(d-Ala1)-NH_2_ (**29**)	8	8	>32	32	8	>32
LL(d-Ala2)-NH_2_ (**30**)	32	32	>32	>32	16	>32
LL(d-Ala3)-NH_2_ (**31**)	>32	>32	>32	>32	>32	>32
LL(d-Ala4)-NH_2_ (**32**)	>32	>32	>32	32	>32	>32
LL(Ala5)-NH_2_ (**33**)	32	>32	>32	>32	32	>32
LL(Ala6)-NH_2_ (**34**)	4–8	8	8	16	16	>32
LL(d-Ala7)-NH_2_ (**35**)	>32	>32	>32	32	32	>32
LL(Ala8)-NH_2_ (**36**)	>32	>32	>32	>32	32	>32
LL(Ala9)-NH_2_ (**37**)	4	4–8	4	8	4	>32
LL(Ala10)-NH_2_ (**38**)	>32	>32	>32	>32	16	>32
LL(Ala11)-NH_2_ (**39**)	8	8	8	8	4	>32
LL(Ala12)-NH_2_ (**40**)	8	8	8	8	8	>32
LL(Ala13)-NH_2_ (**41**)	4–8	8	8	8	4	>32

The results of the alanine scans are notably like those observed with the tridecaptins, a class of linear antibacterial NRPs. In TriA_1_, three cationic residues (d- and l-Dab) and two aromatic amino acids are also essential for antibacterial activity (Figure 2).^15^ TriA_1_ kills Gram-negative bacteria by binding to LPS on the outer membrane, entering the periplasm, selectively binding to Gram-negative lipid II, and disrupting the proton motive force (PMF).^30^ Brevicidine, laterocidine, and the relacidines also bind to LPS,^21,22^ although the relacidines were reported not to bind lipid II,^22^ and brevicidine interacts with phosphatidylglycerol and cardiolipin on the inner membrane to disrupt the PMF.^31^ Our studies suggest that the central scaffold of these peptides represents a key epitope responsible for LPS binding and/or membrane disruption.

Another particularly interesting observation is that Thr9, whose side chain cyclizes with the C-terminus in natural ornicidines, is not essential for activity. This led us to consider amino acid substitutions at this position that may enhance the antibacterial activity of the linear peptides. To study this, a library of position 9-modified LL-NH_2_ analogues (**42**–**52**), and ΔSer12-LB-NH_2_ analogues (**53**–**63**) were synthesized and assessed for antibacterial activity (Table 3).

**Table 3:** Position 9 Modification of Peptides

	Antibacterial activity (μg/mL)						% Hemolysis
Compound	*E. coli* ATCC 25922	*E. coli* (MCR-1)	*K. pneumoniae* ATCC 13883	*A. baumannii* ATCC 17961	*P. aeruginosa* PAO1	*S. aureus* USA300	Sheep red blood cells
LL(Leu9)-NH_2_ (**42**)	4	8	8	4	8	>32	<0.1
LL(Phe9)-NH_2_ (**43**)	4	4	8	4	8	>32	0.6
LL(Met9)-NH_2_ (**44**)	4	4	4	4	4	>32	0.6
LL(Trp9)-NH_2_ (**45**)	4	4	4	4	16	>32	2.1
LL(Ser9)-NH_2_ (**46**)	4	8	4	8	4	>32	<0.1
LL(Asn9)-NH_2_ (**47**)	8	8	8–16	16	8	>32	<0.1
LL(Gln9)-NH_2_ (**48**)	4	8	4	8	4	>32	0.6
LL(MeAbu9)-NH_2_ (**49**)	4	4	4	2	4	>32	<0.1
LL(Dap[Alloc]9)-NH_2_ (**50**)	2	4	4	4	8	>32	<0.1
LL(Glu[OAll]9)-NH_2_ (**51**)	16	16	>32	16	>32	>32	<0.1
LL(Dap9)-NH_2_ (**52**)	8	8	32	16	18	>32	<0.1
Δ12LB (Leu9)-NH_2_ (**53**)	4	4	4	4	8	>32	1.1
Δ12LB (Phe9)-NH_2_ (**54**)	2	2–4	2–4	4	4	>32	1.1
Δ12LB (Met9)-NH_2_ (**55**)	4	4	4	4	4	>32	0.7
Δ12LB (Trp9)-NH_2_ (**56**)	4	4	8	4	8	16	0.8
Δ12LB (Ser9)-NH_2_ (**57**)	4	8	4	8	2	>32	0.6
Δ12LB (Asn9)-NH_2_ (**58**)	8	8	16	16	2	>32	0.2
Δ12LB (Gln9)-NH_2_ (**59**)	8	8	16	16	2	>32	0.5
Δ12LB (MeAbu9)-NH_2_ (**60**)	4	4	2	2	4	>32	0.7
Δ12LB (Dap[Alloc]9)-NH_2_ (**61**)	4	4	2	2	4	>32	<0.1
Δ12LB (Glu[OAll]9)-NH_2_ (**62**)	4	8	4	8	2	>32	1.1
Δ12LB (Asp9)-NH_2_ (**63**)	>32	>32	>32	>32	4	>32	0.3
Δ12LB (Dap9)-NH_2_ (**64**)	8	8	>32	16	4	>32	0.3

ΔSer12-LB-NH_2_, rather than LB-NH_2_ was selected as the scaffold so that the effect of the well-tolerated truncation and position 9 modification could be studied concurrently and compared to full-length LL-NH_2_. In both peptides, position 9 modifications were surprisingly well tolerated, with hydrophobic residues (Leu, Phe, Met, and Trp) and polar residues (Ser, Asn, Gln, MeAbu, Dap(Alloc), and Glu(OAll)) having little to no effect on antibacterial activity. Inclusion of an anionic acid at this position (Asp) abolished activity against most strains (excluding *P. aeruginosa*), showing the importance of having a net +3 charge on the peptide. However, addition of an additional cationic residue (Dap) did not increase antibacterial activity. At this stage, with 60 novel linear peptides assessed, our most active analogues were ΔSer12-LB(Phe9)-NH_2_ (**54**), and LL(Dap(Alloc)9)-NH_2_ (**61**), both showing MICs of 2–4 μg/mL against most strains, including MCR-1 producing *E. coli*. As for the initial series of linear analogues (Table 1), we also investigated whether the structural alterations to brevicidine and laterocidine in peptides **42**–**64** would impart an antibacterial MOA based on a general detergent effect. Such a nonspecific MOA is often identified by an increase in hemolytic activity compared to the natural peptides. To this end, all novel LB-NH_2_ and LL-NH_2_ analogues prepared were incubated with sheep erythrocytes for 1 h at 37 °C and the hemolysis was assessed. Gratifyingly, all peptides displayed low hemolytic activity (<5%) when tested at 32× MIC with only slight hemolysis observed for Δ12LB-NH_2_ (**9**) and Δ12–13LL-NH_2_ (**10**) measured at 3.8 and 3.0%, respectively (Table 3). The low levels of hemolysis observed suggest a specific MOA is at play. However, future studies will be required to elucidate the exact mechanism by which these peptides operate.

## Conclusions

Cyclic NRPs are a common class of antibacterial agents encountered in Nature. Through the work described above, we have shown that the C-terminal macrocycles in brevicidine (**1**) and laterocidine (**2**) are not required for antibacterial activity. Although linear peptides bearing C-terminal carboxylates were significantly less active than their cyclic counterparts, amidation of the C-terminus increased the activity of the linear species to levels comparable to the natural products without introducing hemolytic effects. Further modification at Thr9, whose side chain forms the macrocycle with the C-terminus, yielded novel linear analogues that exhibit potent anti-Gram-negative activity, including against MCR-1 producing *E. coli*. *P. aeruoginosa* is a major contributor to nosocomial infection rates, in fact the World Health Organisation (WHO) has listed carbapenem-resistant *P. aeruginosa* as one of the top three critical priority pathogens that require antibiotic research.^32^ Gratifyingly, the peptides presented herein also demonstrate equipotent or improved activity against *P. aeruginosa*. These linear peptides are more economical to synthesize as they do not require additional orthogonal protecting groups or extra cyclization steps. The convenience offered by the linear C-terminal amide brevicidine and laterocidine analogues also enabled an alanine scan study. This revealed that for both peptides, the residues (d-Tyr2, d-Trp3, d-Orn4, Orn5, d-Orn7, and Trp8) are essential for bioactivity. These residues are either cationic or aromatic (hydrophobic), which are key for target binding and maintaining an active conformation to cross the bacterial cell membrane. Of course, further MOA studies will be required to confirm how these peptides elicit their activity. The results of such assays will likely complement the findings of our alanine scan. Additionally, linear peptides are often more prone to proteolytic degradation than their cyclic counterparts. However, the large number of d-amino acids present in these novel linear analogues likely imparts excellent protection against proteolytic degradation, but further studies will be required to confirm this.

In nature, cyclic NRPs are prepared by non-ribosomal peptide synthetases, with the cyclization step performed by the thioesterase domain. To the best of our knowledge, this is the only natural method by which microorganisms can mask the C-terminus of NRPs. To date, the linearization of NRPs has not been frequently reported, raising the intriguing possibility that the “linearization + amidation strategy” here reported might also be used in preparing functional linear analogues of other cyclic NRPs. Due to their constrained structures, cyclic peptides are intrinsically more ordered and suffer a lower entropic penalty upon target binding than linear peptides. However, many NRAPs are amphiphilic in nature and adopt ordered secondary structures upon interacting with the bacterial membrane. This may help offset the entropic penalty for some linear NRAPs (e.g., TriA_1_^33^) and account for the retention of activity observed with the linear ornicidine analogues reported in this study. Efforts to further establish the general applicability of this approach with cyclic NRPs that possess promising biological activities, and to assess the mechanism of action of the reported linear ornicidine analogues, are now underway and will be reported in due course.

## Experimental Section

### General

All proteinogenic Fmoc-amino acids used in this study were purchased from CEM or P3 BioSystems. For brevicidine analogues, Fmoc-d-Asn(Trt)-OH, Fmoc-d-Tyr(tBu)-OH, Fmoc-d-Trp(Boc)-OH, Fmoc-l-Glu(OAll)-OH, diisopropylethylamine (DIPEA), 2-(7-Aza-1*H*-benzotriazole-1-yl)-1,1,3,3-tetramethyluronium hexafluorophosphate (HATU), triisopropylsilane (TIPS), 4-methylpiperidine, phenylsilane, trifluoroacetic acid (TFA), and tetrakis(triphenylphosphine)palladium were purchased from Fluorochem. Fmoc-l-Orn(Boc)-OH, Fmoc-d-Orn(Boc)-OH, Fmoc-l-Dap(Alloc)-OH, and (2*S*,3*R*)-(Fmoc-amino)-3-azidobutyric acid were purchased from ChemImpex. 4-Methylhexanoic acid and 2-chlorotrityl chloride (2-CT) resin (200–400 mesh) were purchased from Sigma-Aldrich. Rink amide MBHA resin (100–200 mesh), diethyl ether, HPLC grade acetonitrile (ACN), dichloromethane (DCM), and *N*,*N*-dimethylformamide (DMF) were purchased from Sigma-Aldrich. For laterocidine analogues, Fmoc-l-Orn(Boc)-OH, Fmoc-d-Orn(Boc)-OH, Fmoc-l-Dap(Alloc)-OH, and Fmoc-l-Glu(OAll)-OH were purchased from Combi-Blocks. 2-CT resin and Rink Amide MBHA were purchased from P3 BioSystems and Iris Biotech, respectively. Isopelargonic acid was purchased from Enamine. ((1*H*-Benzo[*d*][1,2,3]triazol-1-yl)oxy)tris(dimethylamino) phosphonium hexafluorophosphate (BOP), *N*,*N*-diisopropylcarbodiimide (DIC), ethyl cyanohydroxyiminoacetate (Oxyma), and TIPS were purchased from Manchester Organics. DIPEA, piperidine, TFA, and dimethylsulfoxide (DMSO) were purchased from Carl Roth. DCM and petroleum ether were purchased from VWR Chemicals. ACN, DMF, and methyl tertiary-butyl ether (MTBE) were purchased from Biosolve. All chemicals were used without further purification. All synthetic compounds are >95% pure by HPLC analysis.

### Peptide Synthesis

Peptides **1** and **2** were synthesized and purified as previously described.^22,23^ Brevicidine analogues with a C-terminal acid were synthesized manually on a 0.05 mmol scale on 2-CT resin preloaded with Fmoc-Gly-OH in a Merrifield vessel. The resin loading was determined to be 0.73 mmol/g. The resin was initially swollen by bubbling in DMF (3 mL) for 10 min. The solvent was discharged and the Fmoc group was removed by the addition of a 20% solution of 4-methylpiperidine in DMF (3 × 3 mL, 2 × 1 min then 1 × 3 min). The resin was washed with DMF (3 × 3 mL), and a coupling solution of amino acid (6 equiv), HATU (6 equiv), and DIPEA (12 equiv) in DMF (3 mL) was added. The coupling was bubbled with argon for 1 h before being discharged, and the resin was washed with DMF (3 × 3 mL). The cycle of deprotections and couplings was repeated to obtain the full lipopeptide. Brevicidine analogues with a C-terminal amide were synthesized using a Liberty Blue HT12 system. Automated SPPS was performed on a 0.05 mmol scale using Fmoc chemistry on Rink amide resin. Factory settings were used for all coupling and deprotection cycles. Asymmetrically protected amino acids were used as 0.2 M solutions in DMF, with amino acid subunits being coupled using HATU as the activator and DIPEA as the activator base and heated to 70 °C for 3 min. Upon completion of synthesis, the peptide resin was washed with DCM (3 × 5 mL) and dried under a positive pressure of argon for 15 min. Global deprotection and resin cleavage was performed using a cocktail of TFA/TIPS/H_2_O (5 mL, 95:2.5:2.5) at 37 °C for 1 h with frequent agitation. The cleavage solution was filtered through a glass wool plug and concentrated in vacuo. Cold diethyl ether was added to crash out the peptide, the suspension was centrifuged (3500 rpm, 3 min), and the solvent was decanted. The pellet was resuspended in fresh diethyl ether and centrifuged (3500 rpm, 3 min) again. The solvent was decanted, and the crude pellet was dissolved in a minimal amount of 20% acetonitrile solution in water with 0.1% TFA. The peptides were subsequently purified by reversed-phase high-performance liquid chromatography (RP-HPLC) (**Method A**). Laterocidine analogues containing a C-terminal acid were synthesized manually on a 0.1 mmol scale on 2-CT resin preloaded with Fmoc-Gly-OH. The resin loading was determined to be 0.73 mmol g^–1^. All couplings with the exception of the lipid were performed using amino acid (4 eq.), BOP (4 equiv), and DIPEA (8 equiv) in DMF (5 mL) for 1 h at room temperature under a positive pressure of nitrogen. The lipid was coupled by treating the resin with isopelargonic acid (2 equiv), BOP (2 equiv), and DIPEA (4 equiv) in DMF (5 mL) overnight at room temperature. Fmoc removal was performed by treating the resin with 20% piperidine solution in DMF (5 mL, 1 × 5 min then 1 × 15 min). Laterocidine analogues with a C-terminal amide were synthesized automatically using a CEM Liberty Blue automated peptide synthesizer with microwave irradiation. Couplings were performed at 0.125 M concentration using amino acid (5 equiv), DIC (5 equiv), and Oxyma (5 equiv). Fmoc removal was performed using piperidine/DMF (1:4, v/v). Final side chain deprotection and cleavage from resin was carried out by treating the resin with a cocktail of TFA/TIPS/H_2_O (5 mL, 95:2.5:2.5, v/v) for 90 min. The reaction mixture was filtered through cotton, and the filtrate was precipitated with MTBE/petroleum ether (1:1, v/v) and centrifuged (4500 rpm, 5 min). The pellet was resuspended in MTBE/petroleum ether (1:1, v/v) and centrifuged again (4500 rpm, 5 min). The crude pellet was dissolved in tBuOH/H_2_O (1:1, v/v) and lyophilized overnight. The crude mixtures were subsequently purified by RP-HPLC (**Method B**).

### Purification and Analysis of Peptides

Brevicidine analogues were purified by reversed-phase high-performance liquid chromatography (RP-HPLC). Purification was performed on a PerkinElmer HPLC system composed of a 200 series binary pump, UV/vis detector, vacuum degasser, Rheodyne 7725i injector. The system was operated using Thermo Fisher Chromeleon 7.2 software. **Method A**: Phenomenex Luna C18 column (5 μg, 250 mm × 21.2 mm) equipped with a 2 mL sample loop. Runs were performed at a flow rate of 10 mL/min with UV detection at 220 nm. Solvent *A* = 0.1% TFA in Milli-Q water and solvent *B* = 0.1% TFA in ACN. A gradient method was employed, starting from 5% *B* and 95% *A* for 5 min, ramping up to 8% *B* over 20 min, then ramping up to 20% *B* over 15 min, ramping up to 30% *B* over 3 min, ramping again up to 95% *B* over 4 min, remaining at 95% *B* for 3 min, ramping down to 5% *B* over 2 min before staying at 5% *B* for 5 min. **Method B**: Purification was performed on a BESTATechnik system with a Dr. Maisch ReproSil Gold 120 C18 column (10 μm, 25 × 250 mm^2^) and equipped with an ECOM Flash UV detector. Runs were performed at a flow rate of 12 mL/min with UV detection at 214 and 254 nm. Solvent *A* = 0.1% TFA in water/ACN (95:5) and solvent *B* = 0.1% TFA in water/ACN (5:95). A gradient method was employed, starting at 100% solvent *A* for 5 min, ramping up to 70% solvent *B* over 50 min, remaining at 70% solvent *B* for 3 min before ramping down to 100% solvent *A* over 1 min and remaining there for 5 min. Product-containing fractions were pooled, partially concentrated under vacuum, frozen, and then lyophilized to yield pure peptides as white flocculent solids. A small amount of purified peptide was analyzed by analytical HPLC. Peptide purity was quantified by analytical HPLC (Brevicidine analogues—**Method C**, laterocidine analogues—**Method D**). **Method C (Analytical)**: A Phenomenex Luna C18 column (5 μm, 150 mm × 4.6 mm) was used, with samples were injected to a 200 μL sample loop. The flow rate was set at 2 mL/min, and UV/vis absorbance was measured at 220 nm. Gradient elution was again employed using the same solvents, *A* and *B*. It began at 20% *B* and 80% *A* for 2 min, before ramping to 95% *B* over 18 min. *B* was then decreased to 20% over 0.1 min and held for 3.9 min. **Method D (Analytical)**: Analytical runs of laterocidine analogues were performed on a Shimadzu Prominence-i LC-2030 system with a Dr. Maisch ReproSil Gold 120 C18 (5 μm, 4.6 mm × 250 mm) at 30 °C. Runs were performed at a flow rate of 1 mL/min with UV detection at 214 and 254 nm. Solvent *A* = 0.1% TFA in water/ACN (95:5) and solvent *B* = 0.1% TFA in water/ACN (5:95). A gradient method was employed, starting at 100% solvent *A* for 2 min, ramping up to 50% solvent *B* over 45 min, ramping up to 100% solvent *B* over 1 min, remaining at 100% solvent *B* for 6 min before ramping down to 100% solvent *A* over 1 min and remaining there for 5 min. Electrospray ionization high-resolution mass spectrometry (ESI-HRMS) was carried out on all purified brevicidine analogues by the analytical services and environmental projects (ASEP) Department at Queen’s University Belfast. A Waters LCT Premier ToF mass spectrometer was used to obtain the relevant spectra. HRMS spectra of laterocidine peptides were performed on a Thermo Scientific Dionex UltiMate 3000 HPLC system with a Phenomenex Kinetex C18 (2.6 μm, 2.1 mm × 150 mm) column at 35 °C and equipped with a diode array detector. The following solvent system, at a flow rate of 0.3 mL/min, was used: solvent *A* = 0.1% formic acid in water, solvent *B* = 0.1% formic acid in acetonitrile. A gradient method was employed, starting at 95% solvent *A* and 5% solvent *B* for 1 min, ramping up to 95% solvent *B* over 9 min, ramping up to 98% solvent *B* over 1 min, remaining there for 1 min before ramping back down to 95% solvent *A* over 2 min and remaining there for 1 min. The system was connected to a Bruker micrOTOF-Q II mass spectrometer (ESI ionization) calibrated internally with sodium formate.

### Antibacterial Testing

All minimum inhibitory concentrations (MICs) were determined according to Clinical and Laboratory Standards Institute (CLSI) guidelines. Blood agar plates were inoculated with glycerol stocks of *E. coli* ATCC 25922, *K. pneumoniae* ATCC 13883, *A. baumannii* ATCC 17961, *P. aeruginosa* PAO1, and *S. aureus* USA300. The inoculated agar plates were then incubated for 16 h at 37 °C. Individually grown colonies were subsequently used to inoculate 5 mL aliquots of TSB that were then incubated at 37 °C with shaking at 220 rpm. *E. coli* 25922 MCR-1 glycerol stock was used to inoculate 5 mL of TSB supplemented with kanamycin that was then incubated for 16 h at 37 °C with shaking at 220 rpm. The next day the culture was diluted 100-fold in TSB supplemented with kanamycin and incubated at 37 °C with shaking at 220 rpm. In parallel, the lipopeptide antibiotics DMSO stocks to be assessed were serially diluted with MHB in polypropylene 96-well plates (50 μL in each well). Colistin sulfate stocks were dissolved in water before being diluted with MHB. Aliquots of the inoculated TSB were incubated until an OD_600_ of around 0.5 was reached. The bacterial suspensions were then diluted with MHB (2 × 10^5^ CFU/mL) and added to the microplates containing the test compounds (50 μL to each well). The well plates were sealed with an adhesive membrane and after 18 h of incubation at 37 °C with shaking at 600 rpm, the wells were visually inspected for bacterial growth. MIC values reported are based on three technical replicates and defined as the lowest concentration of the compound that prevented visible growth of bacteria.

### Hemolytic Assays

Experiments were performed in triplicate, and Triton X-100 was used as a positive control. Red blood cells from defibrinated sheep blood obtained from Thermo Fisher were centrifuged (400 g for 15 min at 4 °C) and washed with phosphate-buffered saline (PBS) containing 0.002% Tween20 (buffer) five times. Then, the red blood cells were normalized to obtain a positive control read-out between 2.5 and 3.0 at 415 nm to stay within the linear range with the maximum sensitivity. A serial dilution of the compounds (128–1 μg/mL, 75 μL) was prepared in a 96-well polypropylene plate. The outer border of the plate was filled with 75 μL of buffer. Each plate contained a positive control (0.1% Triton-X final concentration, 75 μL) and a negative control (buffer, 75 μL) in triplicate. The normalized blood cells (75 μL) were added, and the plates were incubated at 37 °C for 1 h while shaking at 500 rpm. A flat-bottom polystyrene plate with 100 μL buffer in each well was prepared. After incubation, the plates were centrifuged (800 g for 5 min at room temperature) and 25 μL of the supernatant was transferred to their respective wells in the flat-bottom plate. The values obtained from a read-out at 415 nm were corrected for background (negative control) and transformed to a percentage relative to the positive control.

## Supporting Information Available

Additional hemolysis data, and analytical data including HRMS values, and analytical HPLC traces (PDF)
Molecular formula strings (CSV)

(PDF)
(CSV)

## Abbreviations Used

ACN: acetonitrile
AMR: antibacterial resistance
BOP: ((1*H*-benzo[*d*][1,2,3]triazol-1-yl)oxy)tris(dimethylamino) phosphonium hexafluorophosphate
CFU: colony-forming units
CLSI: Clinical and Laboratory Standards Institute
2-CT: 2-chlorotrityl
Dap: diaminopropionic acid
DCM: dichloromethane
DIC: diisopropylcarbodiimide
DIPEA: diisopropylethylamine
DMF: dimethylformamide
DMSO: dimethylsulfoxide
ESI: electrospray ionization
Fmoc: fluorenylmethyloxycarbonyl
HATU: *O*-(7-azabenzotriazol-1-yl)-*N*,*N*,*N*′,*N*′-tetramethyluronium hexafluorophosphate
HPLC: high-performance liquid chromatography
HRMS: high-resolution mass spectrometry
LB: linear brevicidine
LL: linear laterocidine
LPS: lipopolysaccharide
MHB: Mueller Hinton broth
MIC: minimum inhibitory concentration
MTBE: methyl tertiary-butyl ether
NRP: non-ribosomal peptide
Oct: octanoyl
Oxyma: ethyl cyanohydroxyiminoacetate
PBS: phosphate-buffered saline
PMF: proton motive force
RP-HPLC: reversed-phase high-performance liquid chromatography
SAR: structure–activity relationship
SPPS: solid-phase peptide synthesis
TFA: trifluoroacetic acid
TIPS: triisopropylsilane
Tri: tridecaptin
TSP: tryptic soy broth
