# Supplementary material for: Linearization of the Brevicidine and Laterocidine Lipopeptides Yields Analogues That Retain Full Antibacterial Activity
Source: J Med Chem. 2023 Apr 18;66(8):6002–9. doi: 10.1021/acs.jmedchem.3c00308 (PMC10150354; doi:10.1021/acs.jmedchem.3c00308)
Supplement: Supplementary file 1 — jm3c00308_si_001.pdf [file jm3c00308_si_001.pdf]

Supplementary Information

FOR

Linearization of the Brevicidine and Laterocidine Lipopeptides Yields Analogues that Retain Full Antibacterial Activity

Ross D. Ballantine,[‡a] Karol Al Ayed,[‡b] Samantha J. Bann,[a] Michael Hoekstra,[b] Nathaniel I. Martin*[b] and Stephen A. Cochrane*[a]

[a] School of Chemistry and Chemical Engineering, David Keir Building, Stranmillis Road, Queen's University Belfast, Belfast, UK, BT9 5AG; [b] Biological Chemistry Group, Institute of Biology, Leiden University, Sylviusweg 72, 2333 BE, Leiden, The Netherlands.

‡Equal contribution
*To whom correspondence should be addressed.

Email:s.cochrane@qub.ac.uk and n.i.martin@biology.leidenuniv.nl

TABLE OF CONTENTS

Section	Contents	Page Number
I	Hemolytic assays	S2
II	HPLC and HRMS analysis of peptides	S3

I. Hemolytic assays

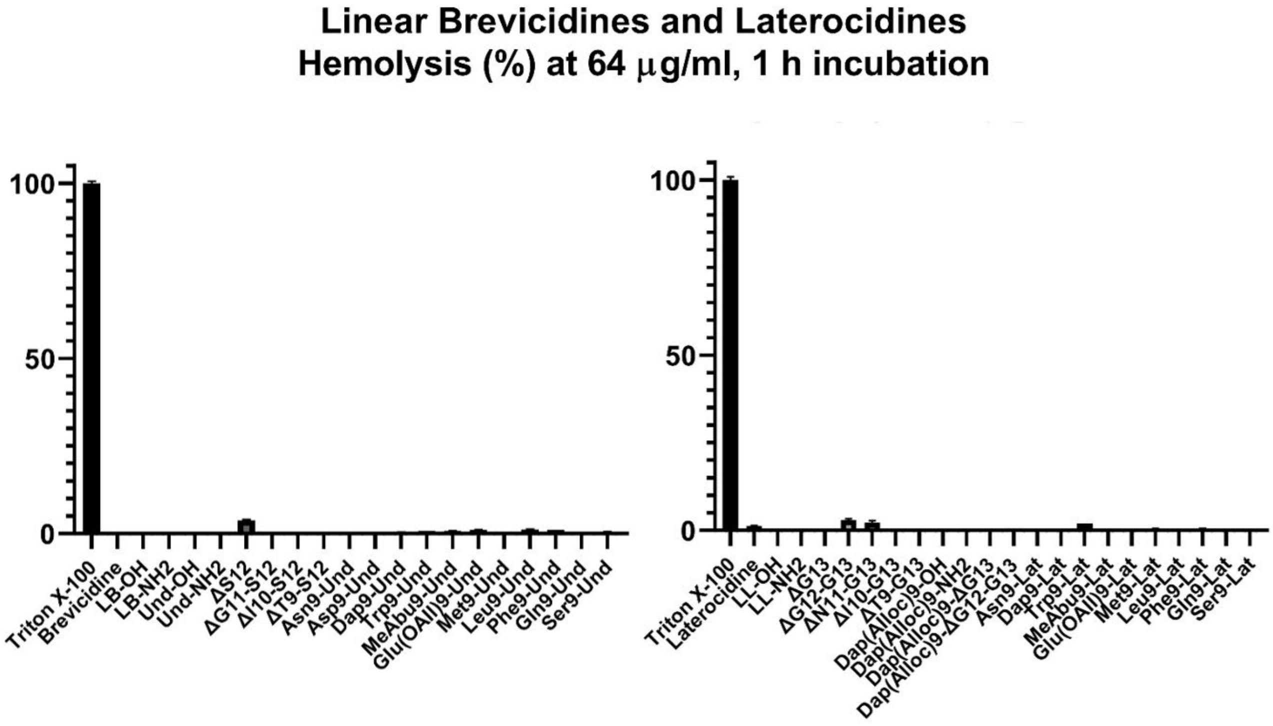

Fig. S1. Hemolytic assays of ornicidine analogues at 64 μg mL^{-1} following 1 hour incubation with sheep blood cells

Experiments were performed in triplicate and Triton X-100 used as a positive control. Red blood cells from defibrinated sheep blood obtained from Thermo Fisher were centrifuged (400 g for 15 min at 4°C) and washed with Phosphate-Buffered Saline (PBS) containing 0.002% Tween20 (buffer) five times. Then, the red blood cells were normalized to obtain a positive control read-out between 2.5 and 3.0 at 415 nm to stay within the linear range with the maximum sensitivity. A serial dilution of the compounds (128 – 1 μg/mL, 75 μL) was prepared in a 96-well polypropylene plate. The outer border of the plate was filled with 75 μL buffer. Each plate contained a positive control (0.1% Triton-X final concentration, 75 μL) and a negative control (buffer, 75 μL) in triplicate. The normalized blood cells (75 μL) were added and the plates were incubated at 37 °C for 1 h while shaking at 500 rpm. A flat-bottom polystyrene plate with 100 μL buffer in each well was prepared. After incubation, the plates were centrifuged (800 g for 5 min at room temperature) and 25 μL of the supernatant was transferred to their respective wells in the flat-bottom plate. The values obtained from a read-out at 415 nm were corrected for background (negative control) and transformed to a percentage relative to the positive control.

II. HPLC and HRMS analysis of peptides

Table S1. Peptide number, name, chemical formula, exact mass, mass found and overall yield for peptides **4** – **64**.

Peptide	Name	Chemical Formula	Calcd Exact Mass	Mass found	Calcd	Overall Yield [%]
**4**	LL-OH	$C_{78}H_{115}N_{19}O_{19}$	1621.8617	811.9386 $[M+2H]^{2+}$	811.9381	19
**5**	LB-OH	$C_{74}H_{108}N_{18}O_{18}$	1536.8089	769.4144 $[M+2H]^{2+}$	769.4117	8
**6**	LL-NH_2	$C_{78}H_{116}N_{20}O_{18}$	1620.8776	811.4464 $[M+2H]^{2+}$	811.4461	29
**7**	LB-NH_2	$C_{74}H_{109}N_{19}O_{17}$	1535.8249	768.9221 $[M+2H]^{2+}$	768.9200	8
**8**	Δ13LL-NH_2	$C_{76}H_{113}N_{19}O_{17}$	1563.8562	782.9358 $[M+2H]^{2+}$	782.9354	29
**9**	Δ12LB-NH_2	$C_{71}H_{104}N_{18}O_{15}$	1448.7929	1471.7715 $[M+H]^{+}$	1471.7821	38
**10**	Δ12-13LL-NH_2	$C_{74}H_{110}N_{18}O_{16}$	1506.8347	754.4248 $[M+2H]^{2+}$	754.4247	24
**11**	Δ11-12LB-NH_2	$C_{69}H_{101}N_{17}O_{14}$	1391.7714	1392.7838 $[M+H]^{+}$	1392.7787	13
**12**	Δ11-13LL-NH_2	$C_{70}H_{104}N_{16}O_{14}$	1392.7918	697.4037 $[M+2H]^{2+}$	697.4032	20
**13**	Δ10-12LB-NH_2	$C_{63}H_{90}N_{16}O_{13}$	1278.6873	1279.7019 $[M+H]^{+}$	1279.6946	14
**14**	Δ10-13LL-NH_2	$C_{64}H_{93}N_{15}O_{13}$	1279.7077	640.8614 $[M+2H]^{2+}$	640.8612	29
**15**	Δ9-12LB-NH_2	$C_{59}H_{83}N_{15}O_{11}$	1177.6396	1178.6526 $[M+H]^{+}$	1178.6469	13
**16**	Δ9-13LL-NH_2	$C_{60}H_{86}N_{14}O_{11}$	1178.6600	1179.6670 $[M+H]^{+}$	1179.6673	14
**17**	LB(D-Ala1)-NH_2	$C_{73}H_{107}N_{17}O_{17}$	1493.8031	747.4174 $[M+2H]^{2+}$	747.4175	11
**18**	LB(D-Ala2)-NH_2	$C_{68}H_{104}N_{18}O_{17}$	1444.7827	1466.7829 $[M+Na]^{+}$	1466.7885	6
**19**	LB(D-Ala3)-NH_2	$C_{66}H_{103}N_{17}O_{18}$	1421.7667	1421.7842 $[M+H]^{+}$	1421.7905	9
**20**	LB(D-Ala4)-NH_2	$C_{72}H_{103}N_{17}0_{18}$	1493.7667	747.4002 $[M+2H]^{2+}$	747.3992	3
**21**	LB(Ala5)-NH_2	$C_{72}H_{103}N_{17}O_{18}$	1493.7667	747.3993 $[M+2H]^{2+}$	747.3992	8
**22**	LB(Ala6)-NH_2	$C_{75}H_{110}N_{18}O_{18}$	1550.8245	775.9287 $[M+2H]^{2+}$	775.9281	7
**23**	LB(D-Ala7)-NH_2	$C_{72}H_{102}N_{17}O_{18}$	1493.7667	747.3990 $[M+2H]^{2+}$	747.3992	8
**24**	LB(Ala8)-NH_2	$C_{66}H_{103}N_{17}O_{18}$	1421.7667	711.3997 $[M+2H]^{2+}$	711.3992	19
**25**	LB(Ala9)-NH_2	$C_{73}H_{106}N_{18}O_{17}$	1506.7983	1528.7998 $[M+Na]^{+}$	1528.8041	17
**26**	LB(Ala10)-NH_2	$C_{71}H_{102}N_{18}O_{18}$	1494.7619	1494.7811 $[M+H]^{+}$	1494.7858	9
**27**	LB(Ala11)-NH_2	$C_{75}H_{110}N_{18}O_{18}$	1550.8245	775.9284 $[M+2H]^{2+}$	775.9281	7
**28**	LB(Ala12)-NH_2	$C_{74}H_{108}N_{18}O_{17}$	1520.8140	760.9227 $[M+2H]^{2+}$	760.9228	15
**29**	LL(D-Ala1)-NH_2	$C_{78}H_{116}N_{20}O_{17}$	1604.8827	803.4487 $[M+2H]^{2+}$	803.4487	34
**30**	LL(D-Ala2)-NH_2	$C_{72}H_{112}N_{20}O_{17}$	1528.8514	765.4331 $[M+2H]^{2+}$	765.4330	31
**31**	LL(D-Ala3)-NH_2	$C_{70}H_{111}N_{19}O_{18}$	1505.8354	753.9254 $[M+2H]^{2+}$	753.9250	37
**32**	LL(D-Ala4)-NH_2	$C_{76}H_{111}N_{19}O_{18}$	1577.8354	789.9256 $[M+2H]^{2+}$	789.9250	27
**33**	LL(Ala5)-NH_2	$C_{76}H_{111}N_{19}O_{18}$	1577.8354	789.9255 $[M+2H]^{2+}$	789.9250	30
**34**	LL(Ala6)-NH_2	$C_{79}H_{118}N_{20}O_{18}$	1634.8933	818.4544 $[M+2H]^{2+}$	818.4539	34
**35**	LL(D-Ala7)-NH_2	$C_{76}H_{111}N_{19}O_{18}$	1577.8354	789.9256 $[M+2H]^{2+}$	789.9250	28
**36**	LL(Ala8)-NH_2	$C_{70}H_{111}N_{19}O_{18}$	1505.8354	753.9254 $[M+2H]^{2+}$	753.9250	31
**37**	LL-(Ala9)-NH_2	$C_{77}H_{114}N_{20}O_{17}$	1590.8671	796.4409 $[M+2H]^{2+}$	796.4408	43
**38**	LL(Ala10)-NH_2	$C_{75}H_{110}N_{20}O_{18}$	1578.8307	790.4229 $[M+2H]^{2+}$	790.4226	34
**39**	LL(Ala11)-NH_2	$C_{77}H_{115}N_{19}O_{17}$	1577.8718	789.9429 $[M+2H]^{2+}$	789.9432	20
**40**	LL(Ala12)-NH_2	$C_{79}H_{118}N_{20}O_{18}$	1634.8933	818.4546 $[M+2H]^{2+}$	818.4539	29
**41**	LL(Ala13)-NH_2	$C_{79}H_{118}N_{20}O_{18}$	1634.8933	818.4541 $[M+2H]^{2+}$	818.4539	32

**42**	LL(Leu9)-NH_2	$C_{80}H_{120}N_{20}O_{17}$	1632.9140	817.4644 $[M+2H]^{2+}$	817.4643	10
**43**	LL(Phe9)-NH_2	$C_{83}H_{118}N_{20}O_{17}$	1666.8984	834.4563 $[M+2H]^{2+}$	834.4565	22
**44**	LL(Met9)-NH_2	$C_{79}H_{118}N_{20}O_{17}S$	1650.8705	826.4424 $[M+2H]^{2+}$	826.4425	17
**45**	LL(Trp9)-NH_2	$C_{85}H_{119}N_{21}O_{17}$	1705.9093	853.9619 $[M+2H]^{2+}$	853.9619	20
**46**	LL(Ser9)-NH_2	$C_{77}H_{114}N_{20}O_{18}$	1606.8620	804.4386 $[M+2H]^{2+}$	804,4383	22
**47**	LL(Asn9)-NH_2	$C_{78}H_{115}N_{21}O_{18}$	1633.8729	817.9438 $[M+2H]^{2+}$	817.9437	20
**48**	LL(Gln9)-NH_2	$C_{79}H_{117}N_{21}O_{18}$	1647.8885	824.9519 $[M+2H]^{2+}$	824.9516	17
**49**	LL(MeAbu9)-NH_2	$C_{78}H_{115}N_{23}O_{17}$	1645.8841	823.9495 $[M+2H]^{2+}$	823.9494	13
**50**	LL(Dap[Alloc]9)-NH_2	$C_{81}H_{119}N_{21}O_{19}$	1689.8991	845.9574 $[M+2H]^{2+}$	845.9569	15
**51**	LL(Glu[OAll]9)-NH_2	$C_{82}H_{120}N_{20}O_{19}$	1688.9039	845.4595 $[M+2H]^{2+}$	845.4592	14
**52**	LL(Dap9)-NH_2	$C_{77}H_{115}N_{21}O_{17}$	1605.8780	803.9465 $[M+2H]^{2+}$	803.9463	21
**53**	Δ12LB(Leu9)-NH_2	$C_{73}H_{108}N_{18}O_{14}$	1460.8292	1461.8273 $[M+H]^{+}$	1461.8365	38
**54**	Δ12LB(Phe9)-NH_2	$C_{76}H_{106}N_{18}O_{14}$	1494.8136	748.4111 $[M+2H]^{2+}$	748.4141	30
**55**	Δ12LB(Met9)-NH_2	$C_{72}H_{106}N_{18}O_{14}S$	1478.7857	1479.7827 $[M+H]^{+}$	1479.7930	13
**56**	Δ12LB(Trp9)-NH_2	$C_{78}H_{107}N_{19}O_{14}$	1533.8245	512.2821 $[M+2H]^{2+}$	512.2717	24
**57**	Δ12LB(Ser9)-NH_2	$C_{70}H_{102}N_{18}O_{16}$	1434.7772	1435.7797 $[M+H]^{+}$	1435.7845	42
**58**	Δ12LB(Asn9)-NH_2	$C_{71}H_{103}N_{19}O_{15}$	1461.7881	1484.7651 $[M+H]^{+}$	1484.7773	12
**59**	Δ12LB(Gln9)-NH_2	$C_{72}H_{105}N_{19}O_{15}$	1475.8038	492.9408 $[M+3H]^{3+}$	493.9358	24
**60**	Δ12LB(MeAbu9)-NH_2	$C_{71}H_{103}N_{21}O_{14}$	1473.7993	737.9129 $[M+H]^{+}$	737.9069	22
**61**	Δ12LB(Dap[Alloc]9)-NH_2	$C_{74}H_{107}N_{19}O_{16}$	1517.8143	749.9157 $[M+2H]^{2+}$	749.9144	33
**62**	Δ12LB(Glu[OAll]9)-NH_2	$C_{75}H_{108}N_{18}O_{16}$	1516.8191	506.6093 $[M+3H]^{3+}$	506.6136	21
**63**	Δ12LB(Asp9)-NH_2	$C_{71}H_{102}N_{18}O_{16}$	1462.7721	732.3873 $[M+2H]^{2+}$	732.3933	27
**64**	Δ12LB(Dap9)-NH_2	$C_{70}H_{103}N_{19}O_{14}$	1433.7932	717.9044 $[M+2H]^{2+}$	717.9039	22

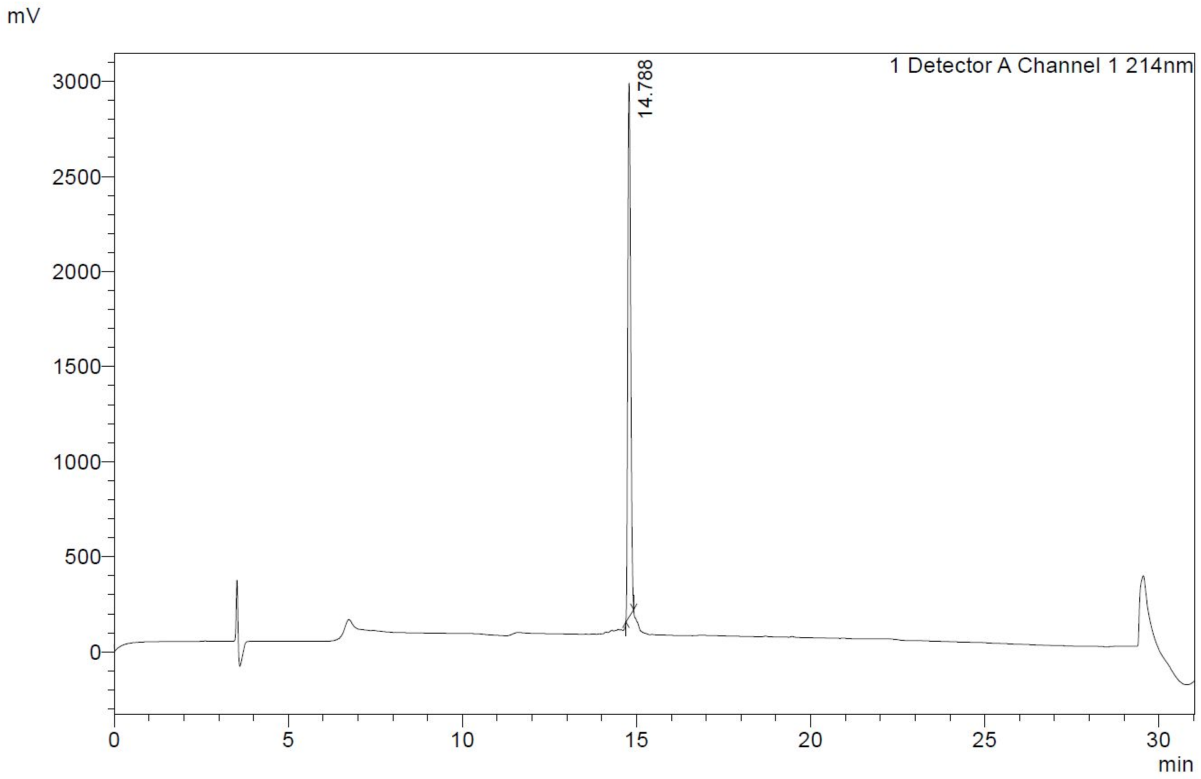

Fig. S2. HPLC trace showing the reinjection of purified LL-OH (**4**). The peptide eluted as a single peak at 14.79 min using the HPLC **method E** outlined in part III.

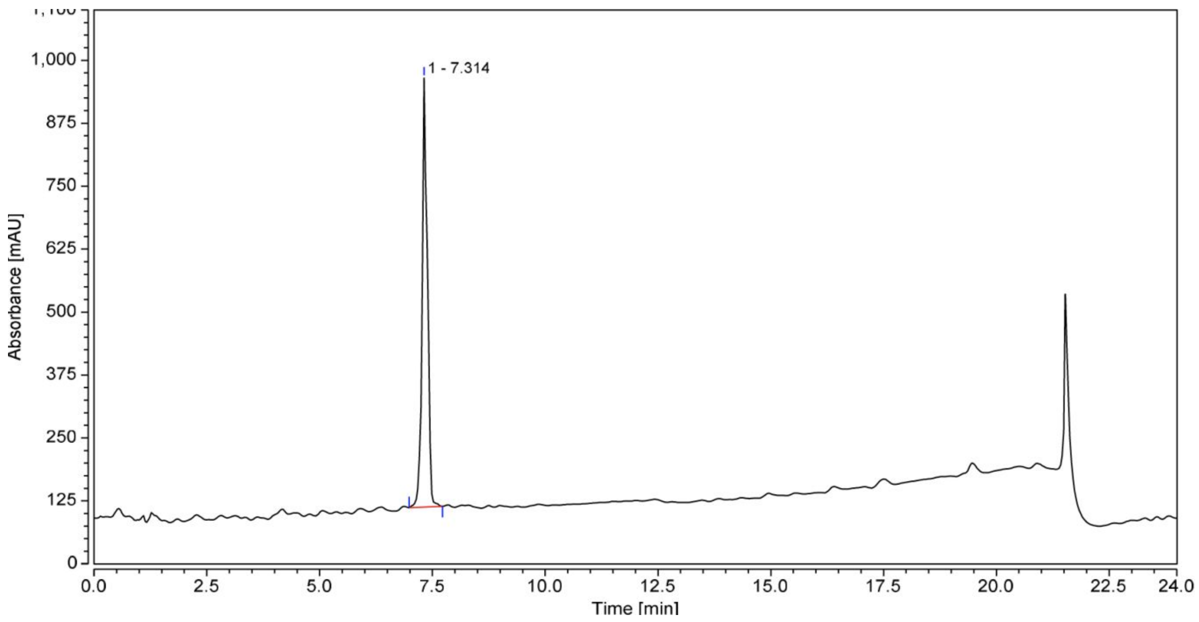

Fig. S3. HPLC trace showing the reinjection of purified LB-OH (**5**). The peptide eluted as a single peak at 7.31 min using the HPLC **method C** outlined in part III.

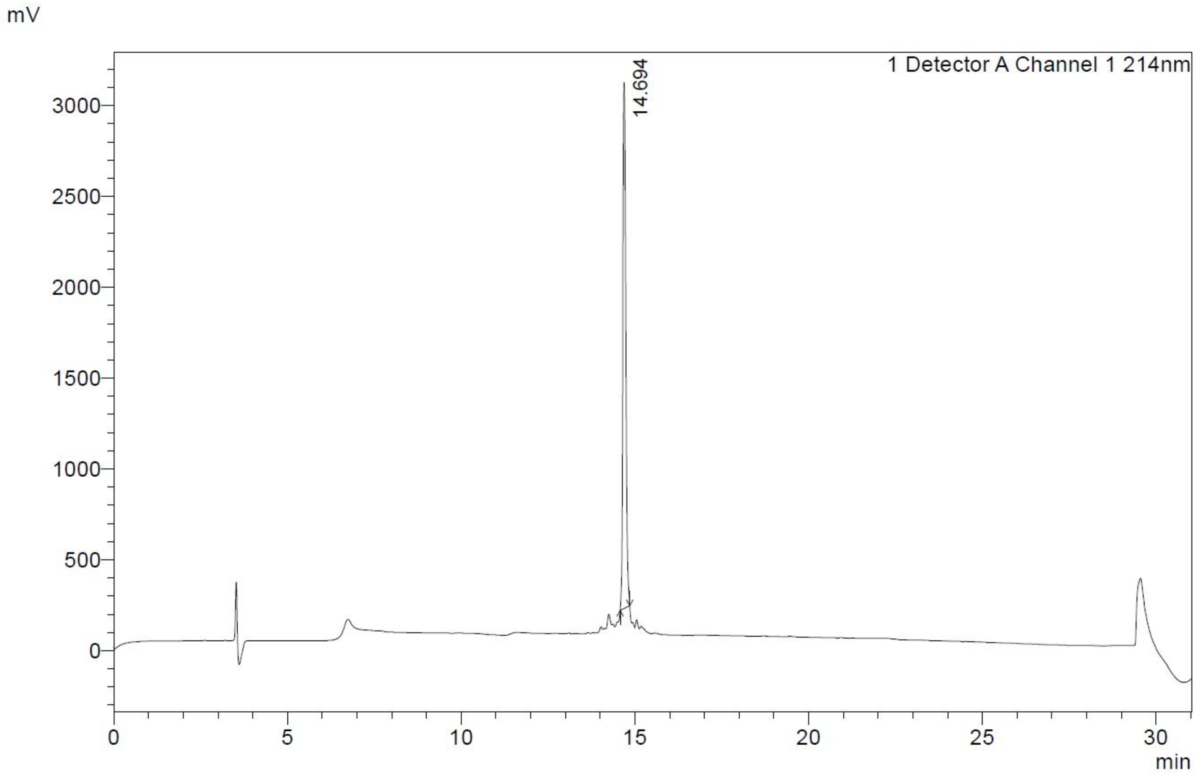

Fig. S4. HPLC trace showing the reinjection of purified LL-NH_2 (**6**). The peptide eluted as a single peak at 14.69 min using the HPLC **method E** outlined in part III.

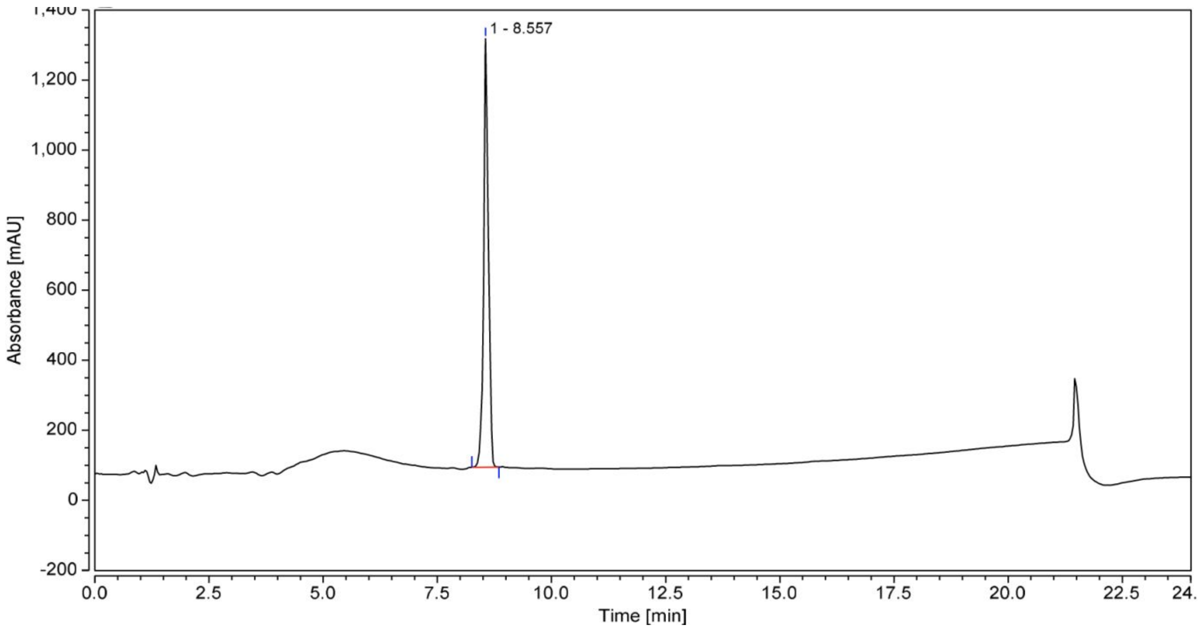

Fig. S5. HPLC trace showing the reinjection of purified LB-NH_2 (**7**). The peptide eluted as a single peak at 8.56 min using the HPLC **method C** outlined in part III.

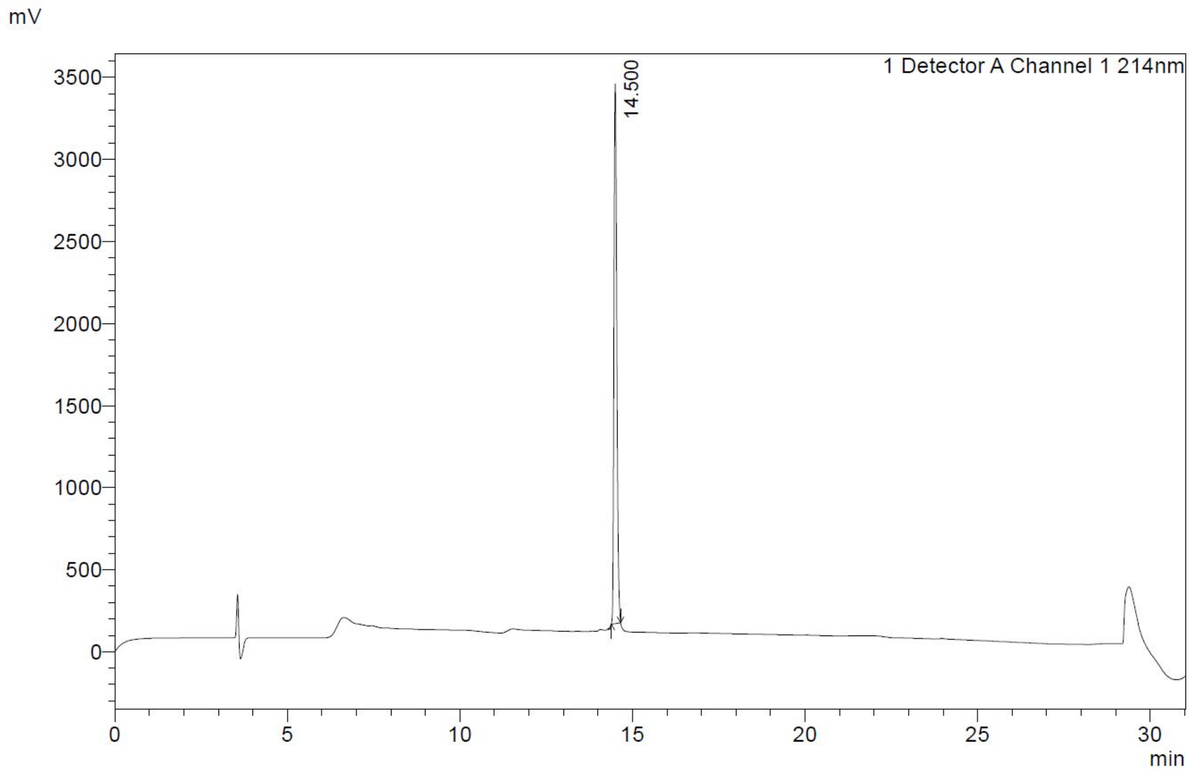

Fig. S6. HPLC trace showing the reinjection of purified Δ13LL-NH_2 (**8**). The peptide eluted as a single peak at 14.50 min using the HPLC **method E** outlined in part III.

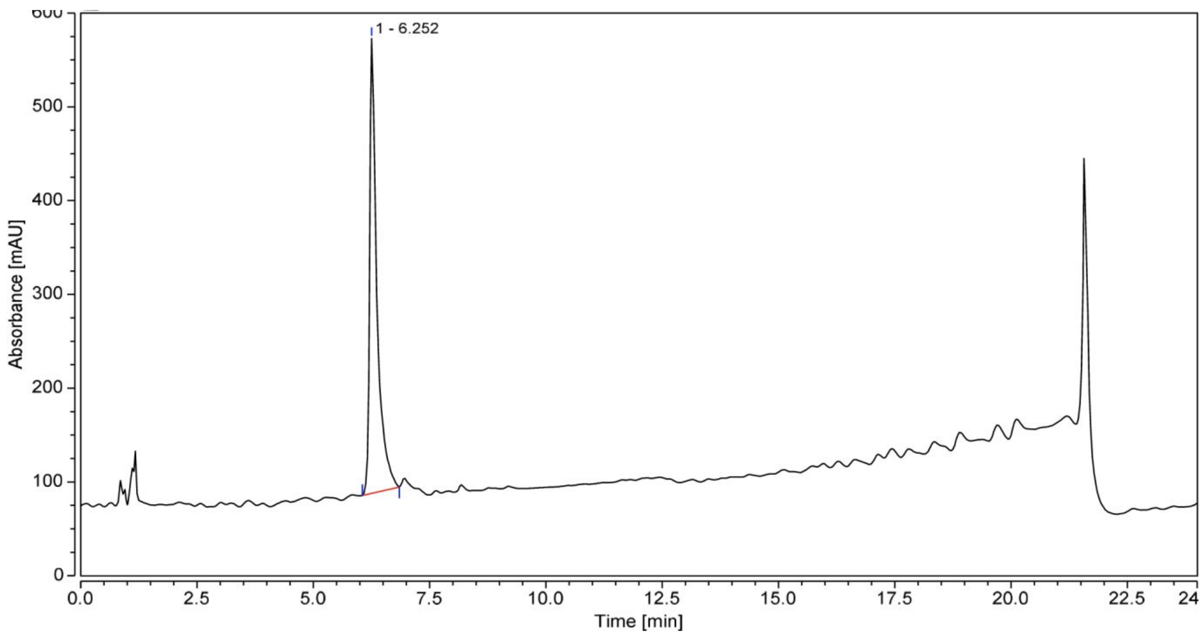

Fig. S7. HPLC trace showing the reinjection of purified Δ12LB-NH_2 (**9**). The peptide eluted as a single peak at 6.25 min using the HPLC **method C** outlined in part III.

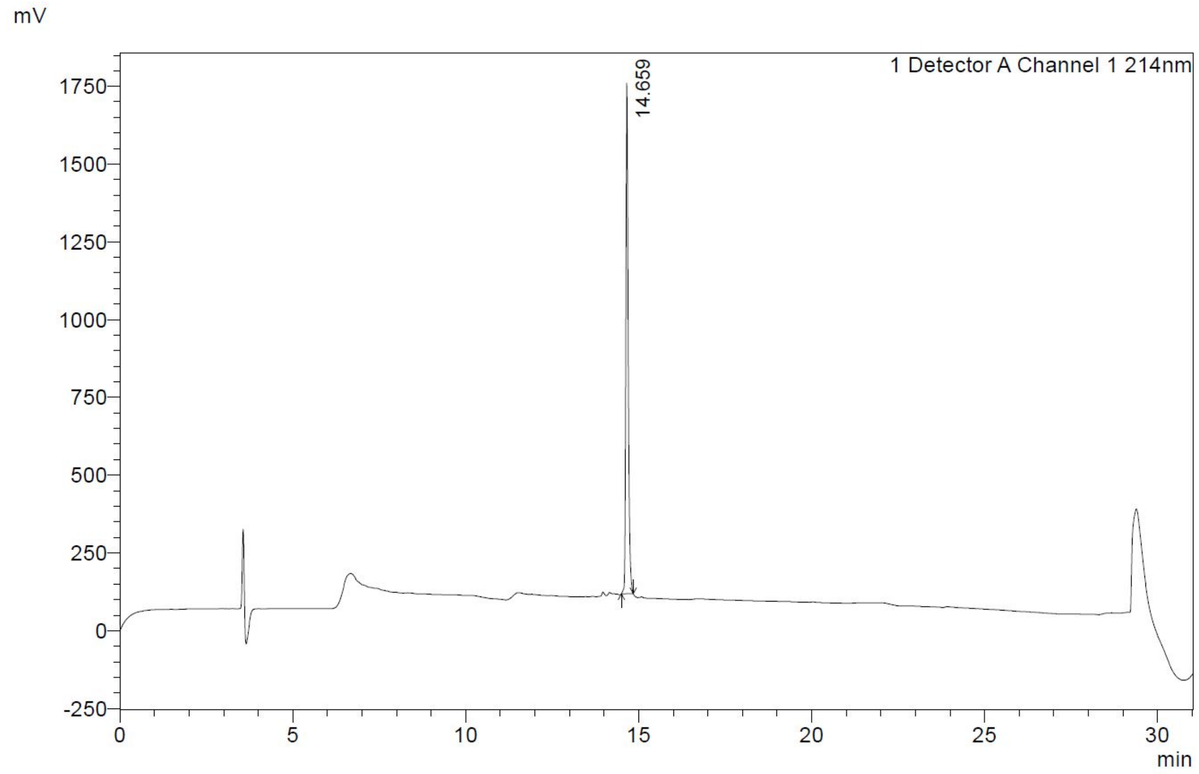

Fig. S8. HPLC trace showing the reinjection of purified Δ12-13LL-NH_2 (**10**). The peptide eluted as a single peak at 14.66 min using the HPLC **method E** outlined in part III.

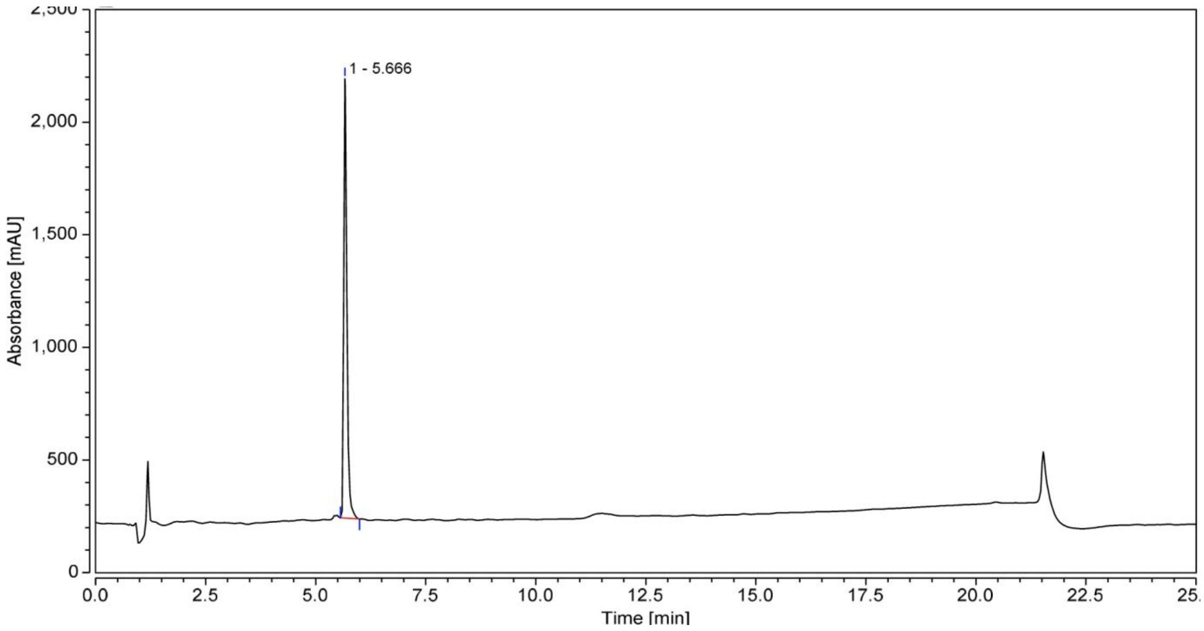

Fig. S9. HPLC trace showing the reinjection of purified Δ11-12LB-NH_2 (**11**). The peptide eluted as a single peak at 5.67 min using the HPLC **method C** outlined in part III.

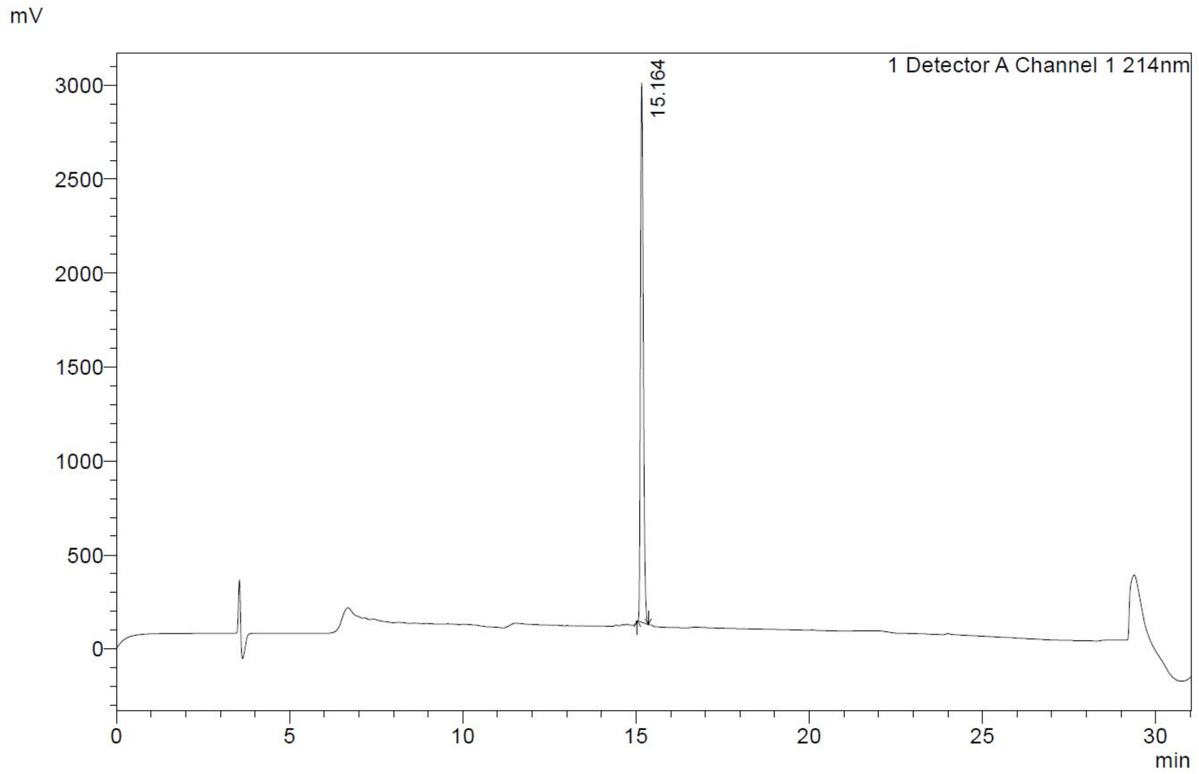

Fig. S10. HPLC trace showing the reinjection of purified Δ11-13LL-NH_2 (**12**). The peptide eluted as a single peak at 15.16 min using the HPLC **method E** outlined in part III.

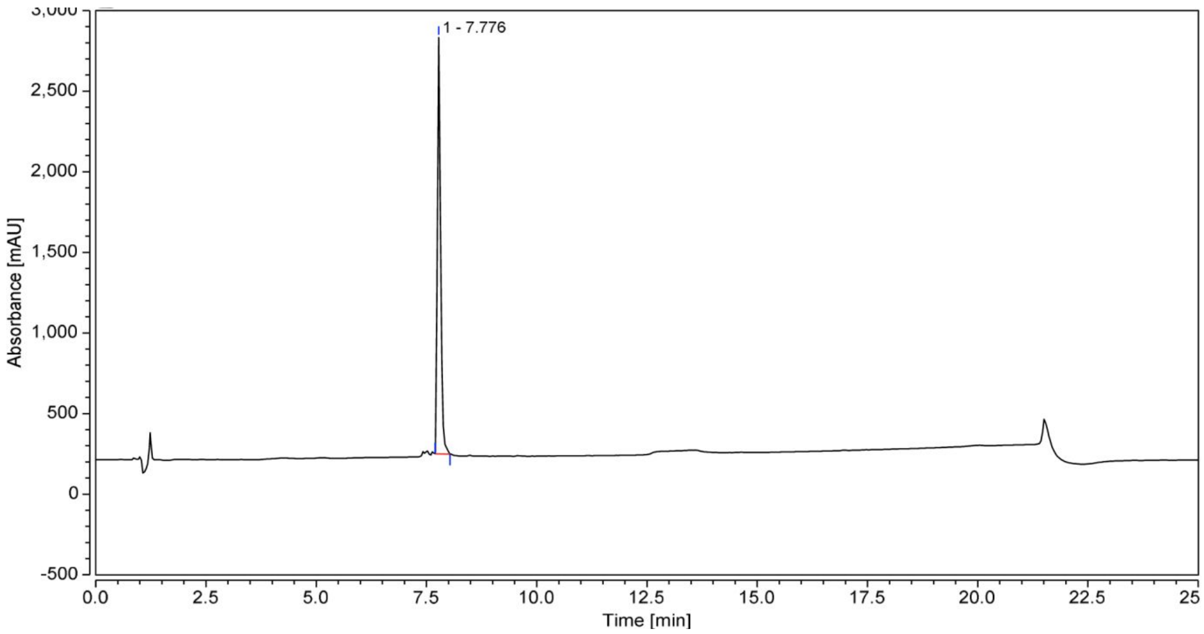

Fig. S11. HPLC trace showing the reinjection of purified Δ10-12LB-NH_2 (**13**). The peptide eluted as a single peak at 7.78 min using the HPLC **method C** outlined in part III.

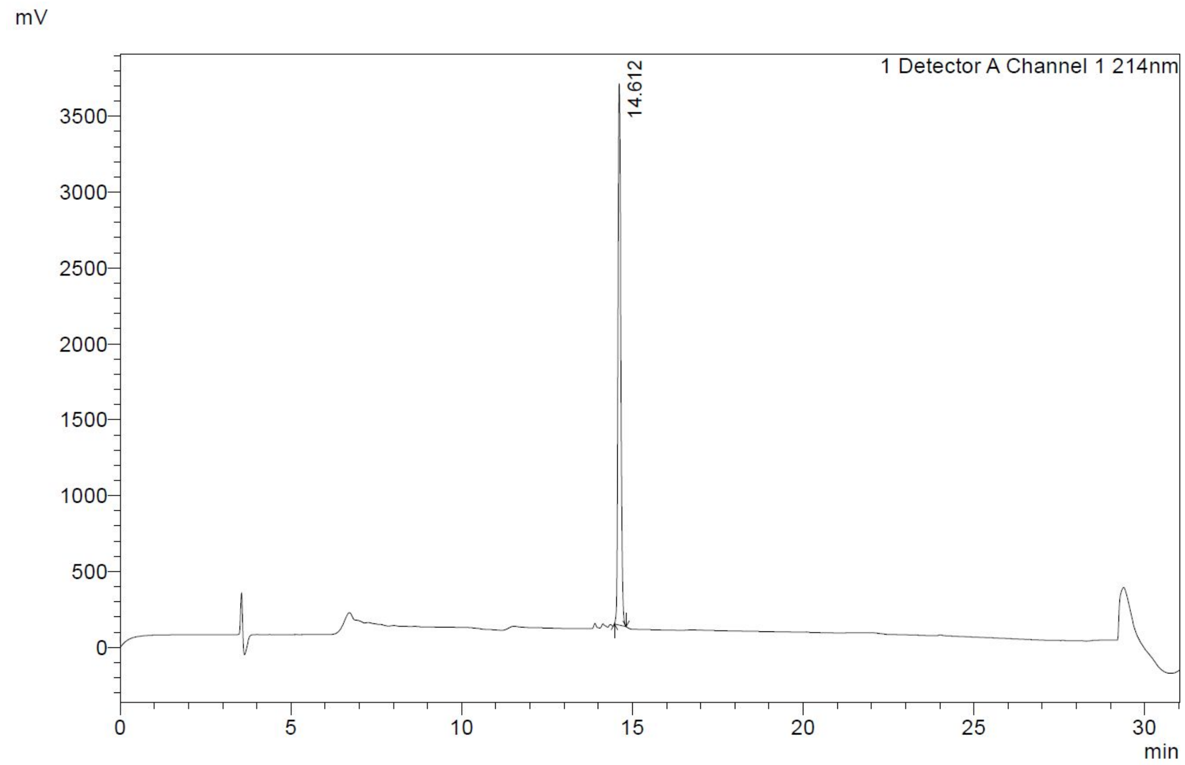

Fig. S12. HPLC trace showing the reinjection of purified Δ10-13LL-NH_2 (**14**). The peptide eluted as a single peak at 14.61 min using the HPLC **method E** outlined in part III.

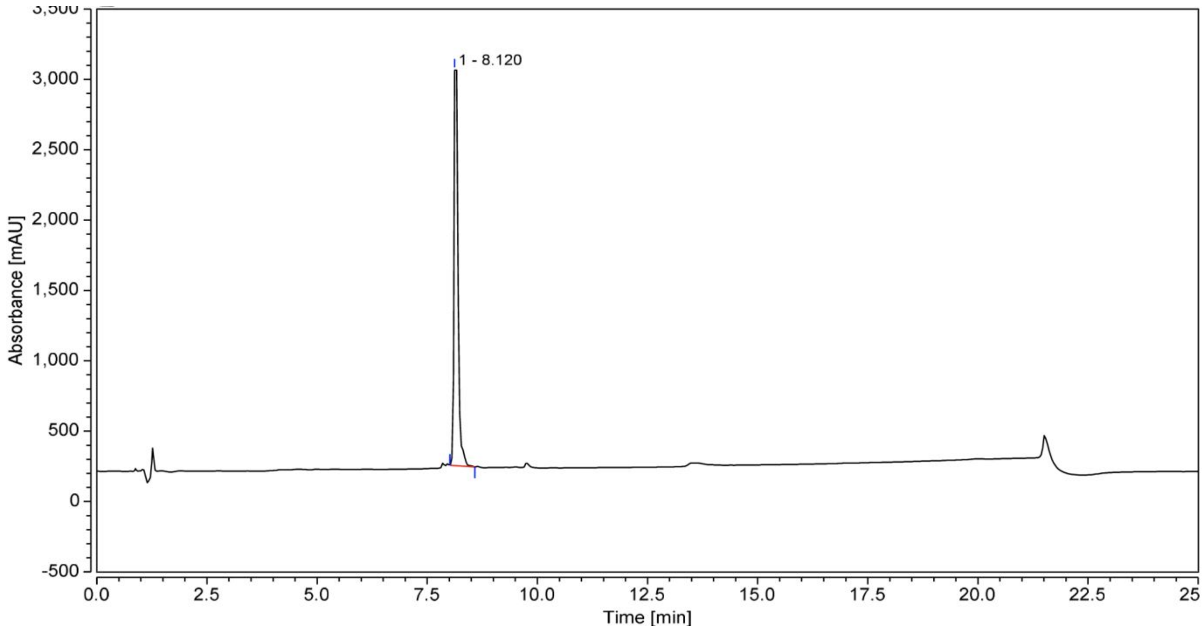

Fig. S13. HPLC trace showing the reinjection of purified Δ9-12LB-NH_2 (**15**). The peptide eluted as a single peak at 8.12 min using the HPLC **method C** outlined in part III.

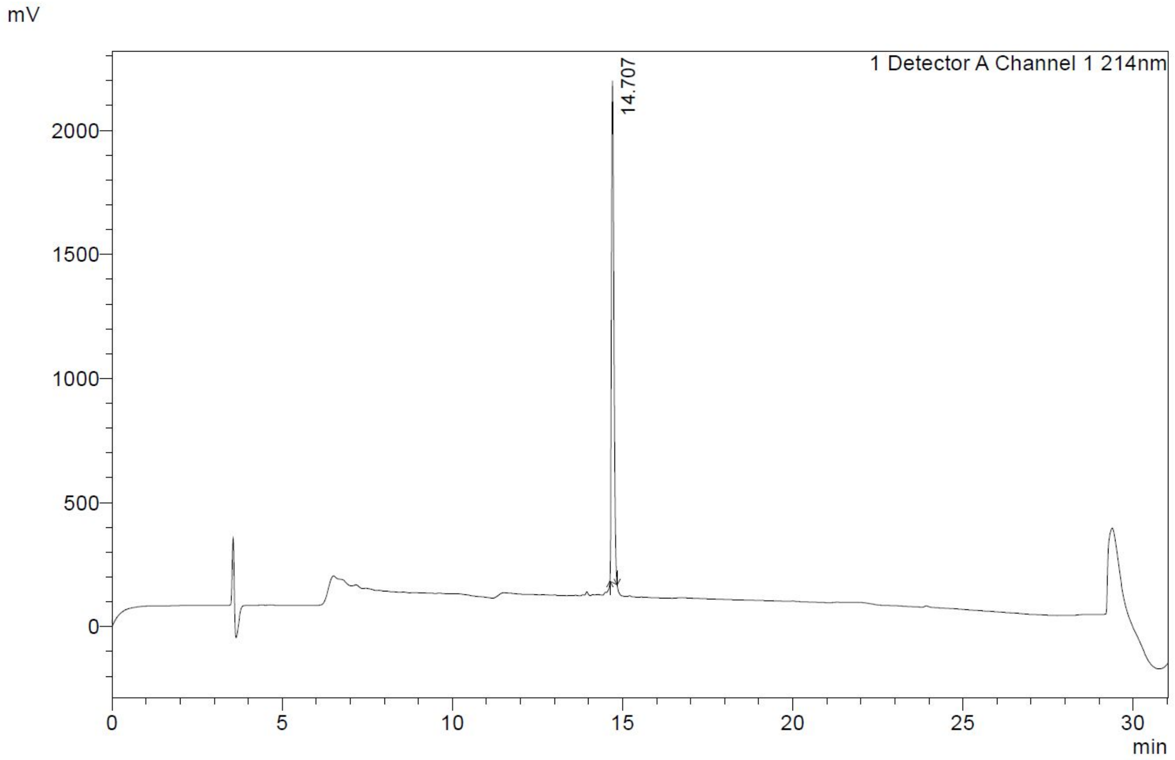

Fig. S14. HPLC trace showing the reinjection of purified Δ9-13LL-NH_2 (**16**). The peptide eluted as a single peak at 14.71 min using the HPLC **method E** outlined in part III.

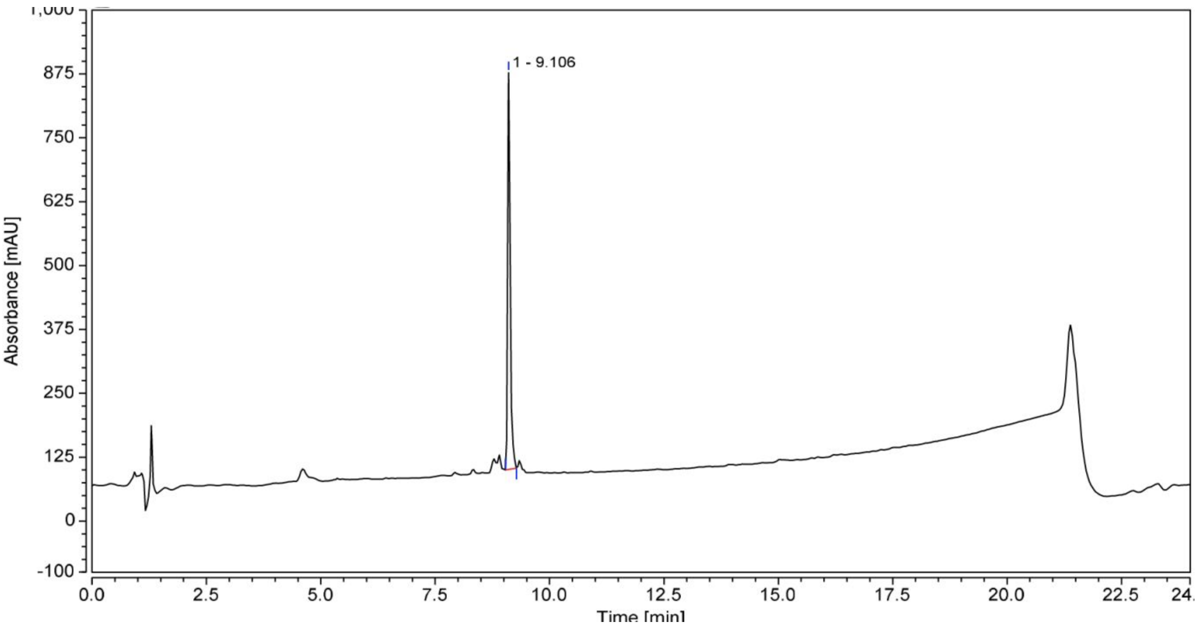

Fig. S15. HPLC trace showing the reinjection of purified LB(D-Ala1)-NH_2 (**17**). The peptide eluted as a single peak at 9.11 min using the HPLC **method C** outlined in part III.

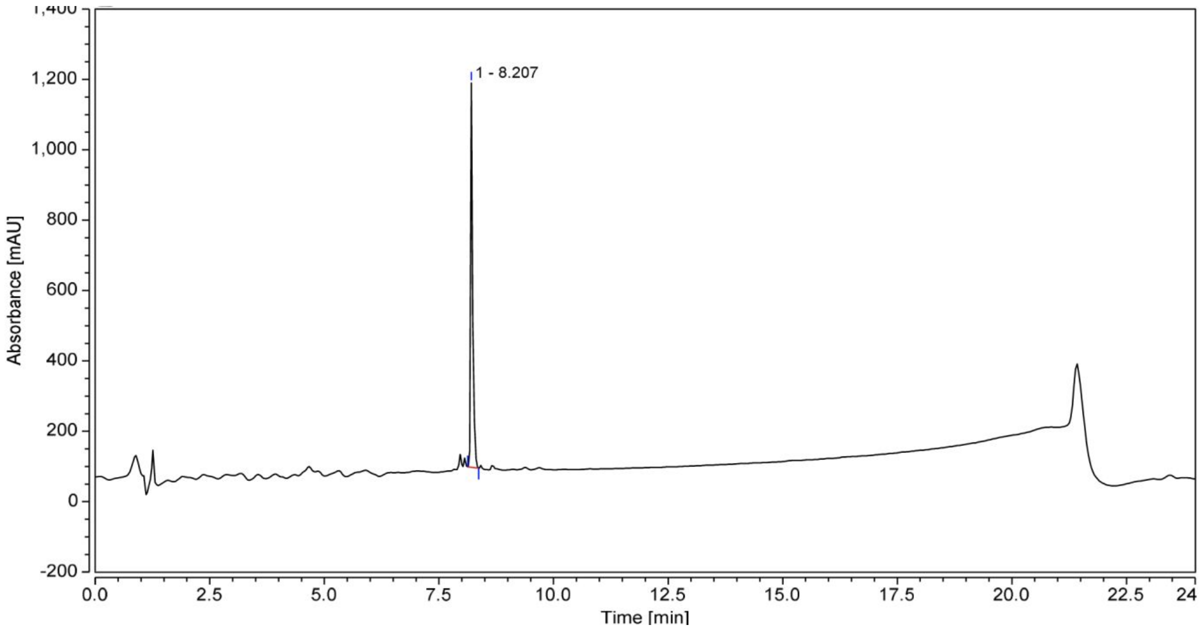

Fig. S16. HPLC trace showing the reinjection of purified LB(D-Ala2)-NH_2 (**18**). The peptide eluted as a single peak at 8.21 min using the HPLC **method C** outlined in part III.

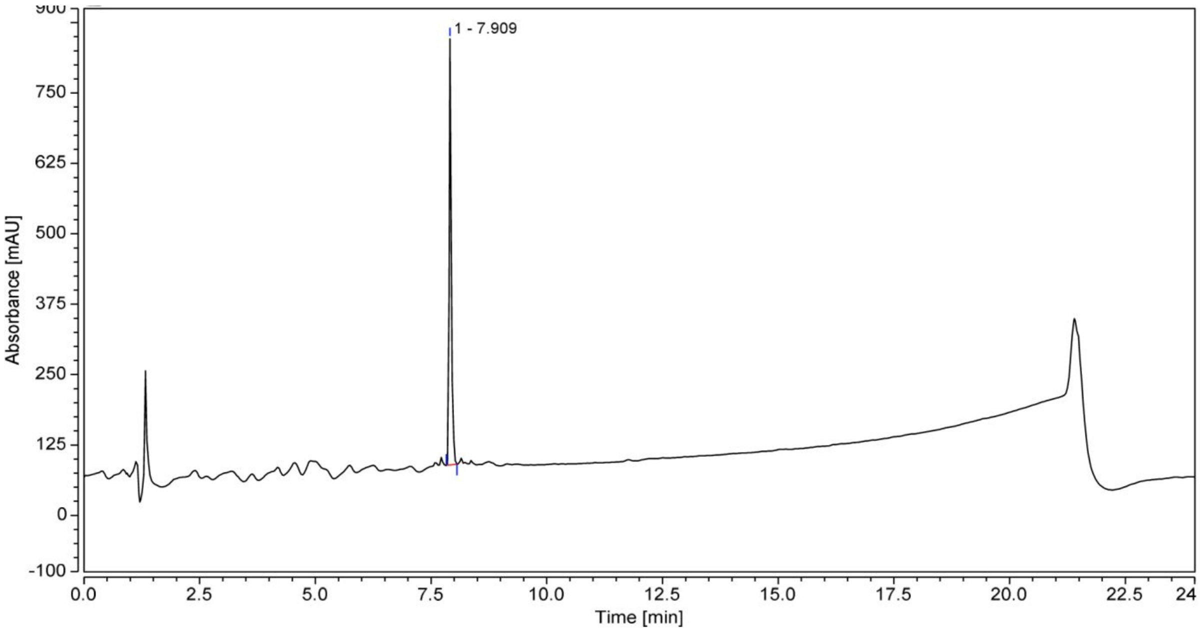

Fig. S17. HPLC trace showing the reinjection of purified LB(D-Ala3)-NH_2 (**19**). The peptide eluted as a single peak at 7.91 min using the HPLC **method C** outlined in part III.

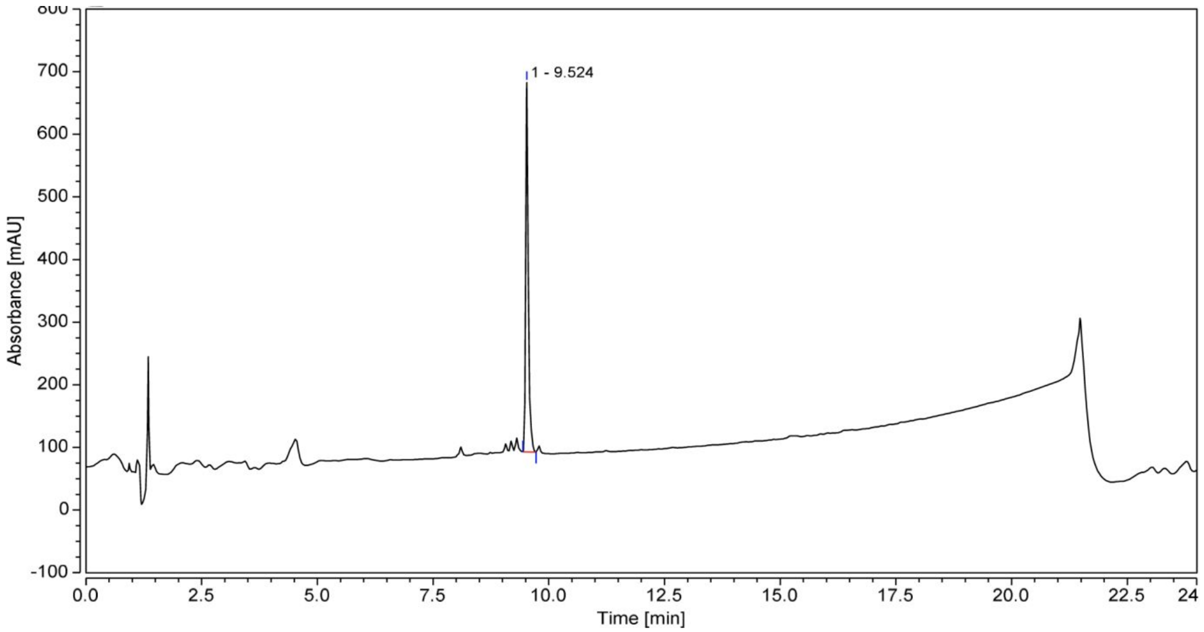

Fig. S18. HPLC trace showing the reinjection of purified LB(D-Ala4)-NH_2 (**20**). The peptide eluted as a single peak at 9.52 min using the HPLC **method C** outlined in part III.

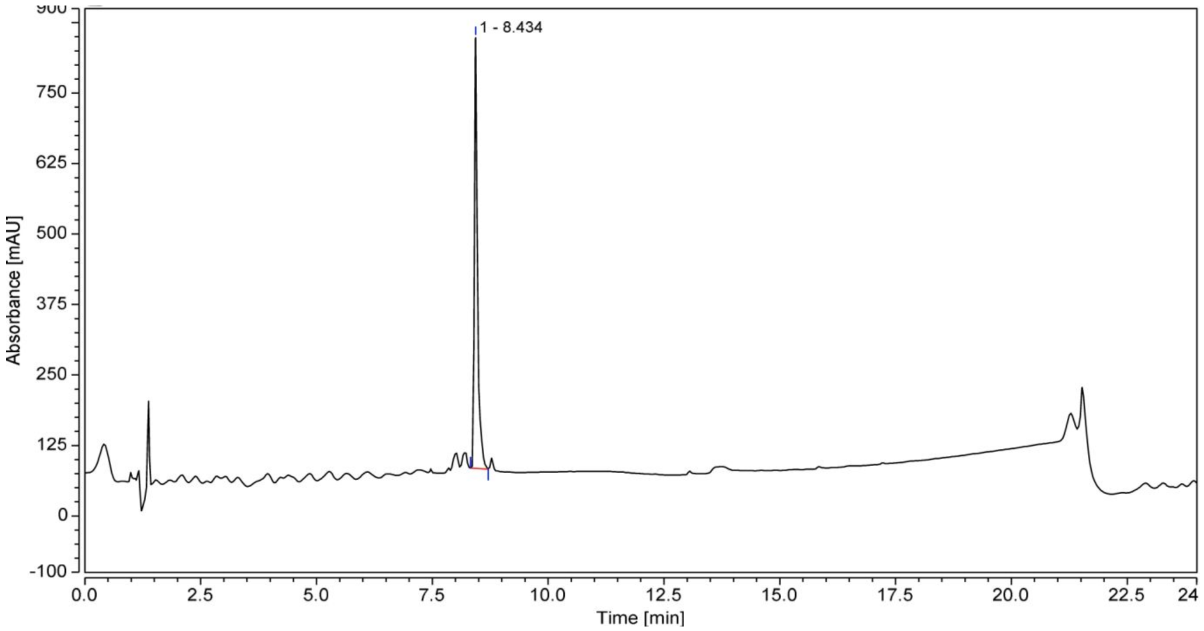

Fig. S19. HPLC trace showing the reinjection of purified LB(Ala5)-NH_2 (**21**). The peptide eluted as a single peak at 8.43 min using the HPLC **method C** outlined in part III.

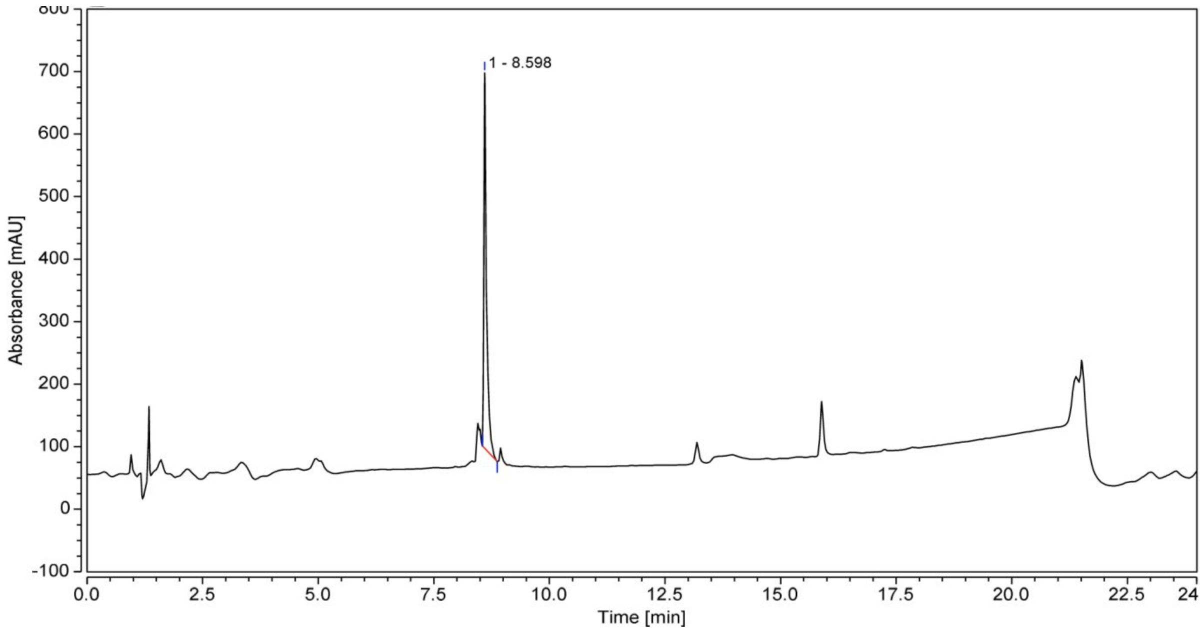

Fig. S20. HPLC trace showing the reinjection of purified LB(Ala6)-NH_2 (**22**). The peptide eluted as a single peak at 8.60 min using the HPLC **method C** outlined in part III.

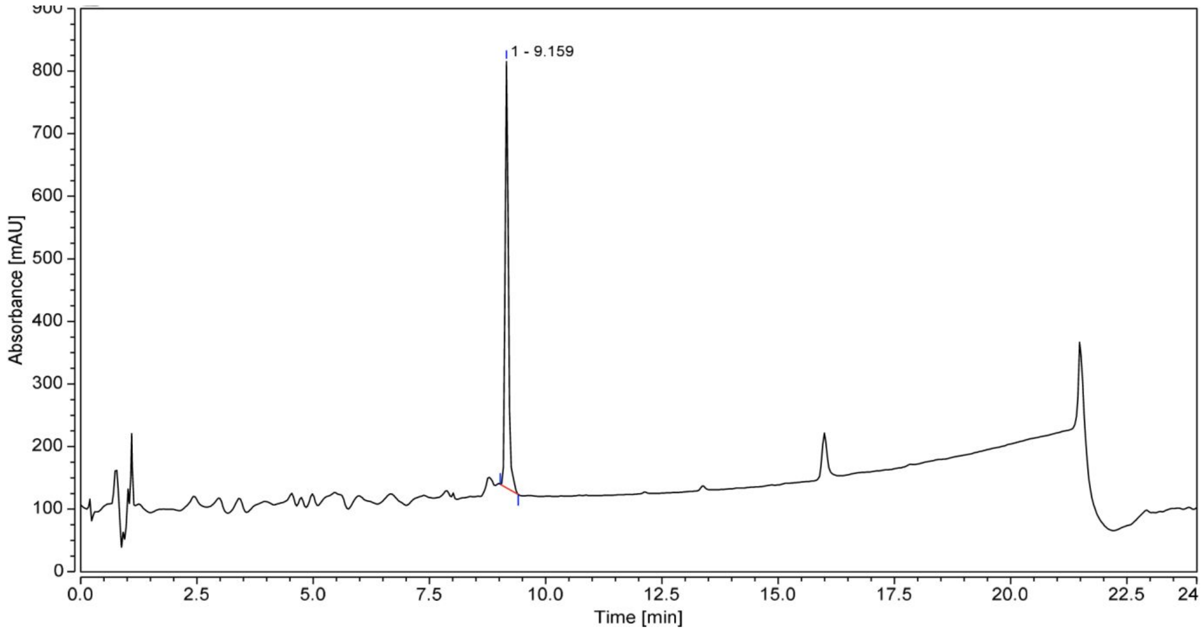

Fig. S21. HPLC trace showing the reinjection of purified LB(D-Ala7)-NH_2 (**23**). The peptide eluted as a single peak at 9.16 min using the HPLC **method C** outlined in part III.

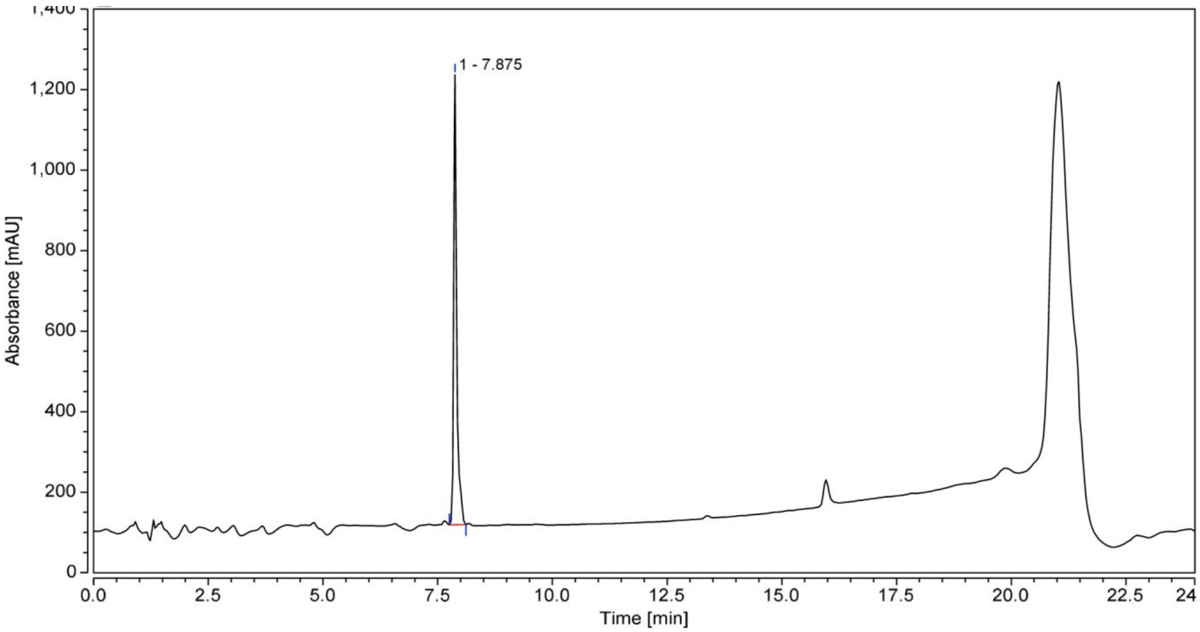

Fig. S22. HPLC trace showing the reinjection of purified LB(Ala8)-NH_2 (**24**). The peptide eluted as a single peak at 7.88 min using the HPLC **method C** outlined in part III.

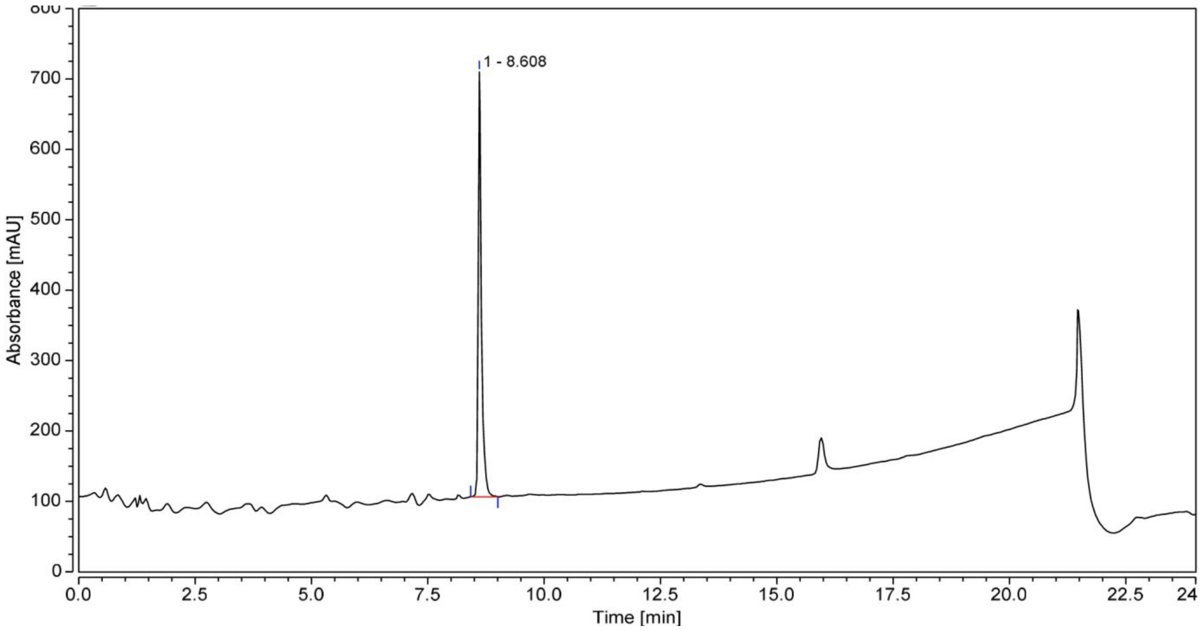

Fig. S23. HPLC trace showing the reinjection of purified LB(Ala9)-NH_2 (**25**). The peptide eluted as a single peak at 8.61 min using the HPLC **method C** outlined in part III.

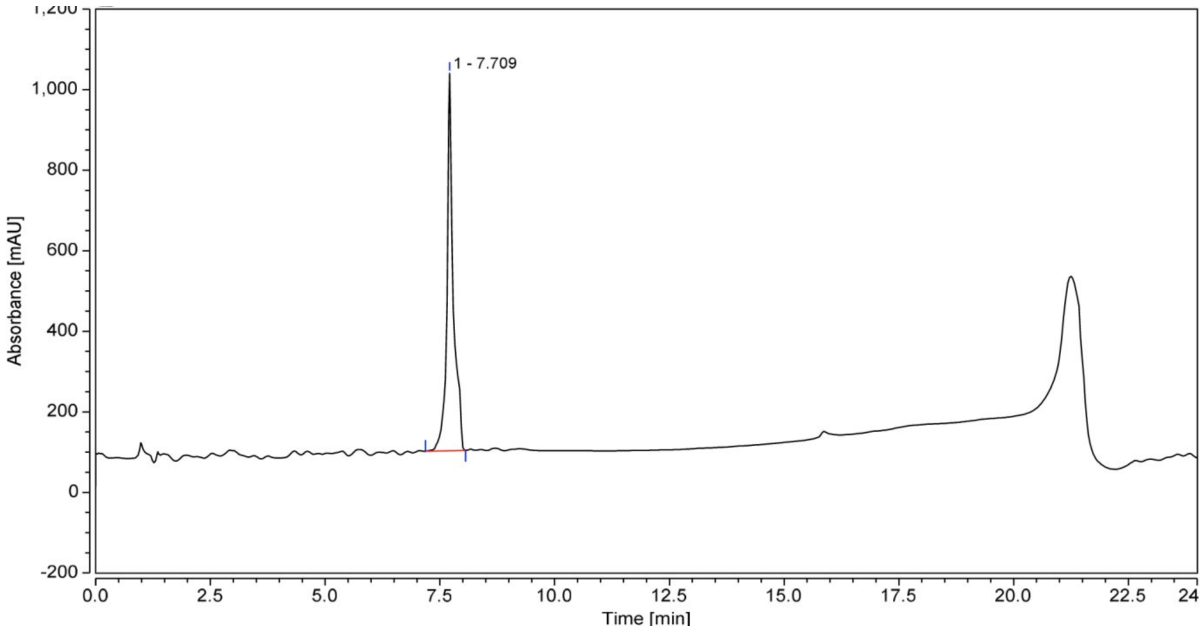

Fig. S24. HPLC trace showing the reinjection of purified LB(Ala10)-NH_2 (**26**). The peptide eluted as a single peak at 7.71 min using the HPLC **method C** outlined in part III.

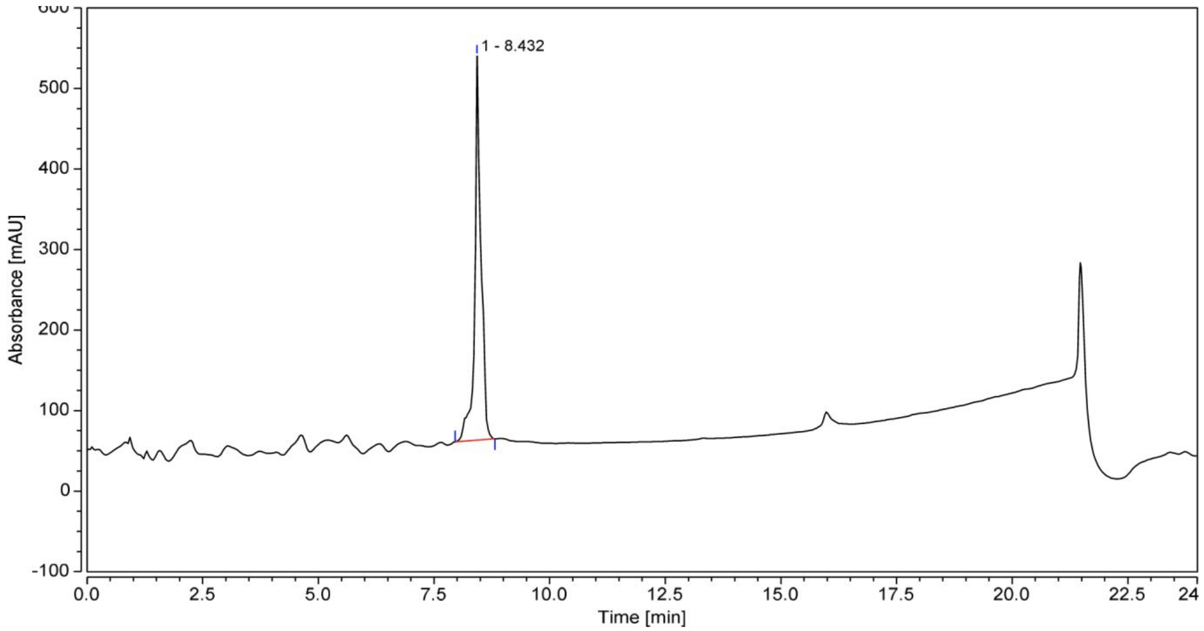

Fig. S25. HPLC trace showing the reinjection of purified LB(Ala11)-NH_2 (**27**). The peptide eluted as a single peak at 8.43 min using the HPLC **method C** outlined in part III.

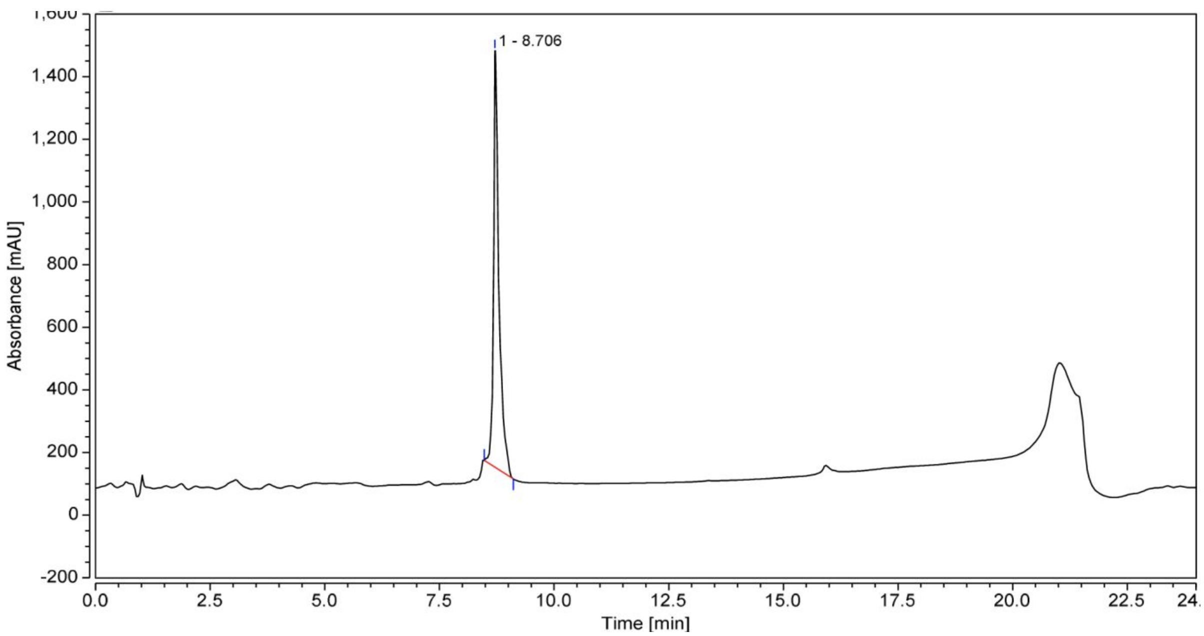

Fig. S26. HPLC trace showing the reinjection of purified LB(Ala12)-NH_2 (**28**). The peptide eluted as a single peak at 8.71 min using the HPLC **method C** outlined in part III.

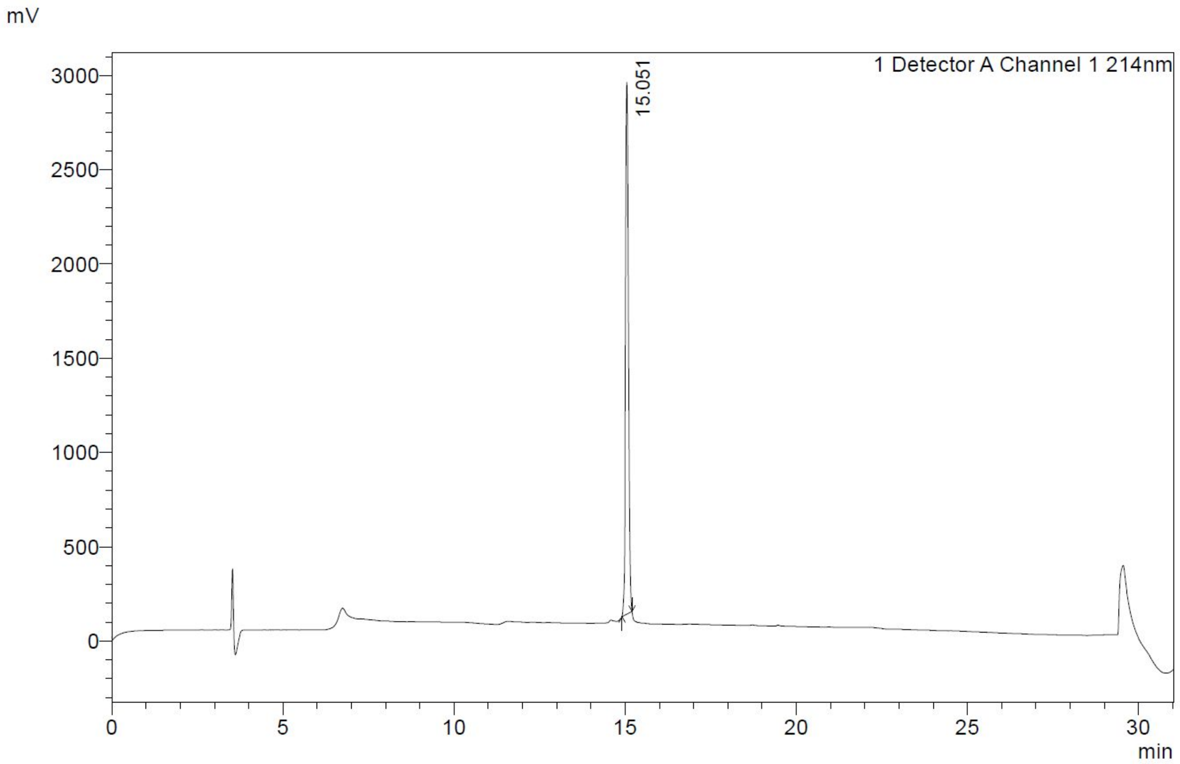

Fig. S27. HPLC trace showing the reinjection of purified LL(D-Ala1)-NH_2 (**29**). The peptide eluted as a single peak at 15.05 min using the HPLC **method E** outlined in part III.

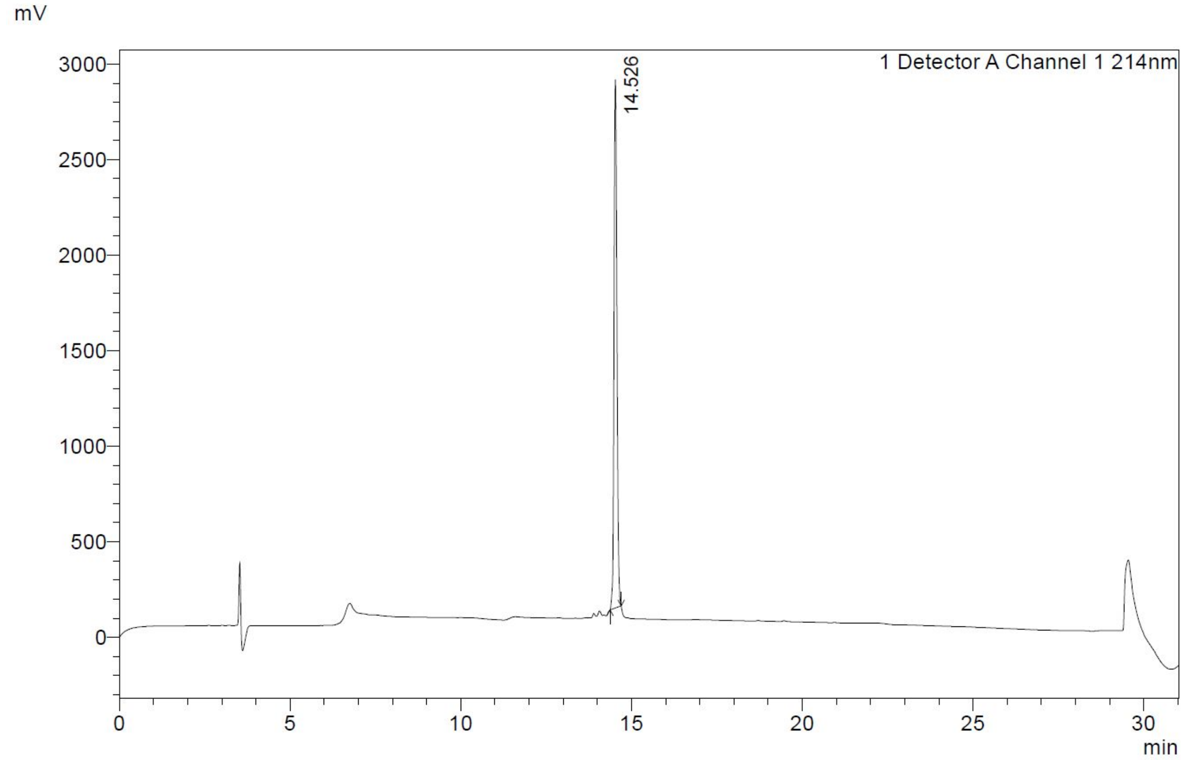

Fig. S28. HPLC trace showing the reinjection of purified LL(D-Ala2)-NH_2 (**30**). The peptide eluted as a single peak at 14.53 min using the HPLC **method E** outlined in part III.

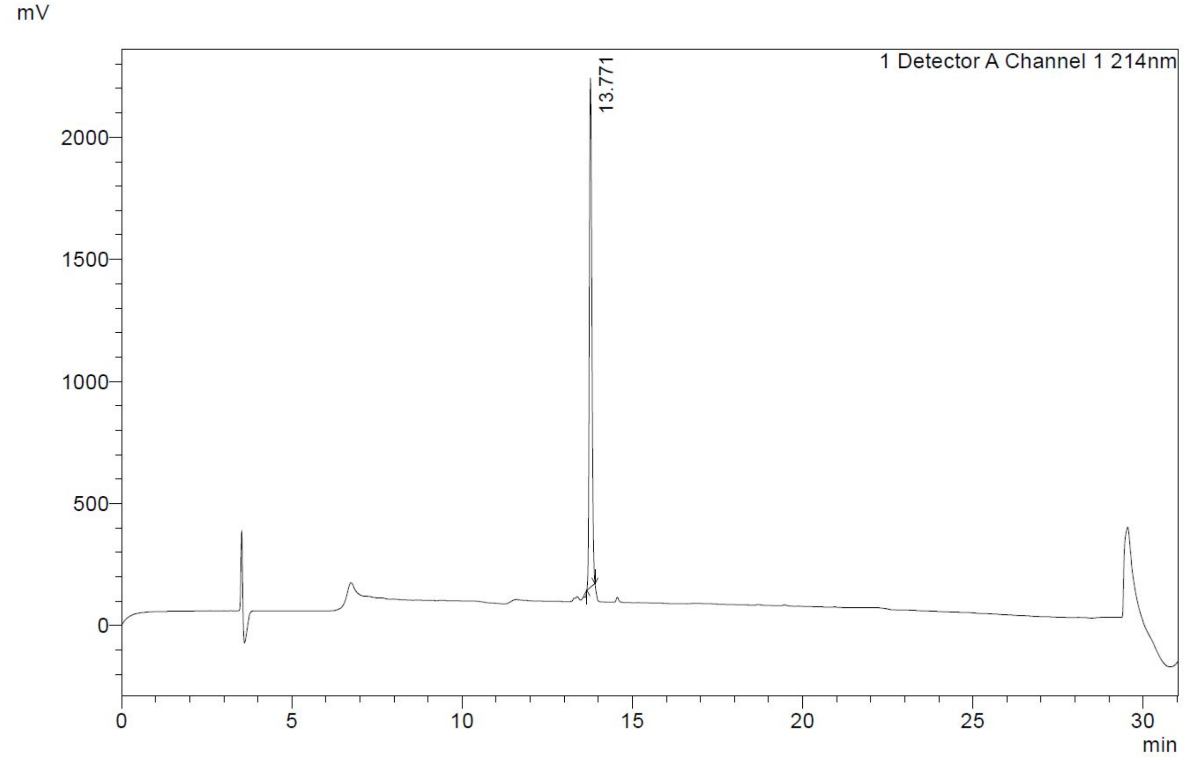

Fig. S29. HPLC trace showing the reinjection of purified LL(D-Ala3)-NH_2 (**31**). The peptide eluted as a single peak at 13.77 min using the HPLC **method E** outlined in part III.

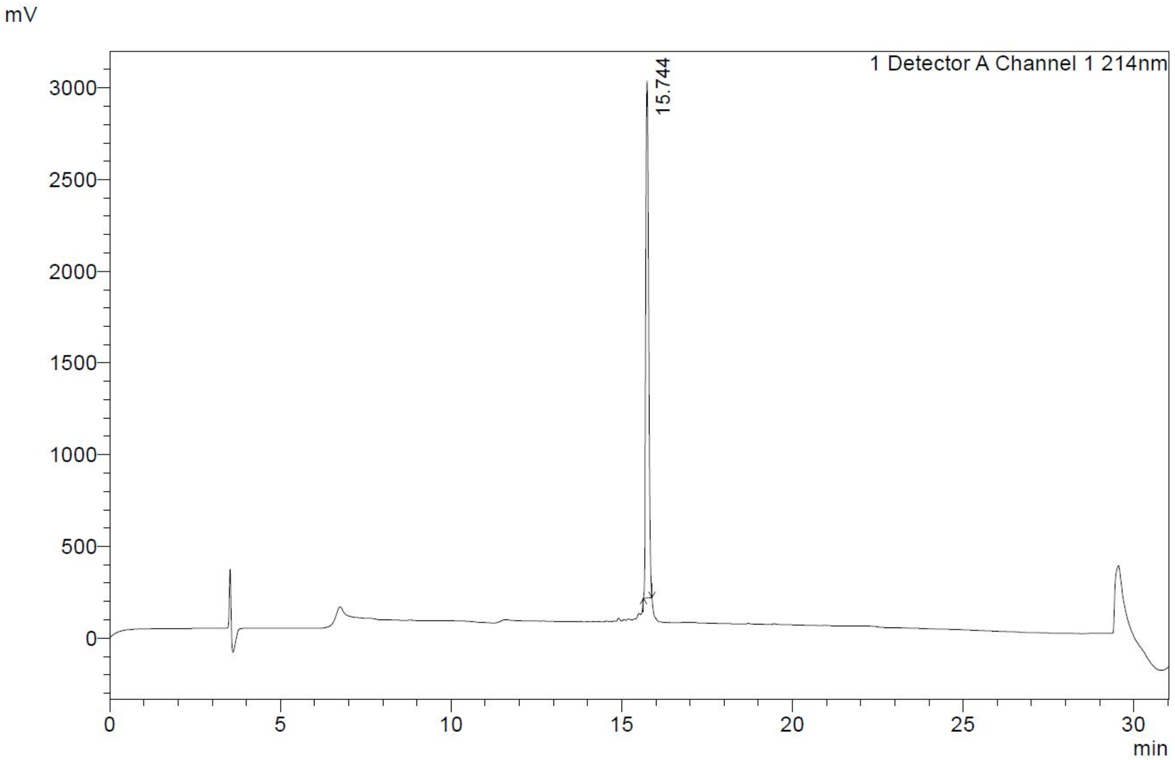

Fig. S30. HPLC trace showing the reinjection of purified LL(D-Ala4)-NH_2 (**32**). The peptide eluted as a single peak at 15.74 min using the HPLC **method E** outlined in part III.

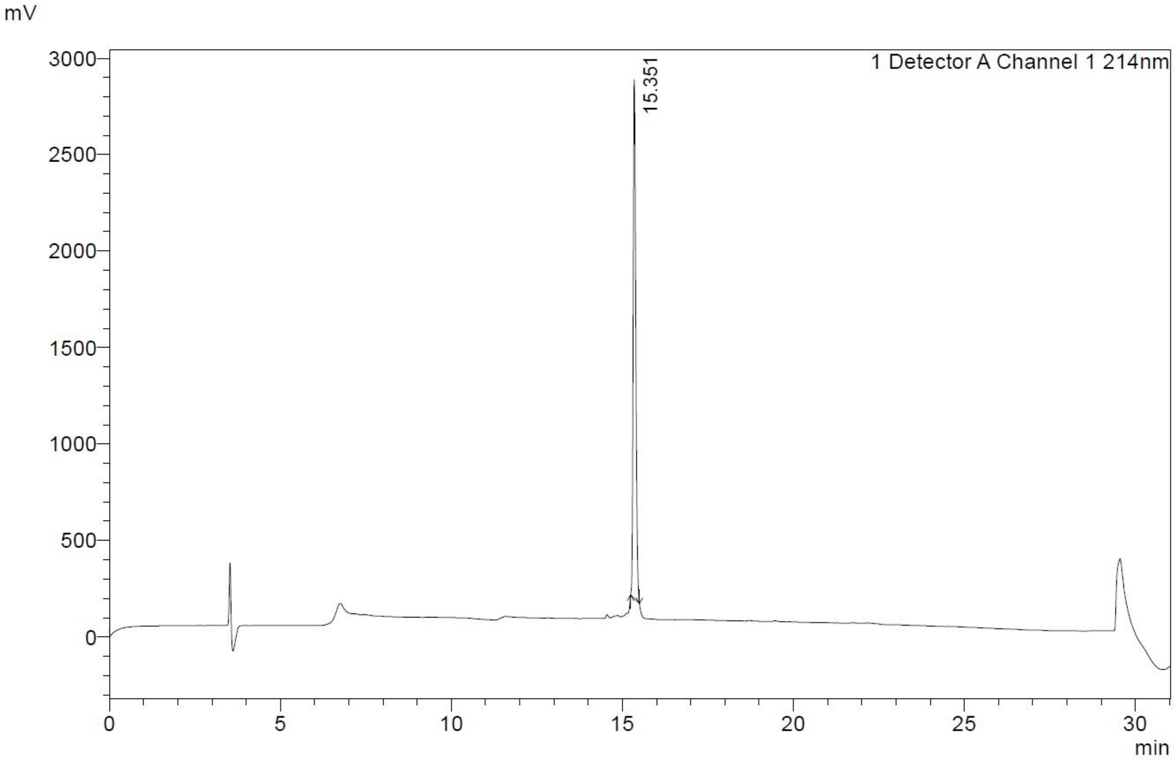

Fig. S31. HPLC trace showing the reinjection of purified LL(Ala5)-NH_2 (**33**). The peptide eluted as a single peak at 15.35 min using the HPLC **method E** outlined in part III.

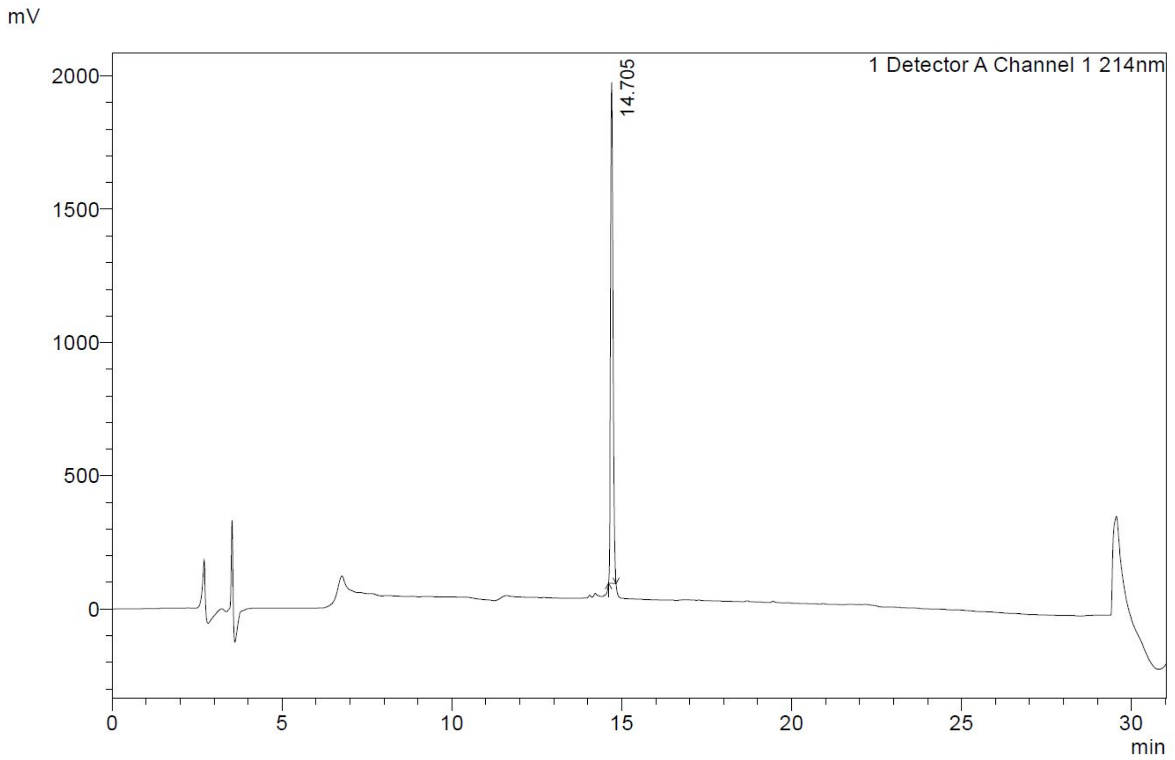

Fig. S32. HPLC trace showing the reinjection of purified LL-(Ala6)-NH_2 (**34**). The peptide eluted as a single peak at 14.71 min using the HPLC **method E** outlined in part III.

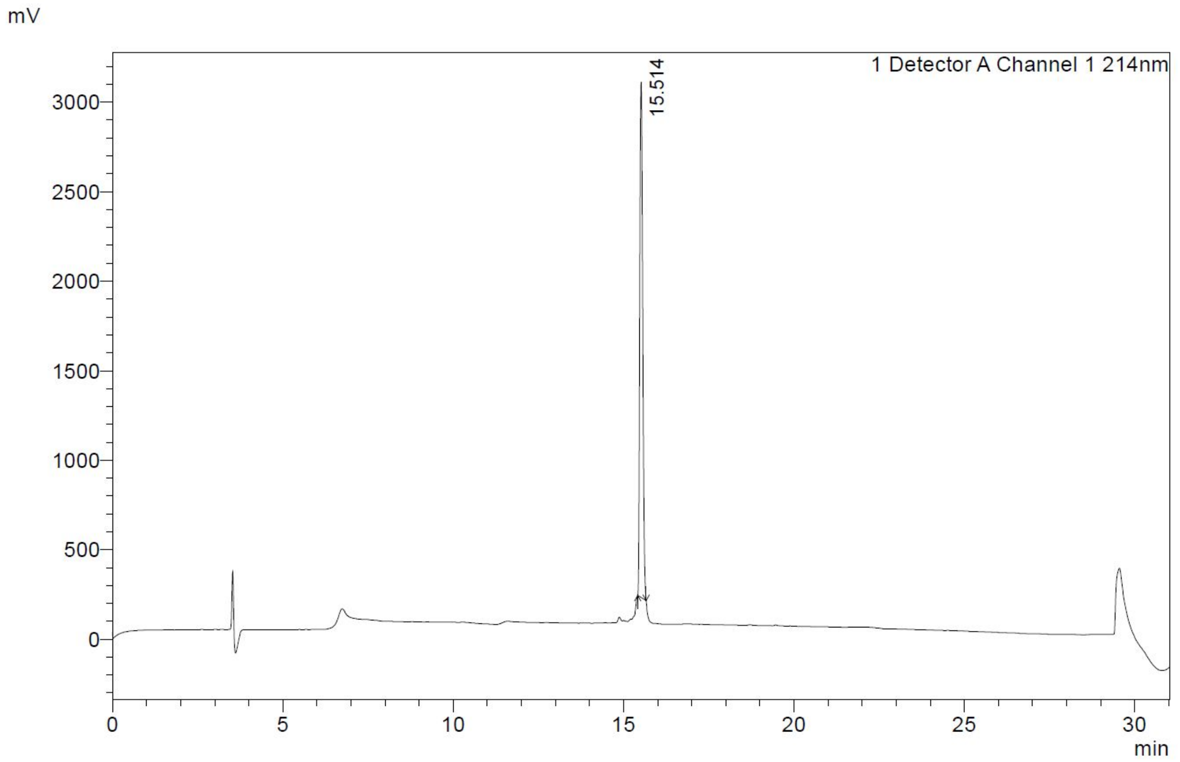

Fig. S33. HPLC trace showing the reinjection of purified LL(D-Ala7)-NH_2 (**35**). The peptide eluted as a single peak at 15.51 min using the HPLC **method E** outlined in part III.

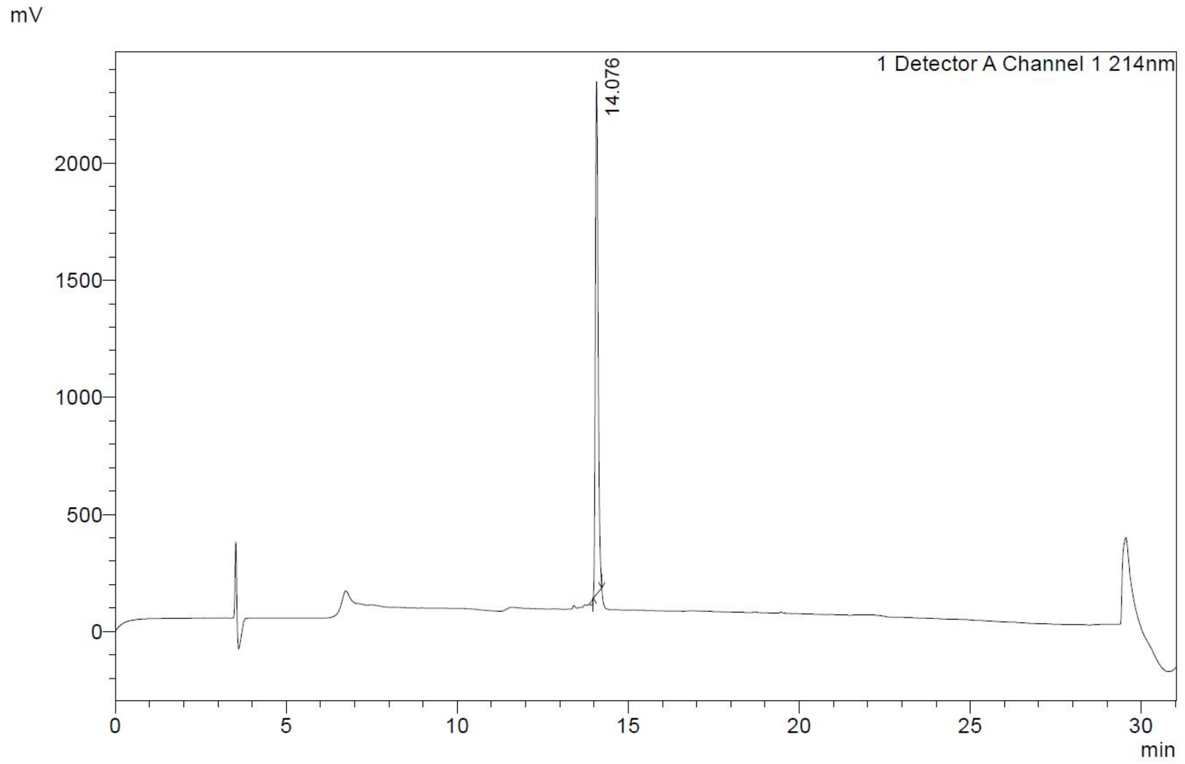

Fig. S34. HPLC trace showing the reinjection of purified LL(Ala8)-NH_2 (**36**). The peptide eluted as a single peak at 14.08 min using the HPLC **method E** outlined in part III.

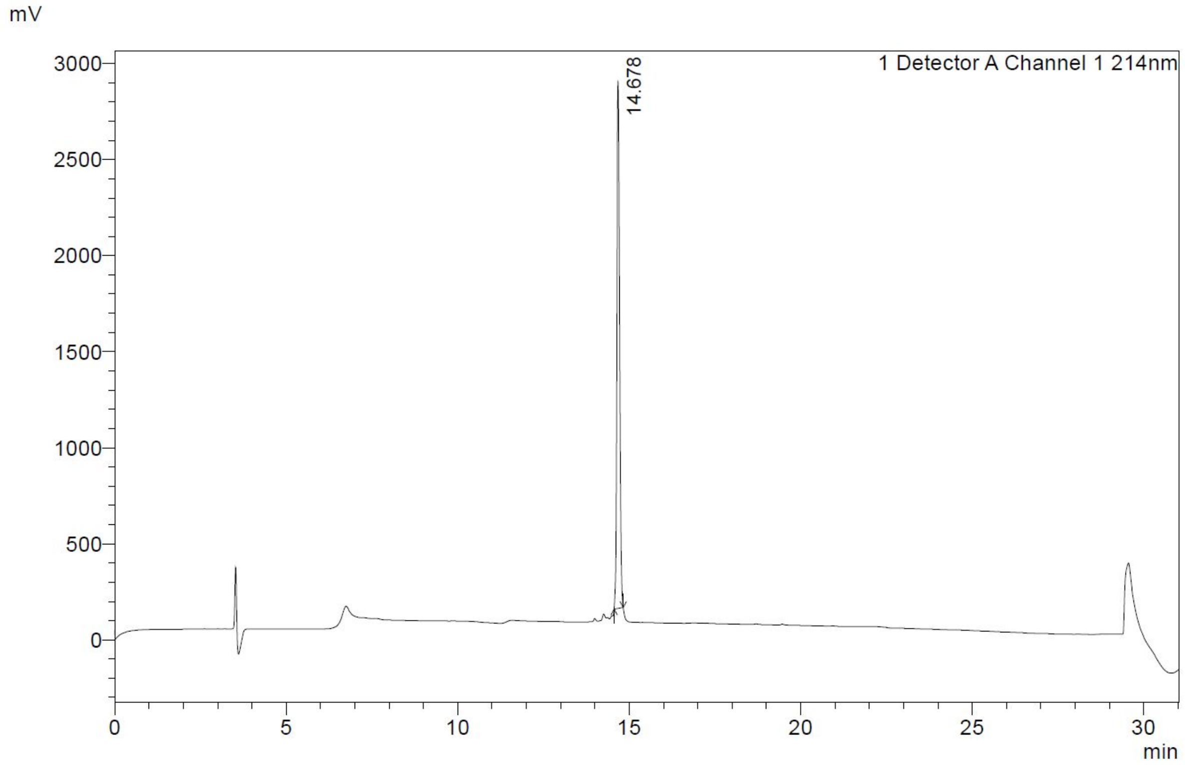

Fig. S35. HPLC trace showing the reinjection of purified LL(Ala9)-NH_2 (**37**). The peptide eluted as a single peak at 14.68 min using the HPLC **method E** outlined in part III.

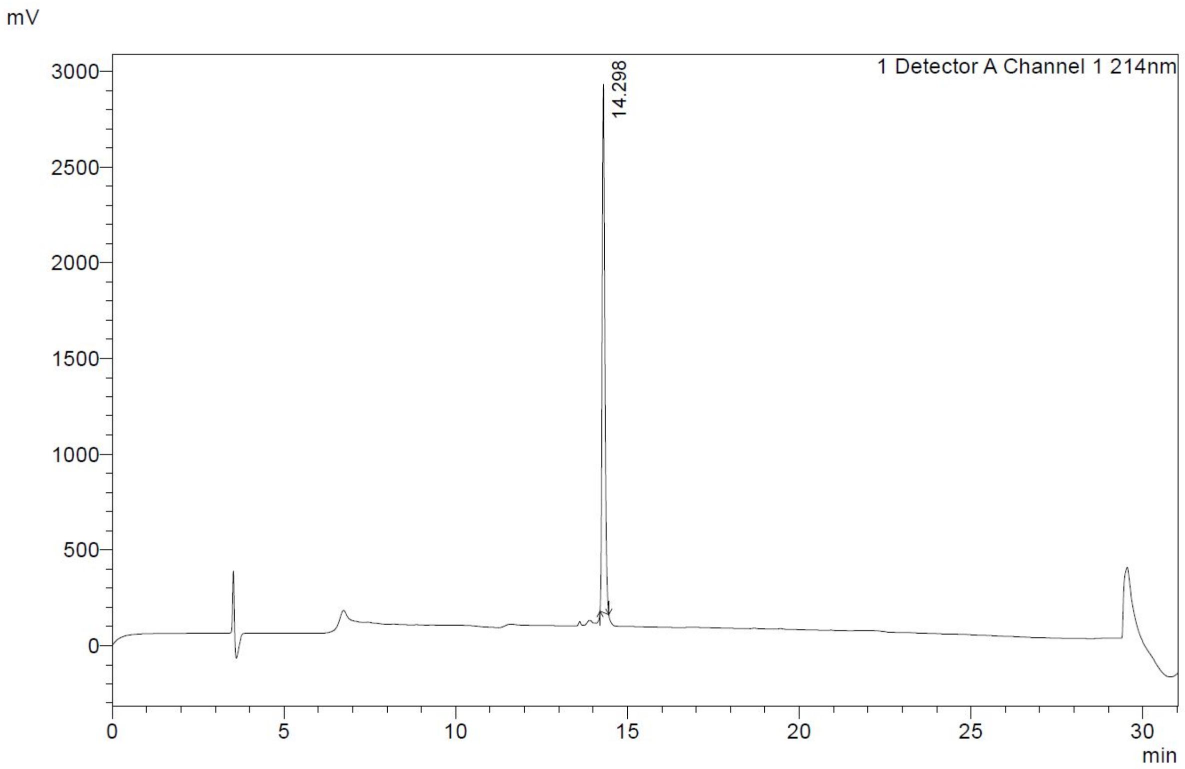

Fig. S36. HPLC trace showing the reinjection of purified LL(Ala10)-NH_2 (**38**). The peptide eluted as a single peak at 14.30 min using the HPLC **method E** outlined in part III.

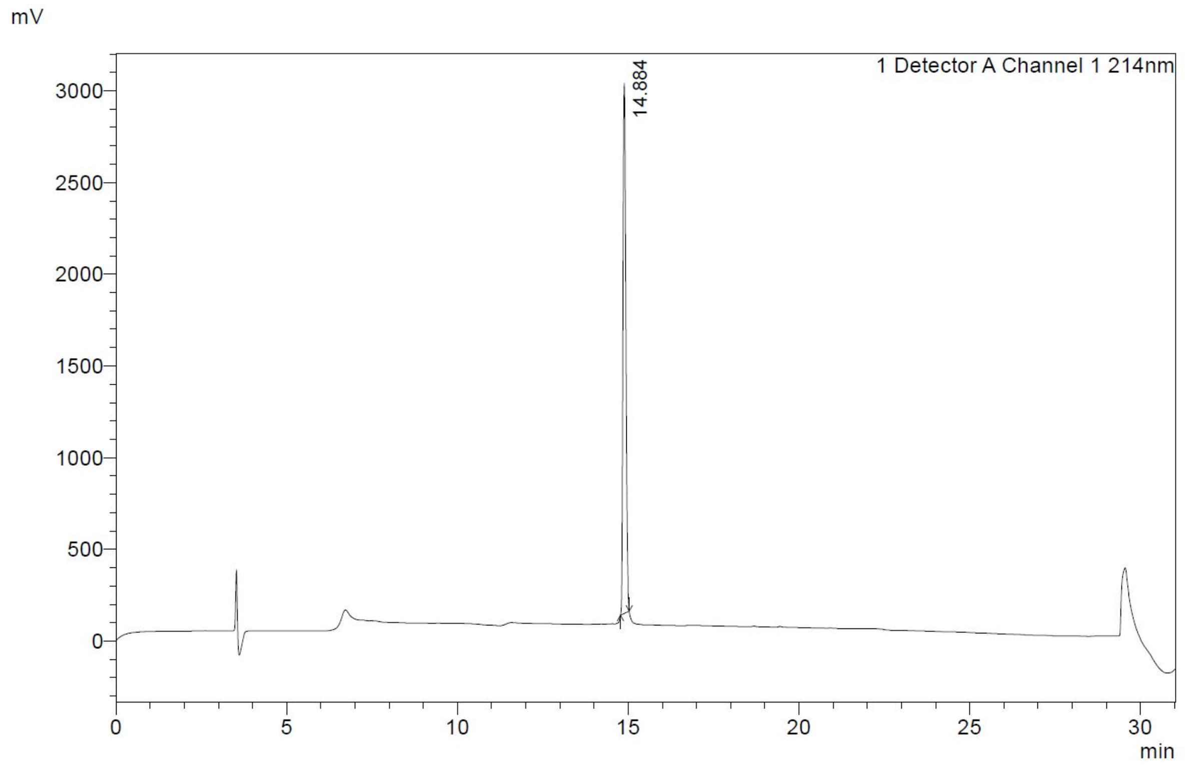

Fig. S37. HPLC trace showing the reinjection of purified LL(Ala11)-NH_2 (**39**). The peptide eluted as a single peak at 14.88 min using the HPLC **method E** outlined in part III.

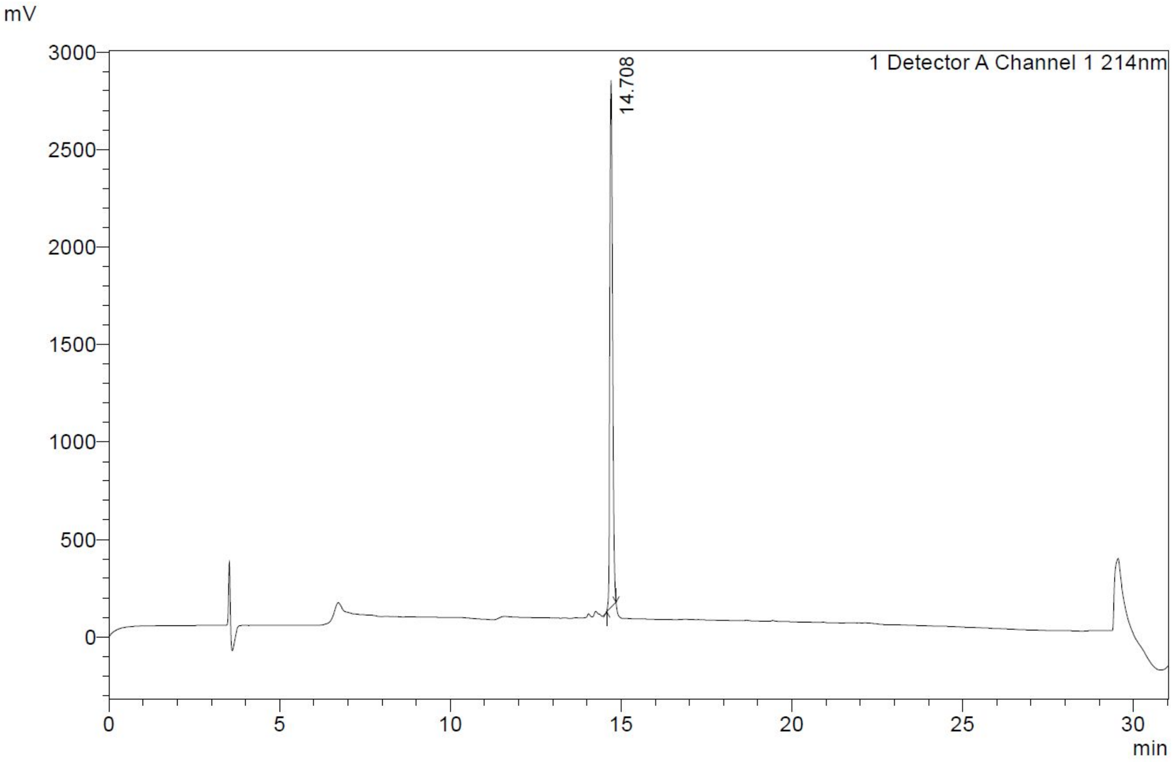

Fig. S38. HPLC trace showing the reinjection of purified LL(Ala12)-NH_2 (**40**). The peptide eluted as a single peak at 14.71 min using the HPLC **method E** outlined in part III.

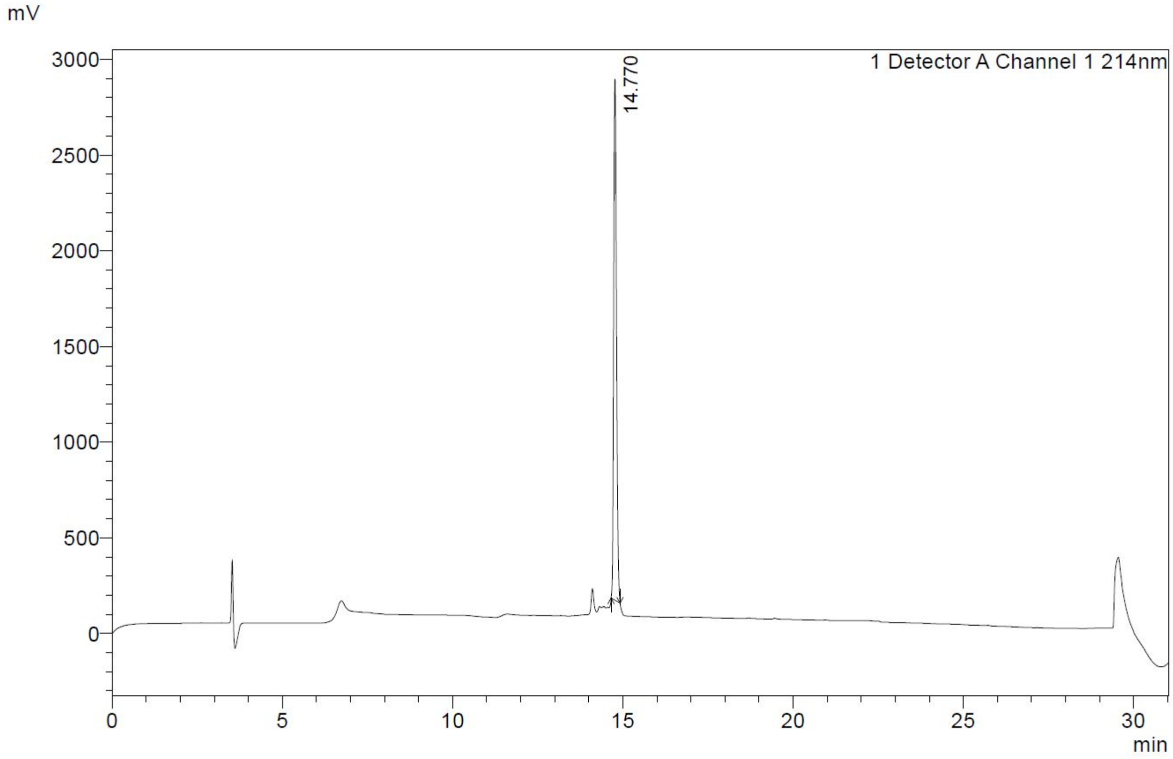

Fig. S39. HPLC trace showing the reinjection of purified LL(Ala13)-NH_2 (**41**). The peptide eluted as a single peak at 14.77 min using the HPLC **method E** outlined in part III.

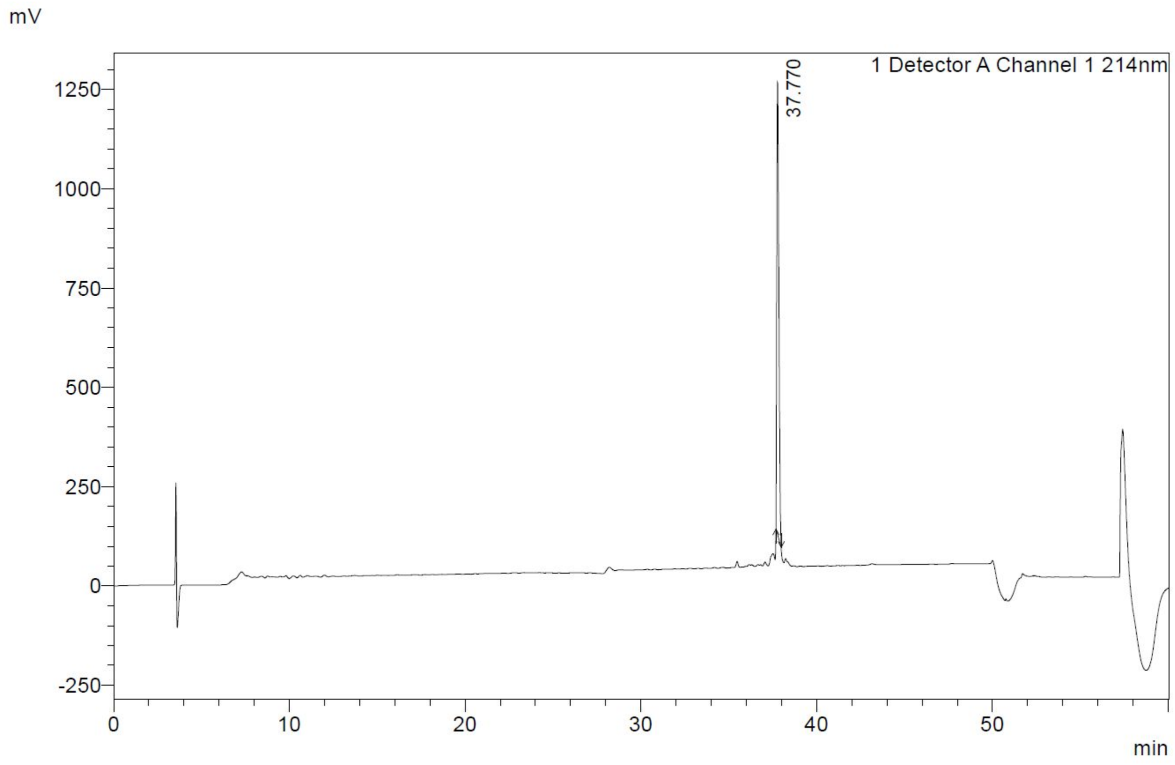

Fig. S40. HPLC trace showing the reinjection of purified LL(Leu9)-NH_2 (**42**). The peptide eluted as a single peak at 37.77 min using the HPLC **method D** outlined in part III.

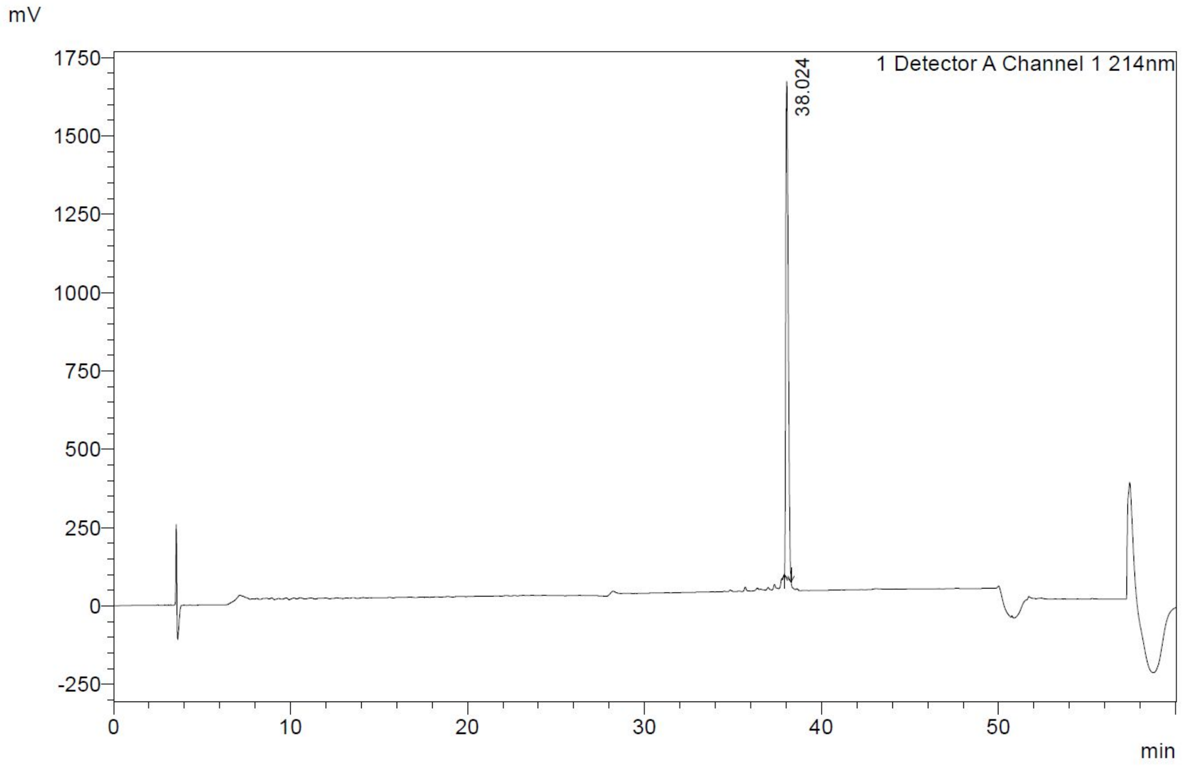

Fig. S41. HPLC trace showing the reinjection of purified LL(Phe9)-NH_2 (**43**). The peptide eluted as a single peak at 38.02 min using the HPLC **method D** outlined in part III.

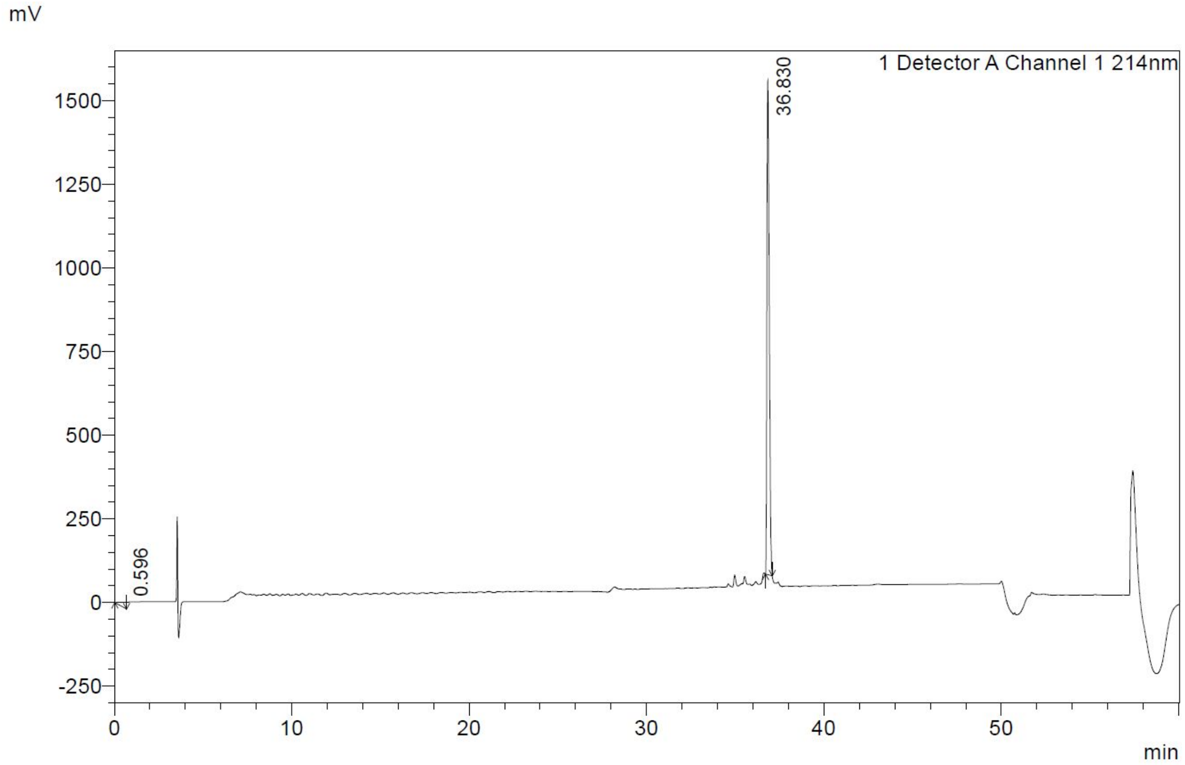

Fig. S42. HPLC trace showing the reinjection of purified LL(Met9)-NH_2 (**44**). The peptide eluted as a single peak at 36.83 min using the HPLC **method D** outlined in part III.

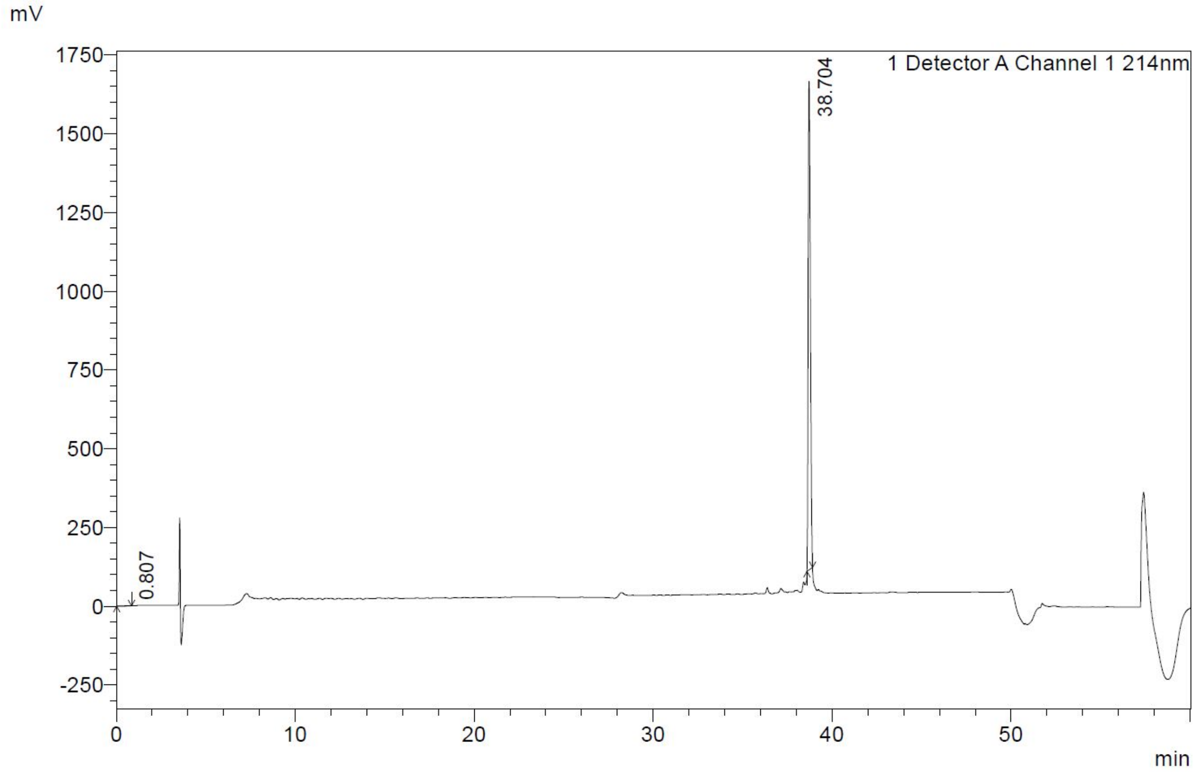

Fig. S43. HPLC trace showing the reinjection of purified LL(Trp9)-NH_2 (**45**). The peptide eluted as a single peak at 35.70 min using the HPLC **method D** outlined in part III.

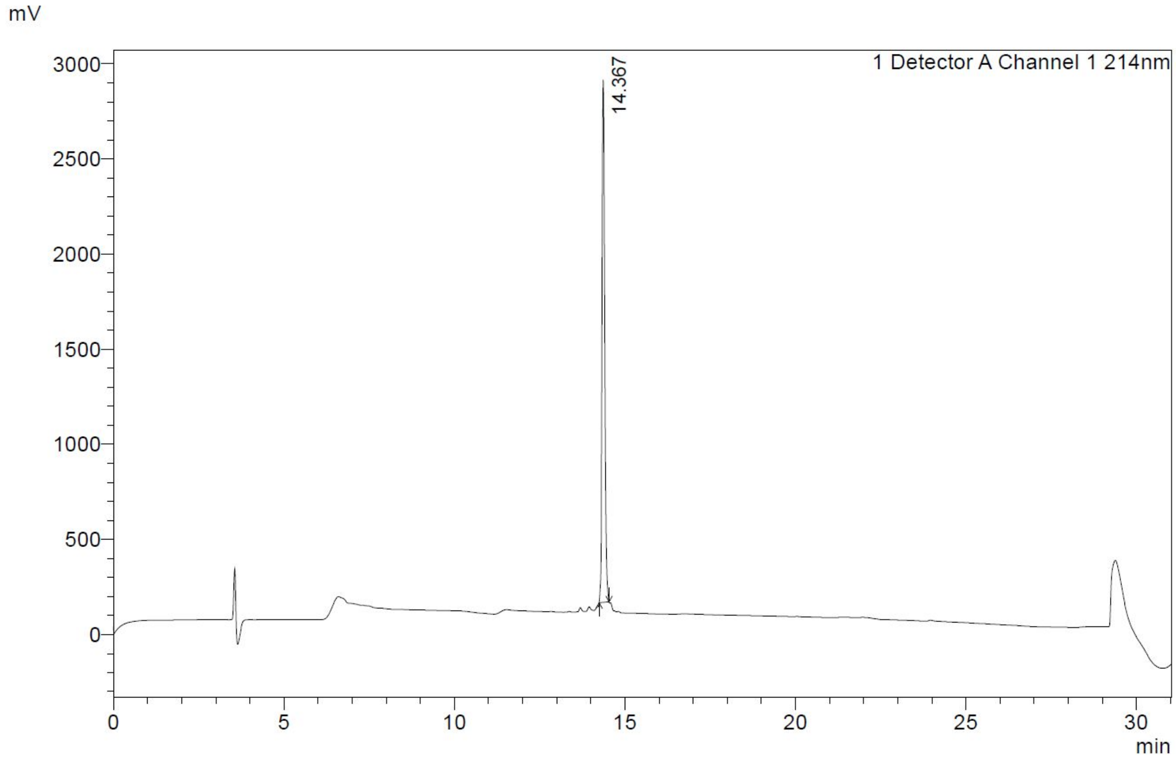

Fig. S44. HPLC trace showing the reinjection of purified LL(Ser9)-NH_2 (**46**). The peptide eluted as a single peak at 14.37 min using the HPLC **method E** outlined in part III.

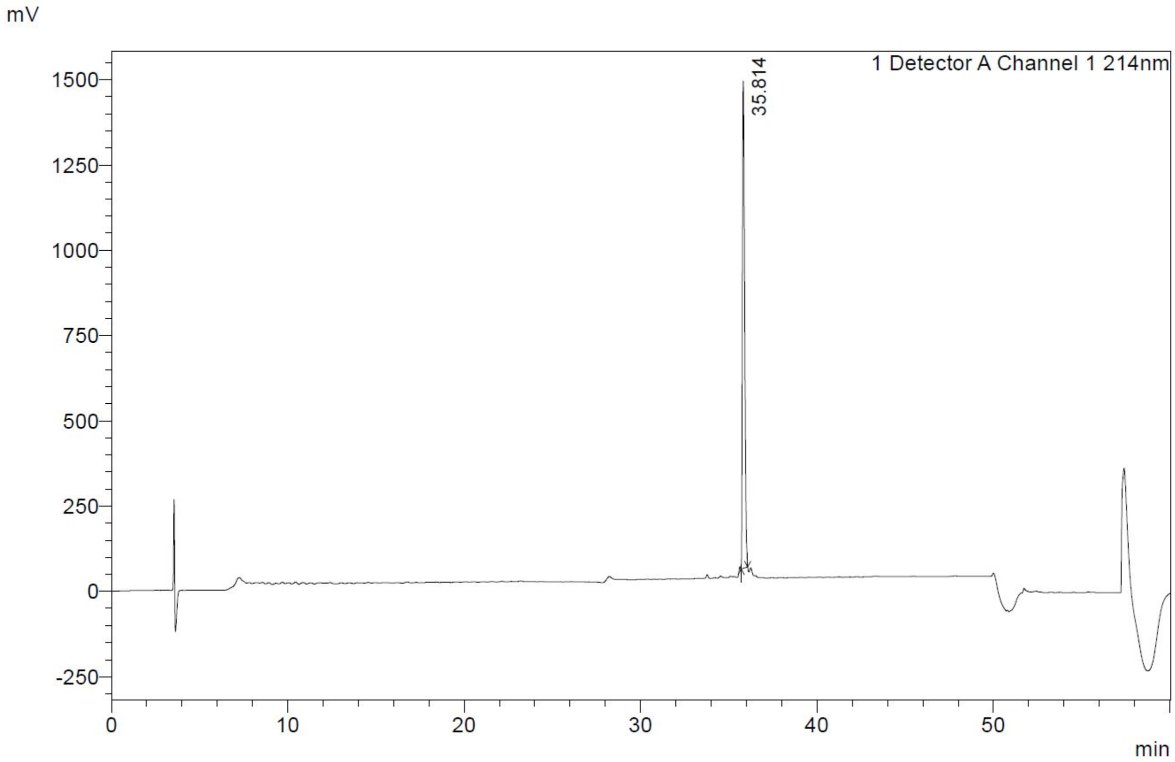

Fig. S45. HPLC trace showing the reinjection of purified LL(Asn9)-NH_2 (**47**). The peptide eluted as a single peak at 35.81 min using the HPLC **method D** outlined in part III.

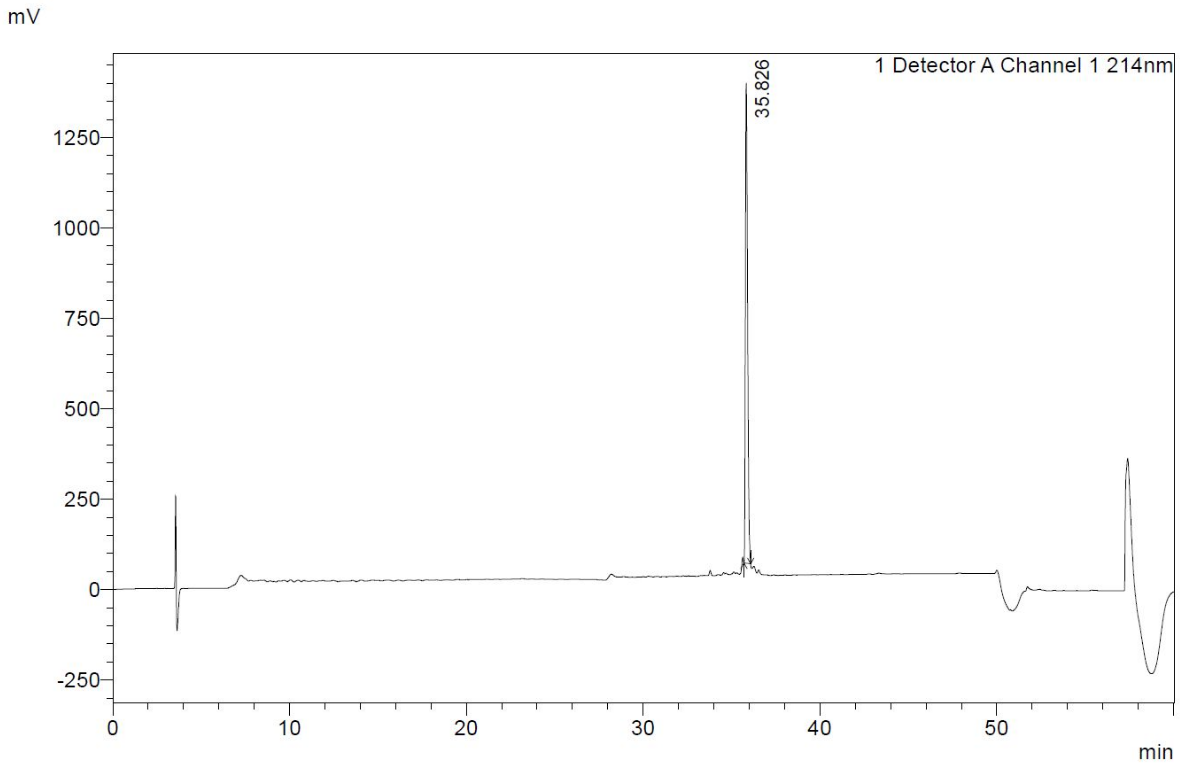

Fig. S46. HPLC trace showing the reinjection of purified LL(Gln9)-NH_2 (**48**). The peptide eluted as a single peak at 35.83 min using the HPLC **method D** outlined in part III.

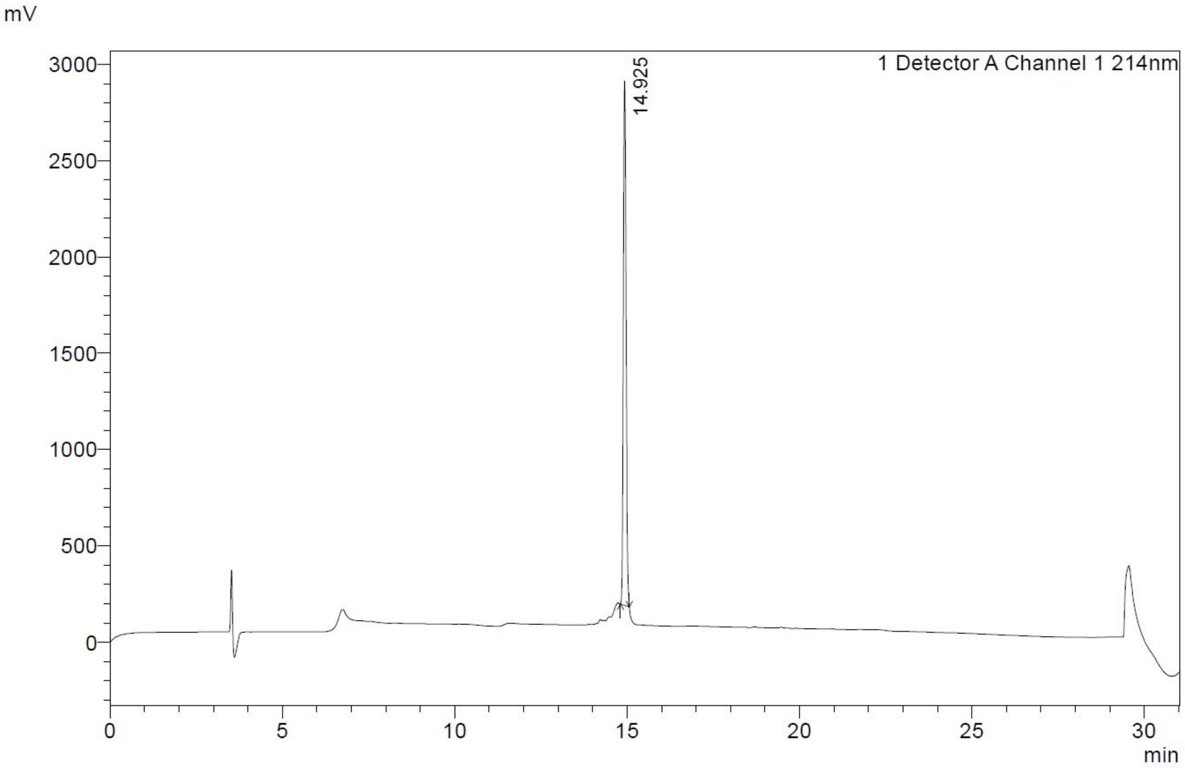

Fig. S47. HPLC trace showing the reinjection of purified LL(MeAbu9)-NH_2 (**49**). The peptide eluted as a single peak at 14.93 min using the HPLC **method E** outlined in part III.

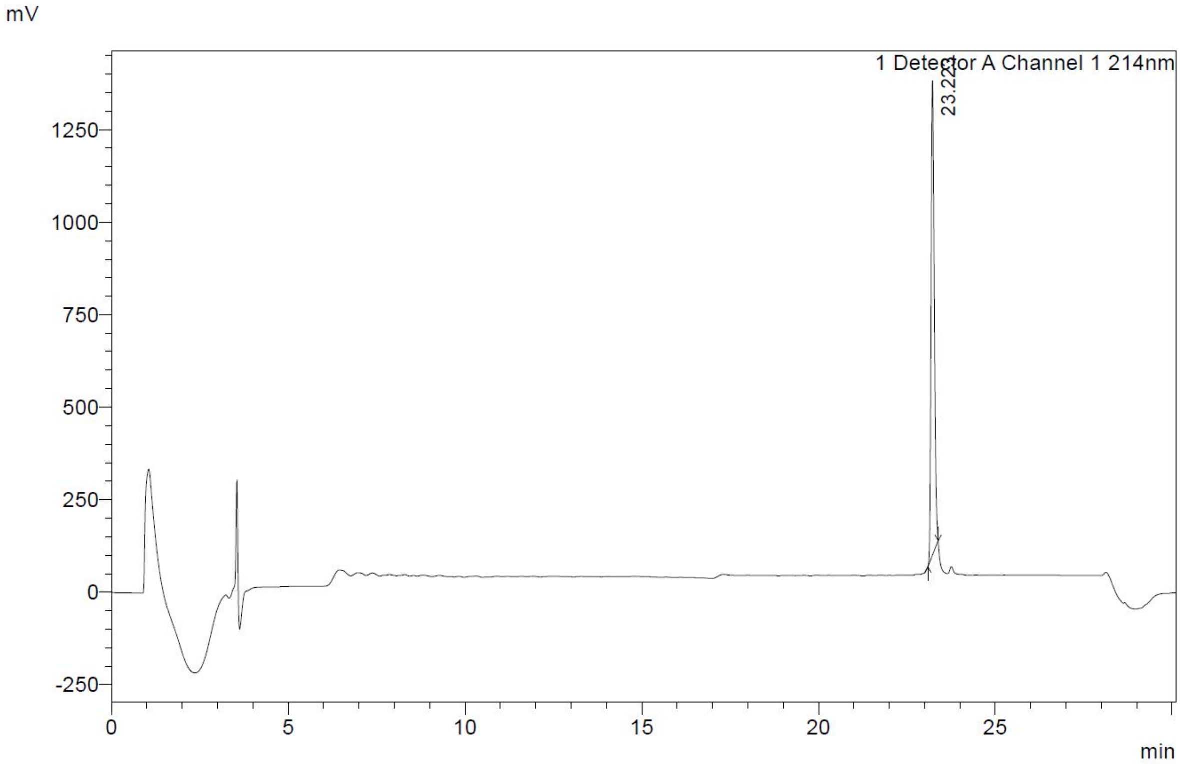

Fig. S48. HPLC trace showing the reinjection of purified LL(Dap[Alloc]9)-NH_2 (**50**). The peptide eluted as a single peak at 23.22 min using the HPLC **method E** outlined in part III.

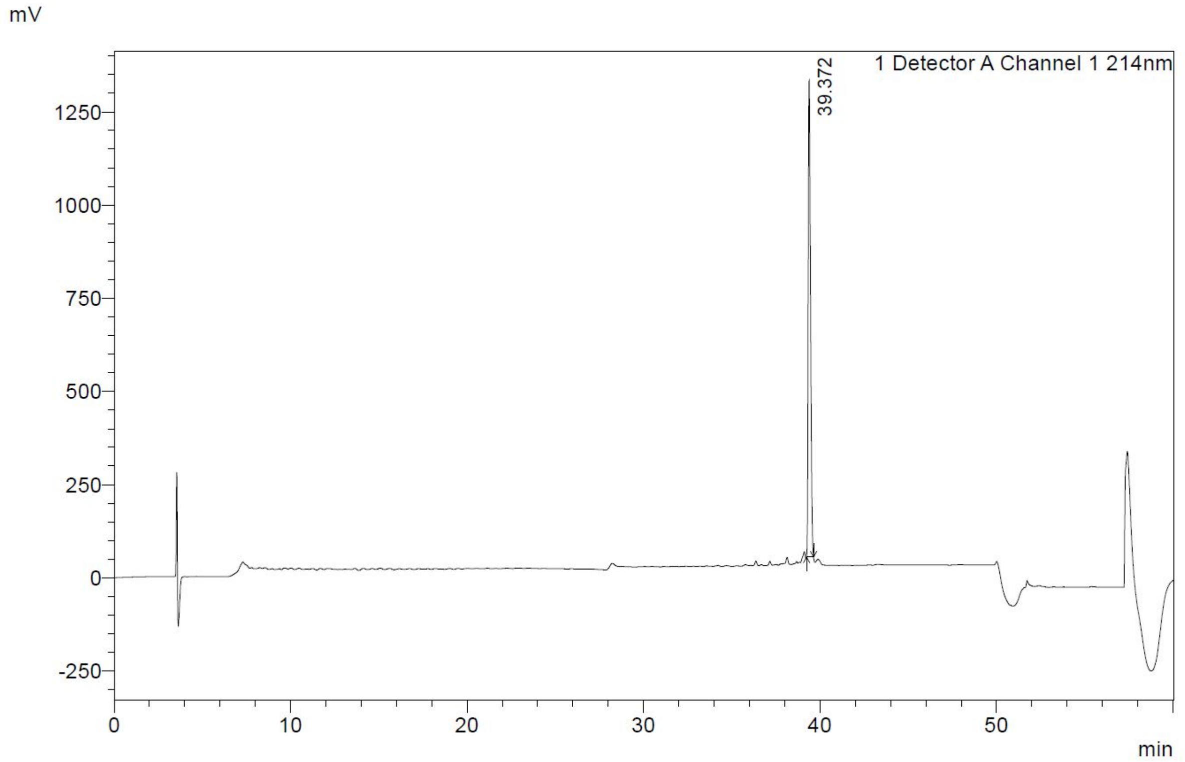

Fig. S49. HPLC trace showing the reinjection of purified LL(Glu[OAll]9)-NH_2 (**51**). The peptide eluted as a single peak at 39.37 min using the HPLC **method D** outlined in part III.

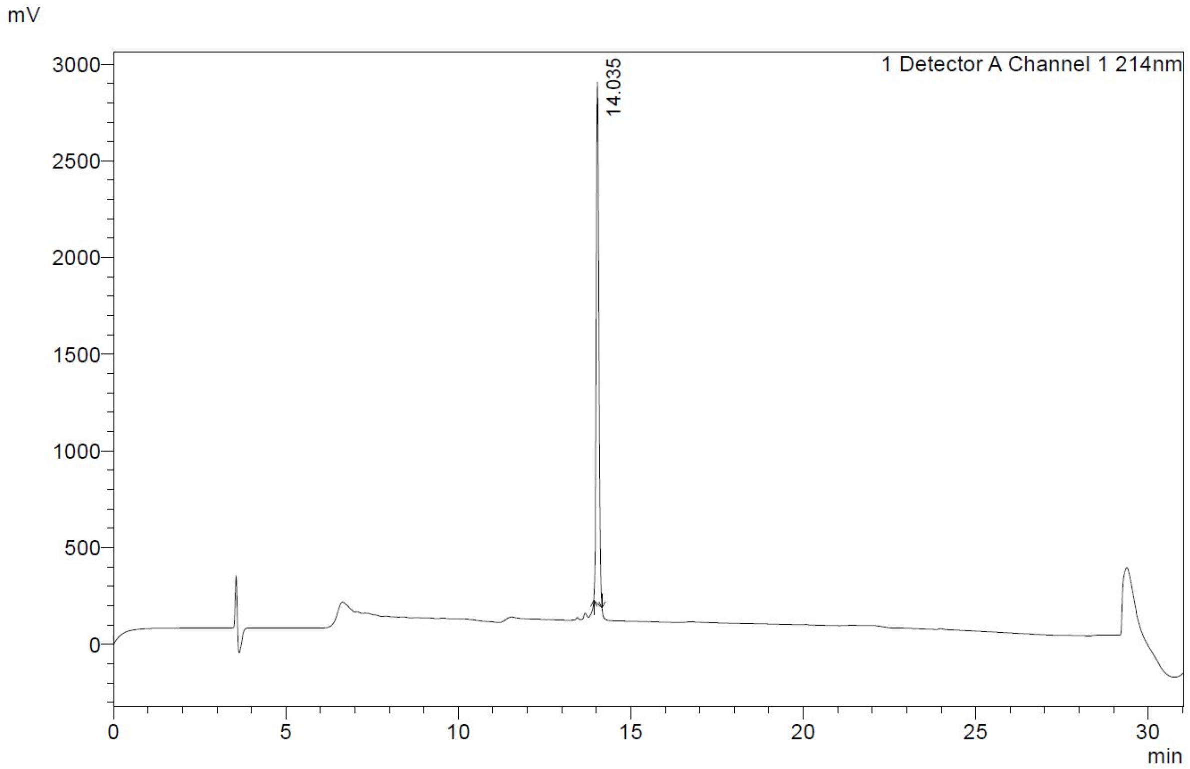

Fig. S50. HPLC trace showing the reinjection of purified LL(Dap9)-NH_2 (**52**). The peptide eluted as a single peak at 14.04 min using the HPLC **method E** outlined in part III.

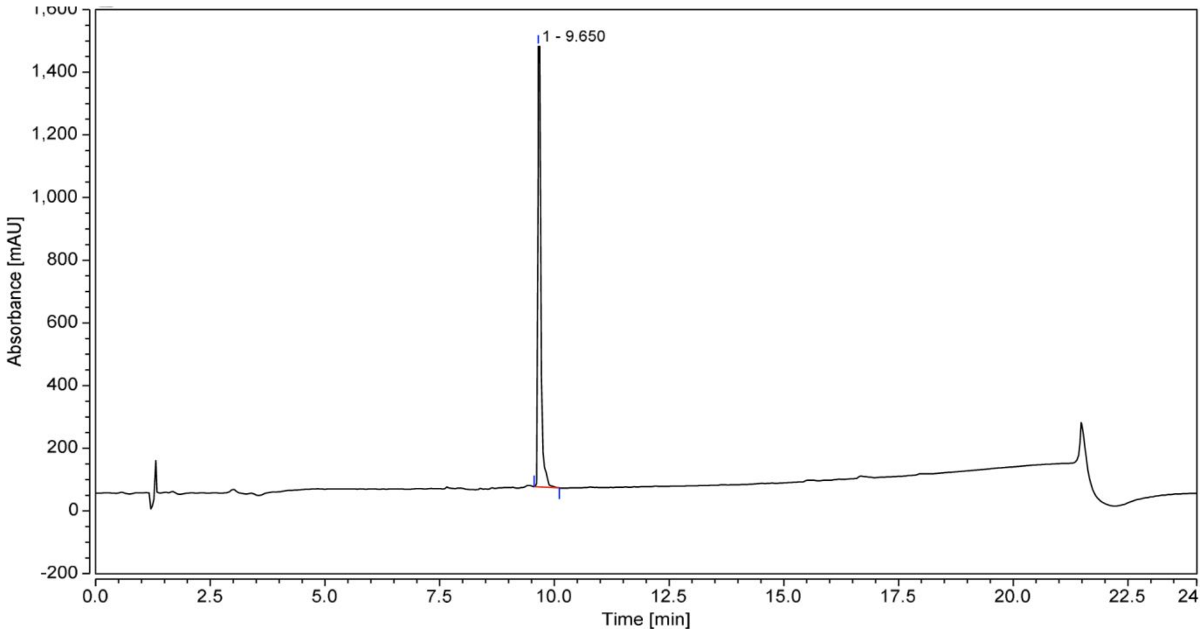

Fig. S51. HPLC trace showing the reinjection of purified Δ12LB(Leu9)-NH_2 (**53**). The peptide eluted as a single peak at 9.65 min using the HPLC **method C** outlined in part III.

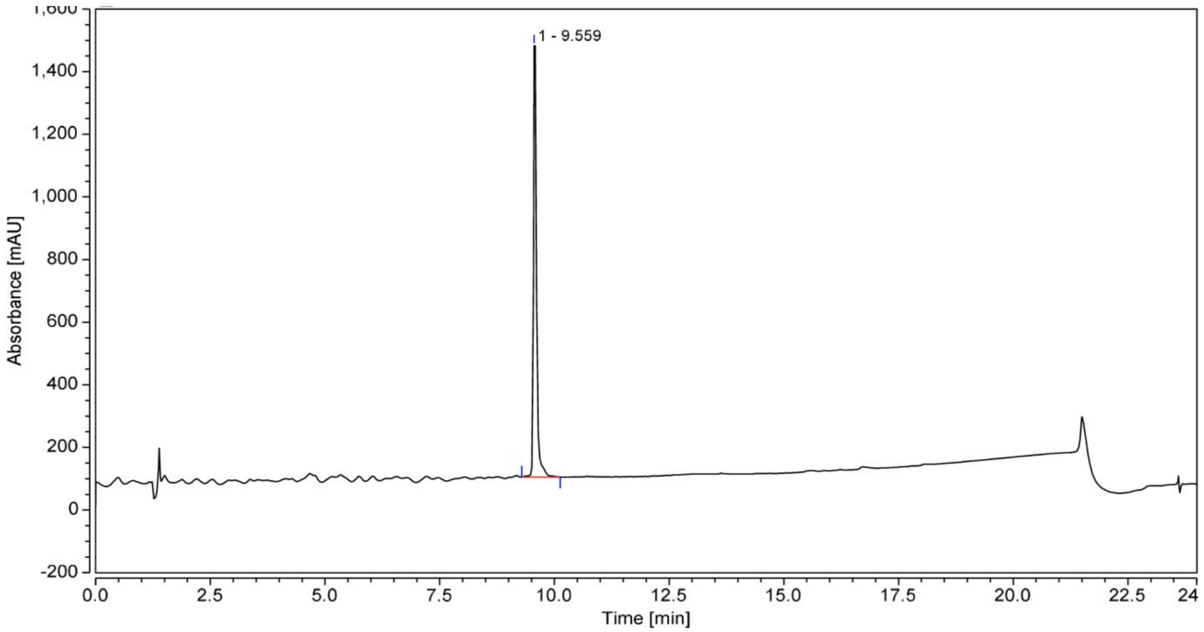

Fig. S52. HPLC trace showing the reinjection of purified Δ12LB(Phe9)-NH_2 (**54**). The peptide eluted as a single peak at 9.56 min using the HPLC **method C** outlined in part III.

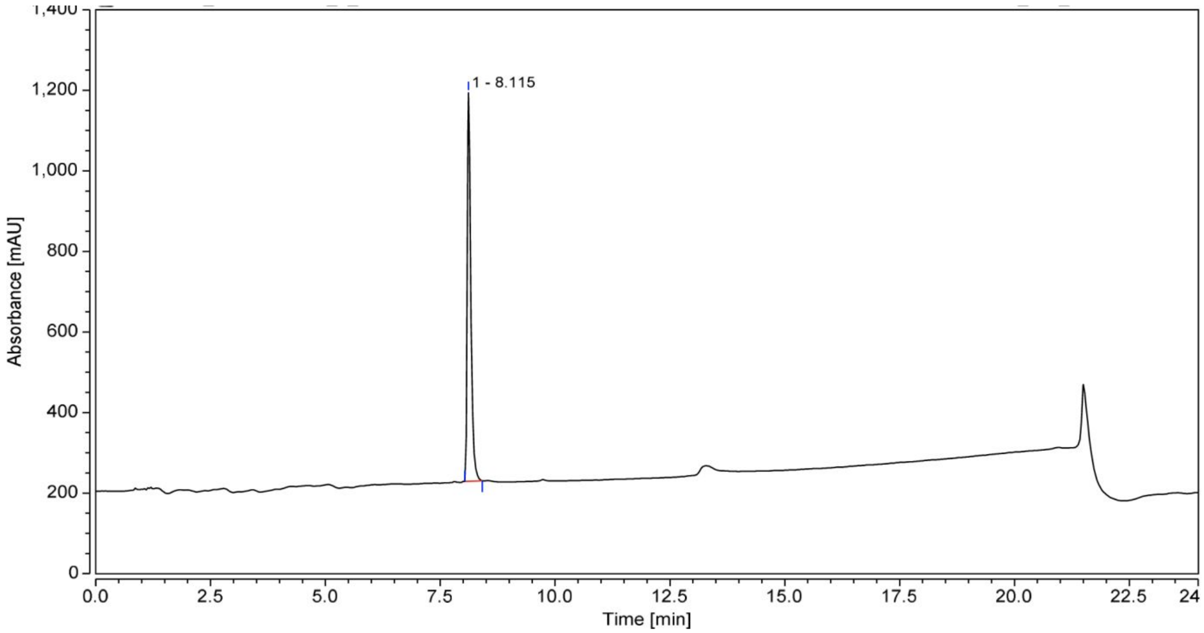

Fig. S53. HPLC trace showing the reinjection of purified Δ12LB(Met9)-NH_2 (**55**). The peptide eluted as a single peak at 8.12 min using the HPLC **method C** outlined in part III.

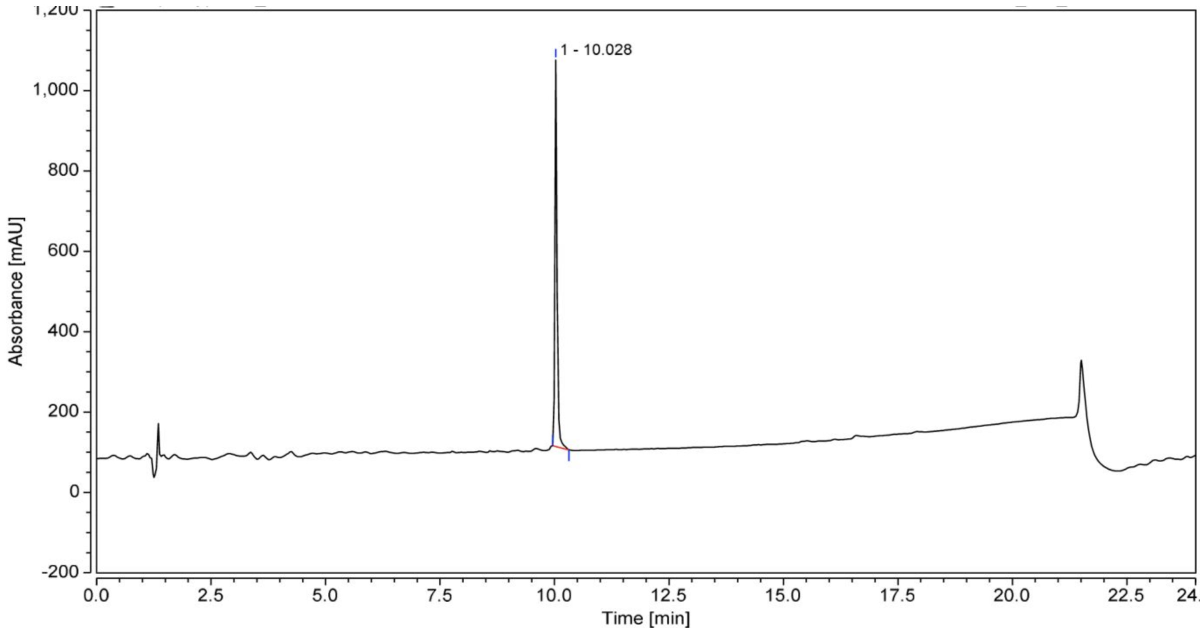

Fig. S54. HPLC trace showing the reinjection of purified Δ12LB(Trp9)-NH_2 (**56**). The peptide eluted as a single peak at 10.03 min using the HPLC **method C** outlined in part III.

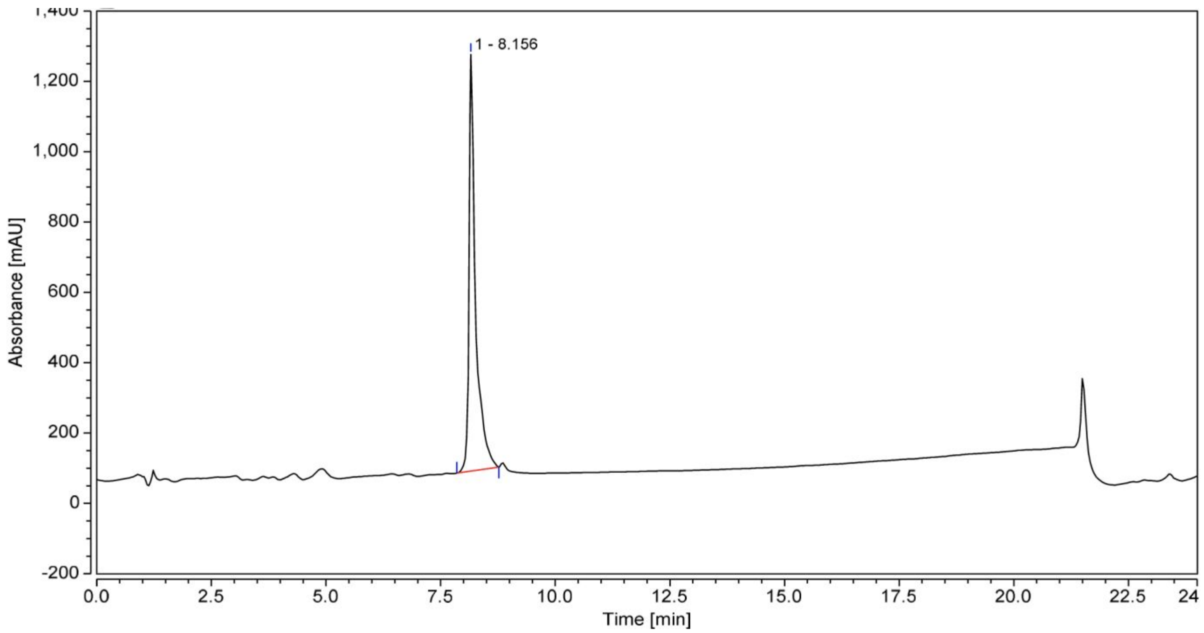

Fig. S55. HPLC trace showing the reinjection of purified Δ12LB(Ser9)-NH_2 (**57**). The peptide eluted as a single peak at 8.16 min using the HPLC **method C** outlined in part III.

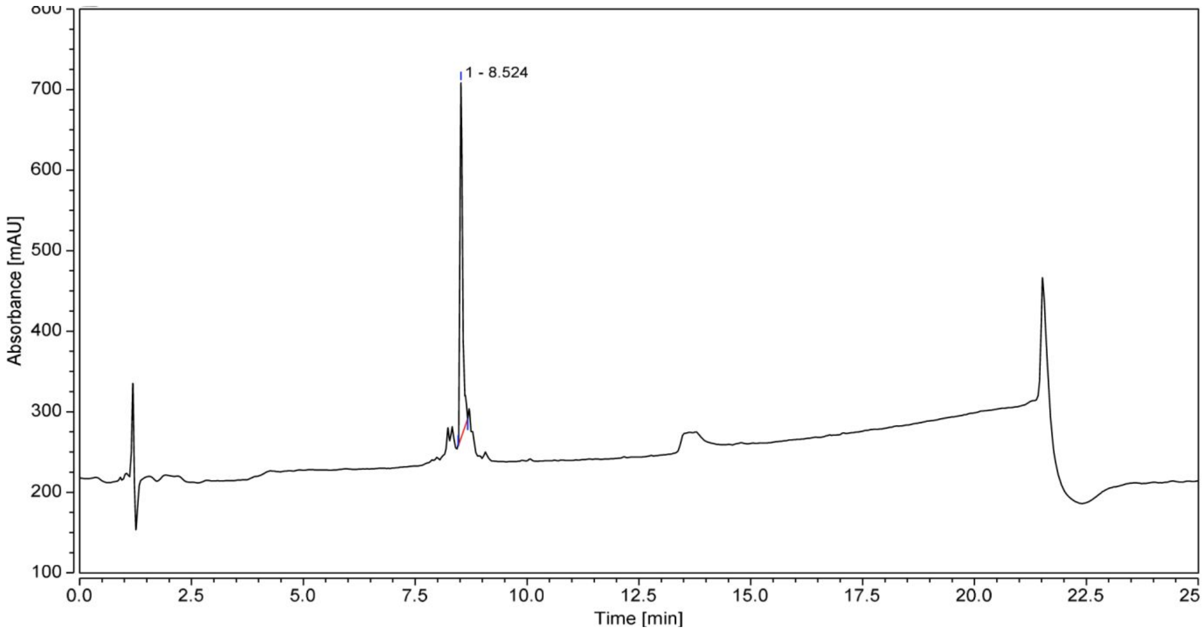

Fig. S56. HPLC trace showing the reinjection of purified Δ12LB(Asn9)-NH_2 (**58**). The peptide eluted as a single peak at 8.52 min using the HPLC **method C** outlined in part III.

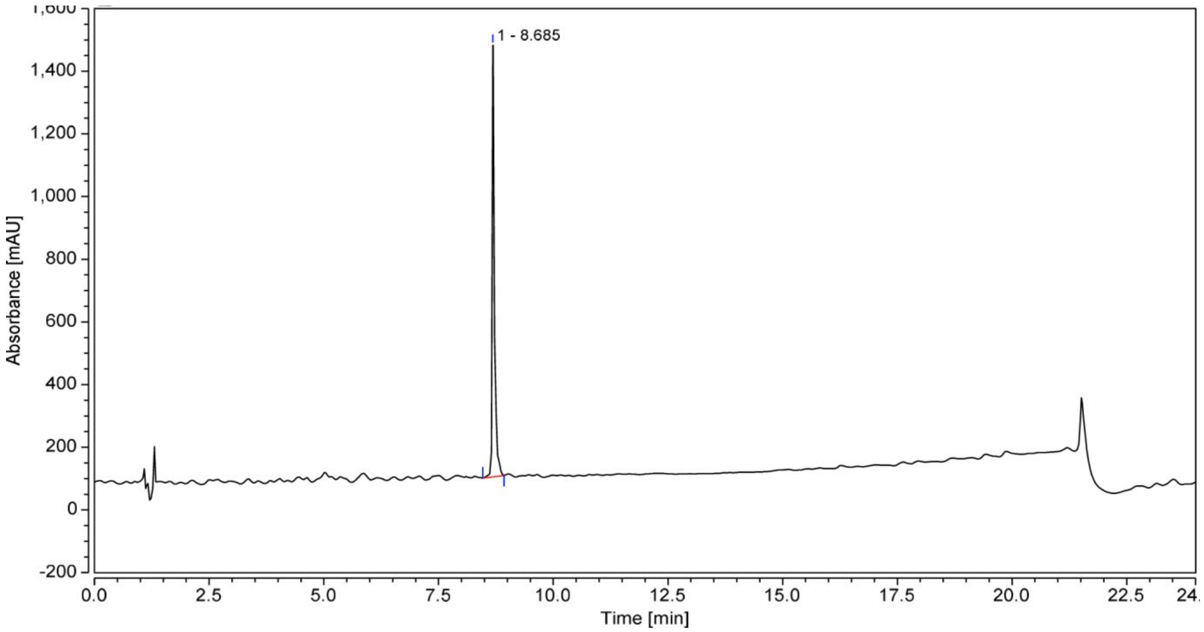

Fig. S57. HPLC trace showing the reinjection of purified Δ12LB(Gln9)-NH_2 (**59**). The peptide eluted as a single peak at 8.69 min using the HPLC **method C** outlined in part III.

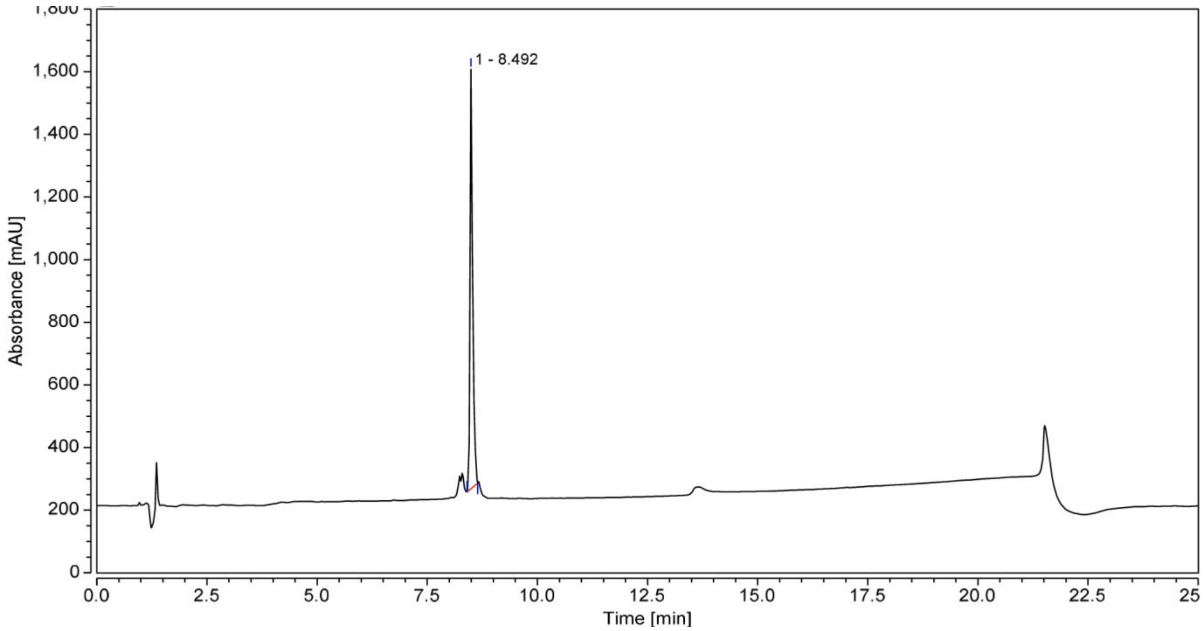

Fig. S58. HPLC trace showing the reinjection of purified Δ12LB(MeAbu9)-NH_2 (**60**). The peptide eluted as a single peak at 8.49 min using the HPLC **method C** outlined in part III.

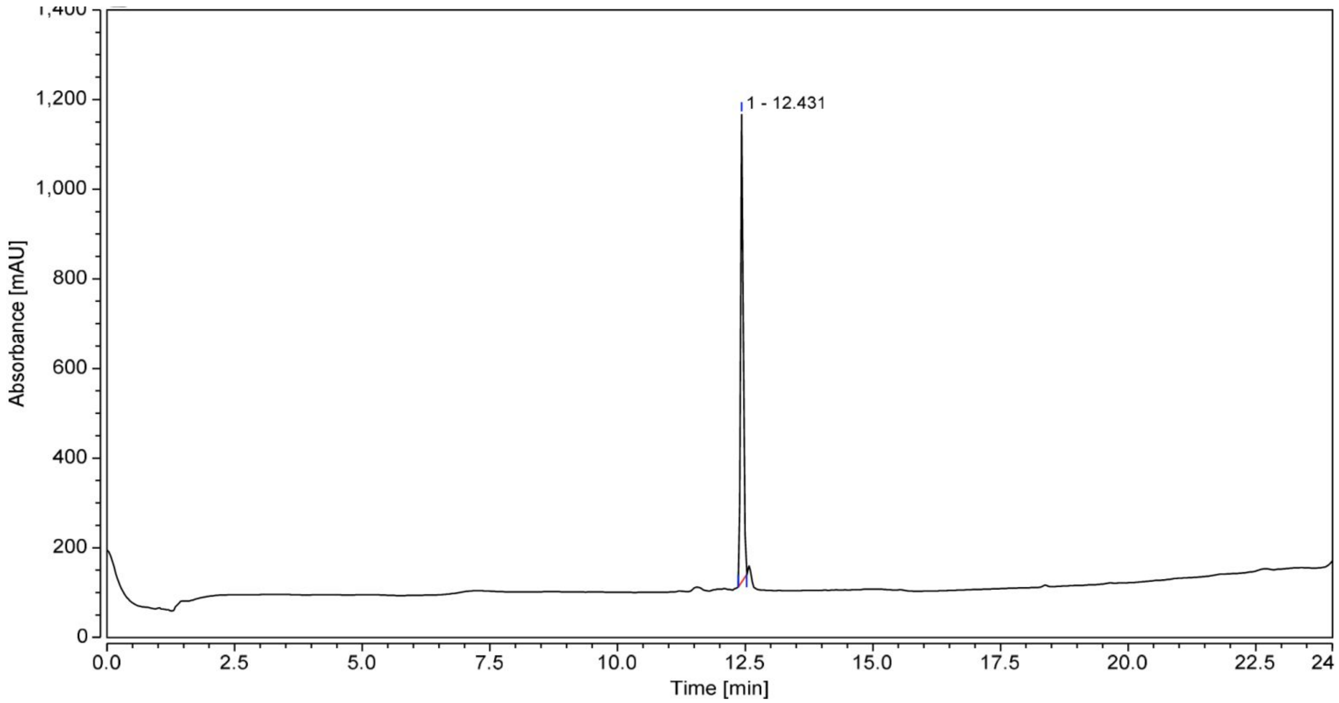

Fig. S59. HPLC trace showing the reinjection of purified Δ12LB(Dap[Alloc]9)-NH_2 (**61**). The peptide eluted as a single peak at 12.43 min using the HPLC **method C** outlined in part III.

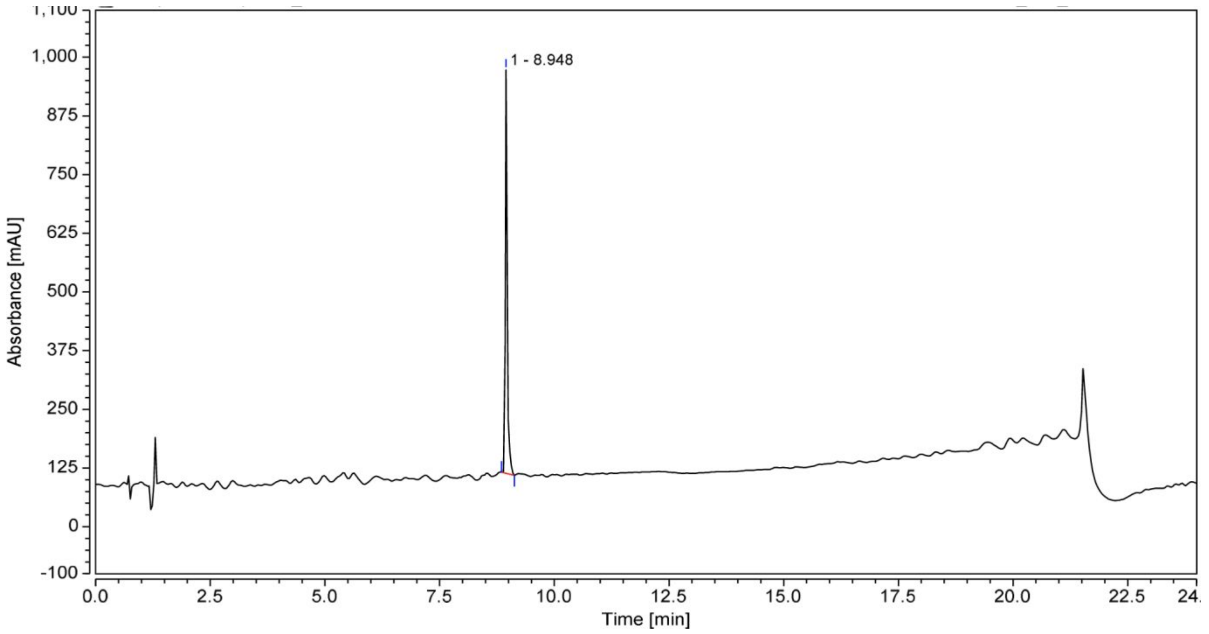

Fig. S60. HPLC trace showing the reinjection of purified Δ12LB(Glu[OAll]9)-NH_2 (**62**). The peptide eluted as a single peak at 8.95 min using the HPLC **method C** outlined in part III.

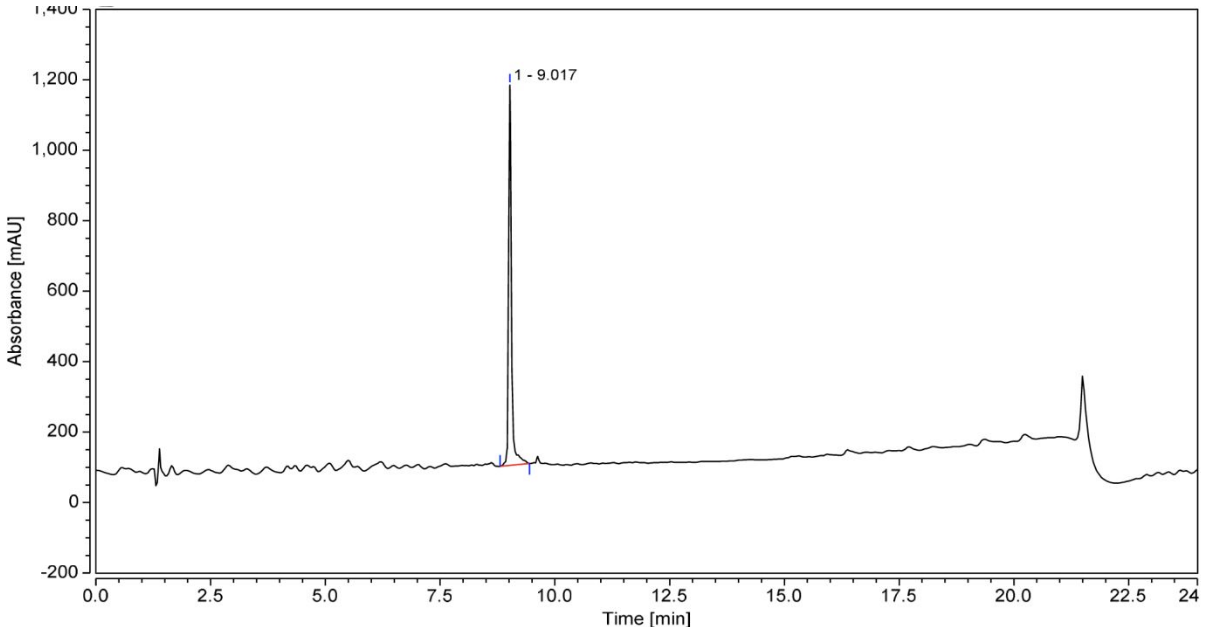

Fig. S61. HPLC trace showing the reinjection of purified Δ12LB(Asp9)-NH_2 (**63**). The peptide eluted as a single peak at 9.02 min using the HPLC **method C** outlined in part III.

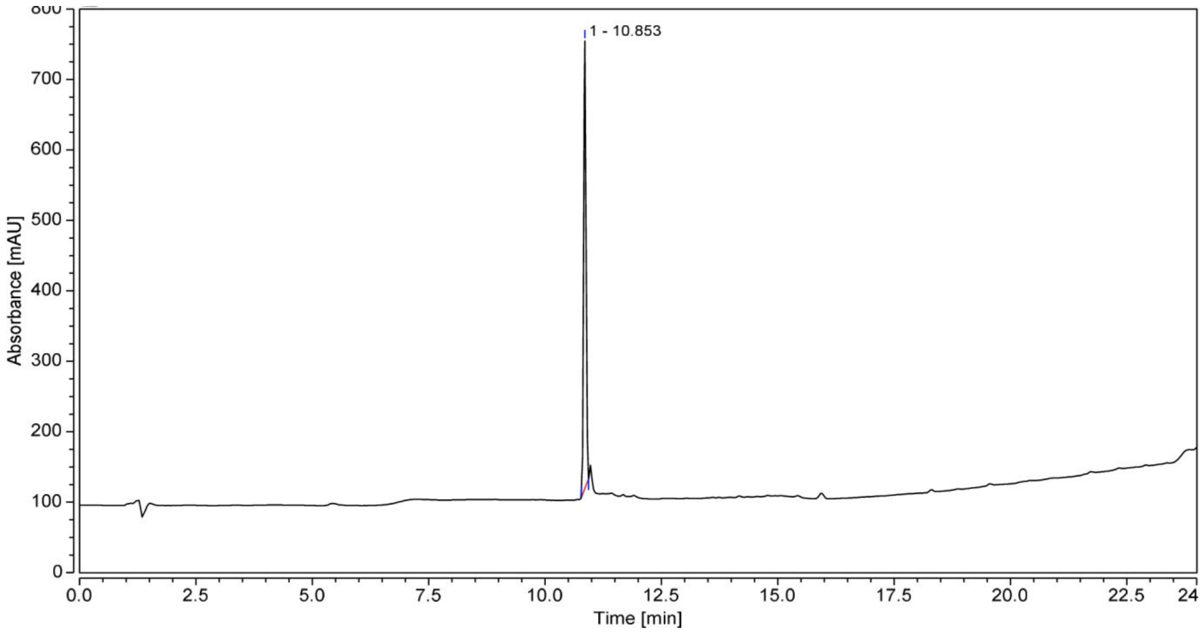

Fig. S62. HPLC trace showing the reinjection of purified Δ12LB(Dap9)-NH_2 (**64**). The peptide eluted as a single peak at 10.85 min using the HPLC **method C** outlined in part III.
